# Prophylactic and therapeutic treatment with a synthetic analogue of a parasitic worm product prevents experimental arthritis and inhibits IL-1β production via NRF2-mediated counter-regulation of the inflammasome

**DOI:** 10.1016/j.jaut.2015.04.005

**Published:** 2015-06

**Authors:** Justyna Rzepecka, Miguel A. Pineda, Lamyaa Al-Riyami, David T. Rodgers, Judith K. Huggan, Felicity E. Lumb, Abedawn I. Khalaf, Paul J. Meakin, Marlene Corbet, Michael L. Ashford, Colin J. Suckling, Margaret M. Harnett, William Harnett

**Affiliations:** a Strathclyde Institute of Pharmacy and Biomedical Sciences, University of Strathclyde, Glasgow G4 0NR, UK; b Institute of Infection, Immunity and Inflammation, University of Glasgow, Glasgow G12 8TA, UK; c Department of Pure and Applied Chemistry, University of Strathclyde, Glasgow G1 1Xl, UK; d Division of Cardiovascular & Diabetes Medicine, Medical Research Institute, Ninewells Hospital and Medical School, Dundee DD1 9SY, UK

**Keywords:** Arthritis, ES-62, IL-1β, Inflammasome, NRF2, Parasitic worm

## Abstract

Rheumatoid arthritis (RA) remains a debilitating autoimmune condition as many patients are refractory to existing conventional and biologic therapies, and hence successful development of novel treatments remains a critical requirement. Towards this, we now describe a synthetic drug-like small molecule analogue, SMA-12b, of an immunomodulatory parasitic worm product, ES-62, which acts both prophylactically and therapeutically against collagen-induced arthritis (CIA) in mice. Mechanistic analysis revealed that SMA-12b modifies the expression of a number of inflammatory response genes, particularly those associated with the inflammasome in mouse bone marrow-derived macrophages and indeed IL-1β was the most down-regulated gene. Consistent with this, IL-1β was significantly reduced in the joints of mice with CIA treated with SMA-12b. SMA-12b also increased the expression of a number of genes associated with anti-oxidant responses that are controlled by the transcription factor NRF2 and critically, was unable to inhibit expression of IL-1β by macrophages derived from the bone marrow of NRF2^−/−^ mice. Collectively, these data suggest that SMA-12b could provide the basis of an entirely novel approach to fulfilling the urgent need for new treatments for RA.

## Highlights

• SMA-12b is a mimetic of the active PC-moiety of the helminth immunomodulator, ES-62.
• SMA-12b effectively protects mice against collagen-induced arthritis (CIA).
• SMA-12b downregulates IL-1β and inflammasome genes via activation of NRF2.
• SMA-12b reduces IL-1β in the joints of mice with CIA.

## Graphical abstract

**Figure:**
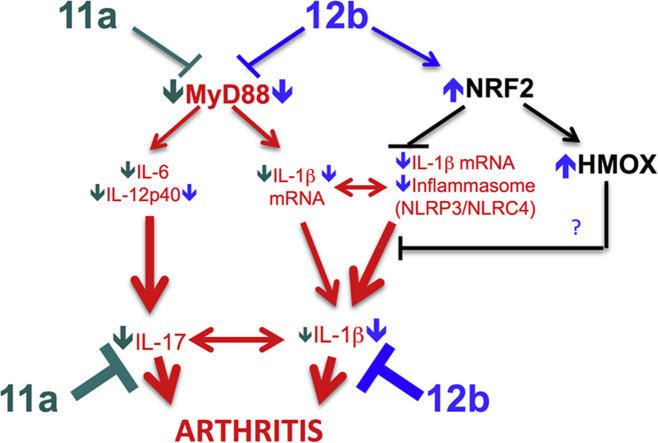

## 1 Introduction

Rheumatoid arthritis (RA) is one of the more common inflammatory diseases to affect Western societies with a prevalence rate of 1% [1]. Incidence increases with age, women are three to five times more likely than men to develop the condition and the disease is associated with decreased life span [2]. Although the advent of new biologic therapies has revolutionized the management of RA [3], not all patients are responsive and hence, the need to develop new drugs remains.

ES-62 is a glycoprotein secreted by the parasitic filarial nematode *Acanthocheilonema viteae* [4]. The molecule possesses anti-inflammatory properties and hence it has been tested in the collagen-induced arthritis (CIA) model of RA where it was found to protect against disease development [5–7]. ES-62 activity is dependent on an unusual post-translational attachment of phosphorylcholine (PC) [reviewed in Ref. [8]] and indeed PC attached to ovalbumin [6] or albumin [9] can mimic ES-62 in protecting against CIA. As a consequence of this, we hypothesized that it could be possible to synthesize novel small drug-like PC-based molecular analogues (SMAs) that mimic ES-62 activity: indeed, recently we produced a sulfone termed 11a that protects against CIA and appears to do so using the same mechanism of action as ES-62, namely inhibiting TLR-mediated pro-inflammatory cytokine responses, by partially downregulating MyD88 expression [9]. Here, we describe another novel sulfone, 12b, which also inhibits disease development in mice sensitized and challenged with collagen, but which contains additional previously unsuspected immunomodulatory properties. In particular, we have found 12b to modulate the expression of a number of genes associated with the inflammatory response, particularly those linked to IL-1β signalling, and which appear to be counter-regulated by activation of the transcription factor, NRF2 that plays a crucial cytoprotective role in the response to oxidative stress [10]. SMA-12b may thus be prototypic of a novel class of compounds of use in treating RA, in particular in those patients resistant to TNF-targeting biologics [11].

## 2 Materials and methods

### 2.1 Animals

Jirds and mice were bred and/or maintained in the Biological Services Units of the Universities of Glasgow and Strathclyde in accordance with Home Office UK Licenses PPL60/3119, PPL60/3580, PPL60/3791, PPL60/4300, PIL60/12183 and PIL60/12950 and the permission of the Ethics Review Board of both Universities. Collagen-induced arthritis (CIA) was induced in male DBA/1 mice (8–10 weeks old; Harlan Olac; Bicester, UK) by intradermal immunization with bovine type II collagen (CII, MD Biosciences) in complete Freund's adjuvant (FCA) and mice were treated with purified endotoxin-free 12b (1 μg/dose) or PBS subcutaneously on days −2, 0 and 21 and scored for development of arthritis as previously described [5–7]. In addition, the therapeutic effects of 12b were tested where following the onset of arthritis (mean score 3.25 ± 0.55), mice were treated subcutaneously with PBS or 12b (1 μg/dose) every 3 days (d0, d3 and d6). The Nrf2^−/−^ animals that were created by Itoh et al. [12] and provided kindly by Ken Itoh and Masayuki Yamamoto were backcrossed over six generations onto a C57BL/6 background.

### 2.2 Chemical synthesis and preparation of SMAs

Endotoxin-free ES-62 and SMAs-11a, -12b and -19o (for structures see Supplemental Fig. 1) were prepared as described previously, with the SMAs to ≥95% purity as shown by HPLC and ^1^H NIMR [7,9,13]. The SMAs were reconstituted at 100 mg/ml in cell culture-tested dimethyl sulfoxide (DMSO; Sigma–Aldrich) and then diluted in RPMI medium, or PBS when used *in vivo,* to 1 mg/ml and stored in microcentrifuge tubes at −20 °C. Compounds were filter-sterilised using a Millex-GP (0.22 μm; Millipore) filter unit prior to use in culture. All reagents and plasticware used were sterile and pyrogen free.

### 2.3 Analysis of pathology and IL-1β expression in the joint

Decalcified joint tissue section (7 μm) preparation, Haematoxylin and Eosin (H & E) and Trichrome staining and detection via immunofluorescence were performed as previously described [7,13]. The extent of synovitis, pannus formation, and destruction of bone and cartilage was determined using a graded scale: grade 0, no signs of inflammation; grade 1, mild inflammation with hyperplasia of the synovial lining and minor cartilage damage; grades 2 through 4, increasing degrees of inflammatory cell infiltrate and destruction of bone and cartilage. To detect IL-1β expression, sections were incubated with sodium citrate buffer (10 mM Sodium Citrate, 0.05% Tween 20, pH 6.0) for antigen retrieval and stained with a rabbit anti-mouse IL-1β antibody (Abcam; rabbit IgG isotype control) with DAPI as a counterstain, at 4 °C for 12 h, followed by detection using a biotinylated goat anti-rabbit IgG antibody and streptavidin–Alexa Fluor 647. Immunofluorescence images were obtained using an LSM 510 META confocal laser coupled to an Axiovert 200 microscope (Zeiss) and analysed by Zeiss LSM Image Browser software.

### 2.4 *Ex vivo* analysis

Draining lymph node (DLN) cells (10^6^/ml) were incubated ± 50 ng/ml PMA plus 500 ng/ml ionomycin for 1 h before addition of 10 μg/ml Brefeldin A (Sigma–Aldrich, UK) for a further 5 h at 37 °C with 5% CO_2_. Live cells were discriminated by the LIVE/DEAD fixable aqua dye (Invitrogen) and phenotypic markers were labelled using anti-CD4-PerCP, anti-CD8-FITC or anti-γδ-PE (BioLegend) antibodies before the cells were fixed and permeabilised using BioLegend protocols. Cells were then labelled using anti-IFNγ-Pacific Blue or anti-IL-17A-APC (BioLegend) antibodies for 30 min prior to flow cytometry and gated according to appropriate isotype controls as described previously [7]. IL-12p40 and IL-17 levels in serum or DLN, bmM and peritoneal exudate cell (PEC) supernatants were detected by ELISA using kits from BioLegend as described previously [7] whilst levels of IL-1β were determined by ELISA using kits from eBioscience according to the manufacturer's recommendations.

### 2.5 In vitro analysis of bone marrow-derived macrophages (bmMs)

Macrophages were prepared from bone marrow progenitor cells obtained from 6- to 8-wk-old male BALB/c mice and DBA/1 mice with CIA or C57BL/6, MyD88 and NRF2 knockout mice. Bone marrow progenitor cells were cultured for 7 days at 37°/5% CO_2_ in complete Dulbecco's modified Eagle's medium (DMEM; GIBCO) supplemented with 20% L929 cell culture supernatant (contains CSF-1), 10% heat-inactivated Fetal Calf Serum (HI FCS), 2 mM L-glutamine (GIBCO), 50 U/ml penicillin (GIBCO) and 50 μg/ml streptomycin (GIBCO) with fresh medium being added on day 4 [14]. The cells were analysed by flow cytometry, and were shown routinely to be ≥99% positive for CD11b and F4/80 markers.

BmMs were cultured in RPMI medium (PAA Laboratories) supplemented with 10% HI FCS, 2 mM L-glutamine, 50 U/ml penicillin and 50 μg/ml streptomycin (complete RPMI) in triplicate (2 × 10^5^ cells/well) in 96-well plates and were rested overnight prior to exposure to the indicated concentration of SMAs for 18 h. In some experiments, bmMs were then stimulated with either *Salmonella minnesota* lipopolysaccharide (100 or where indicated, 1000 ng/ml LPS; Sigma), BLP (10 ng/ml Pam3CSK4; Axxora Ltd) or CpG (0.01 μM; Source Bioscience Autogen) for 24 h and cell supernatants analysed for cytokine production by ELISA. ELISAs were performed according to the manufacturer's instructions, using paired antibodies from BD Bioscience Pharmingen for IL-12p40 and IL-6 and R&D systems for IL-1β.

### 2.6 TransAm (NFκB p65)

BmMs were cultured in 6 well plates (4 × 10^6^ cells/well) in complete RPMI medium. After 24 h, the medium was changed and the cells were pretreated with or without SMAs (5 μg/ml) for 18 h before being stimulated with 100 ng/ml LPS, 100 ng/ml BLP or 1 μM CpG for 1 h. Treatment with the SMAs alone did not activate NFκB p65 (results not shown). Activated NFκB p65 was measured in nuclear fractions (isolated using a Nuclear Extraction Kit; ActiveMotif) by the ELISA-based TransAM kit (ActiveMotif) according to the manufacturer's instructions.

### 2.7 Flow cytometric analysis of cell death

Cell death was determined by 7-Amino Actinomycin D (7-AAD; BD Pharmingen) staining of bmMs after stimulation with compounds 12b and 19o. bmMs (2 × 10^5^/well) were pretreated with SMAs (5 μg/ml) for 18 h prior to being stimulated with either 100 ng/ml *S. minnesota* LPS (Sigma), 100 ng/ml BLP (Pam3CSK4) or 0.01 μM CpG for 24 h. The ability of the compounds to spontaneously induce cell death was also tested. The cells were washed in PBS containing 1% FCS, then subsequently incubated with 5 μL of 7-AAD for 10 min on ice in the dark. Flow cytometry was conducted using a FACS Canto immunocytometry system (Becton Dickinson Pharmingen) and data were processed using FlowJo software (Tree Star Inc., OR USA).

### 2.8 Laser scanning cytometry (LSC)

BmMs (10^4^/well) from mice with CIA treated with either PBS or 12b (1 μg/ml) were incubated for 18 h in Lab-Tek chamber slides (Nunc) before being stimulated with LPS (100 ng/ml) for 15 min then fixed with 4% formaldehyde for 15 min [15]. Samples were quenched with 50 mM ammonium chloride (Fisher Scientific) for 10 min, washed, permeabilised with 0.1% Triton X-100 (Sigma) in PBS for 20 min and then washed and incubated for 20 min with PBS containing 1% BSA and 10% normal goat serum (Sigma). Cells were then incubated with anti-phospho-p65 (pp65; Cell Signalling) or the relevant isotype control rabbit IgG (Santa Cruz Biotech Inc) in PBS containing 1% BSA and 10% normal goat serum overnight at 4 °C. The cells were washed, and incubated with fluorescein-conjugated anti-rabbit IgG (Vector Laboratories) at 10 μg/ml in 1% BSA, 10% normal goat serum in PBS for 1 h in the dark. Cells were washed and stained with DAPI (Invitrogen) as a counterstain. Cells were washed again, and the slides mounted in Vectashield (Vector Laboratories) for analysis by LSC. LSC data were analysed using WinCyte software (CompuCyte). Using the relocation feature of the LSC, areas with the average representative fluorescence were relocated to and digital images of the stained cells were obtained using a Hammamatsu camera and Openlab software (Improvision) [15].

### 2.9 Cell lysates and Western blotting

BmMs (2 × 10^6^ cells/sample) were lysed by the addition of ice-cold, modified RIPA buffer (50 mM Tris, pH 7.4, 150 mM sodium chloride, 2% (v/v) NP-40, 0.25% (w/v) sodium deoxycholate, 1 mM EGTA, 1x Halt protease and phosphatase inhibitors [Pierce]) and solubilised on ice for 30 min. Protein (30 μg) samples were resolved on the XCell *SureLock* Mini-Cell kit with NuPAGE Novex high-performance pre-cast Bis-Tris gels and NuPAGE buffers and reagents (Invitrogen Life Technologies). Proteins were transferred to nitrocellulose (Amersham) or PVDF (Millipore, Watford, UK) and membranes were blocked by incubating for 1 h in 5% non-fat milk in TBS/Tween (0.5 M NaCl and 20 mM Tris pH7.5 with 0.1% (v/v) Tween-20) at RT. Membranes were incubated with primary antibody diluted in 5% BSA in TBS/Tween buffer overnight at 4 °C, washed with TBS/Tween and incubated with the appropriate horseradish peroxidase (HRP)-conjugated secondary antibody in 5% non-fat milk in TBS/Tween for 1 h at RT. Membranes were then washed with TBS/Tween and protein bands were visualised using the ECL detection system. Quantification of the bands was performed using ImageJ software (National Institute of Health, NIH).

### 2.10 Microarray

BmMs (2 × 10^6^ cells/well in 6-well plates) were incubated with medium, 12b, 19o (both 5 μg/ml) or ES-62 (2 μg/ml) for 4 h. Cells were harvested into RLT buffer and RNA prepared using the RNeasy Mini kit (Qiagen) and residual DNA cleared with DNaseI (Invitrogen), according to the manufacturers' protocols. Checking of RNA quality, cDNA preparation and microarray were performed at the Glasgow Polyomics Facility at the University of Glasgow using standard Affymetrix protocols. Triplicate biological replicates were hybridised to Affymetrix Mouse Gene 1.0 ST arrays representing over 28,000 genes. Bioinformatical data analysis was commissioned in the Bioinformatic Services Miltenyi Biotec GmbH (Bergisch-Gladbach, Germany). Briefly, raw microarray data were preprocessed using GC-RMA method and corrected for batch effect. The normalized log2 intensities values were centered to the median of all samples for each transcript cluster ID, i.e. the median was subtracted from each individual log2 expression value. Such ratio data in log2 space were used to create heat maps in which red shading indicates a stronger expression of the representative gene in comparison to the median of the total sample, and green represents a relative downregulation, respectively. Next, the different samples were compared to each other by a correlation analysis in order to get an impression of inter-sample similarity or variability. The obtained inter-experiment correlation coefficients based on the normalized log2 intensities were generated for all samples and displayed in clustered images. Positive correlation is indicated by shades of yellow (higher correlation = brighter color), while less well correlated samples are indicated by shades of black. In order to select differentially regulated transcripts between SMA-12b-stimulated and unstimulated samples the following selection criteria were applied: adjusted p value ≤0.1 (calculated by the method of Benjamini and Hochberg) and at least 1.5-fold expression difference. All differentially expressed genes were analysed through the use of IPA (Ingenuity^®^ Systems, www.ingenuity.com) software to detect up-stream regulators (transcription factors, TF) that may be responsible for the observed changes in the gene expression using experimentally observed relationships between TFs and genes. The IPA TF analytical tool determines a z-score that establishes whether gene-expression changes for known targets of each TF are consistent with what is reported as “activation” in the literature (z > 0, TF predicted as “activated”), or if the changes reflect inhibition as described in the literature (z < 0, TF predicted as “inhibited”). Z-scores greater than 2 or -2 are considered significant. Next, all of the significantly down-regulated TFs i.e. RelA, NFκB1 and HMGB1 were displayed as a network to graphically represent the molecular relationships between molecules. Molecules are represented as nodes, and the biological relationship between two nodes is represented as a line. All relationships are supported by at least one reference from the literature, from a textbook, or from canonical information stored in the Ingenuity Knowledge Base. The intensity of the node color indicates the degree of up- (red) or down- (green) regulation. Nodes are displayed using various shapes that represent the functional class of the gene product. For signalling pathways, an arrow pointing from node A to node B indicates that A causes B to be activated (e.g. by binding, phosphorylation, dephosphorylation, etc) and for ligands/receptors pathways: an arrow pointing from a ligand to a receptor signifies that the ligand binds the receptor and subsequently leads to activation of the receptor. This binding event does not necessarily directly activate the receptor; activation of the receptor could be caused by events secondary to the ligand/receptor-binding event. Solid lines indicate direct interactions whereas dotted lines, indirect interactions.

### 2.11 qRT-PCR

Total RNA was extracted using an RNeasy plus kit (Qiagen) and ≤ 1 μg of RNA was used to synthesize cDNA (Applied Biosystems). TaqMan^®^ RT-PCR was performed using the following TaqMan^®^ Gene Expression Assays: IL-1β (Mm01336189_m1), chemokine receptor 5 (CCR5: Mm01216171_m1), chemokine receptor 2 (CCR2: Mm00438270_m1), chemokine ligand 10 (CXCL10; Mm00445235_m1), complement component 5a receptor 1 (C5AR1; Mm00500292_s1), CD274 (PD-L1) (Mm00452054_m1), CD200 receptor 1 (CD200R1: Mm02605260_s1), NLRP3 (Mm00840904_ml), NLRC4 (Mm01233149_ml), glutamate-cysteine ligase, modifier subunit (GCLM; Mm00514996_ml), glutamate-cysteine ligase, catalytic subunit (GCLC: Mm00802655_ml) and haem oxygenase 1 (HMOX1; Mm00516005_ml), all from Applied Biosystems. Polymerase chain reactions were performed in triplicate in a StepOne sequence detector (Applied Biosystems). Data analysis was performed using the Applied Biosystems sequence detection software and samples were normalized to the reference reporter mouse glyceraldehyde 3-phosphate dehydrogenase (GAPDH; Mm99999915_g1) endogenous control.

### 2.12 Statistical analysis of data

Parametric data were analysed by the unpaired one-tailed Student's t test or by 1-way ANOVA. Normalised data were analysed by the Kruskal–Wallis test whilst the Mann–Whitney test was used for analysis of clinical CIA scores where *p < 0.05, **p < 0.01 and ***p < 0.001.

## 3 Results

### 3.1 SMA-12b protects against CIA

We have recently provided proof of concept that screening of PC-based compounds for their ability to suppress TLR2-, TLR4- and TLR9-mediated production of Th1/Th17-promoting cytokines (IL-12p40 and IL-6) by macrophages allows the selection of SMAs, such as the sulfone 11a, that mimic the ability of ES-62 to protect against CIA by suppressing pathogenic IFNγ and IL-17 production [9]. Although another sulfone, 12b, could also significantly reduce IL-12p40 secretion, it was not as effective with respect to IL-6 [9]. Nevertheless, it was found to be as effective as ES-62 [5–7,13] and 11a [9] in preventing the development of arthritis in the prophylactic CIA mouse model, as indicated by reduction in each of articular score (Fig. 1A), hind paw width (Fig. 1B) and disease incidence (Fig. 1C; score ≥2). Furthermore, importantly as with ES-62 [5] and SMA-11a (unpublished), when SMA-12b was administered therapeutically after the onset of arthritis; it protected against further disease development (Fig. 1D).

Consistent with the *in vitro* screening studies, whilst 12b- or PBS-treated mice undergoing CIA in the prophylactic model did not display altered numbers of leukocytes in the peritoneal exudate (PEC; Fig. 1E) or frequencies of macrophages within this population (Fig. 1F) when compared to healthy naïve mice, we observed significantly elevated levels of IL-12p40 in the peritoneal fluid of mice undergoing CIA that were reduced to the levels observed in naïve mice by *in vivo* exposure to 12b (Fig. 1G). Similarly, the low levels of IL-12p40 spontaneously secreted *ex vivo* by DLN cells were reduced to levels comparable to those produced by naive cells, in cultures derived from 12b-treated mice undergoing CIA (Fig. 1H). However, unlike ES-62 and 11a, SMA-12b did not suppress the number of total DLN cells or CD4^+^, CD8^+^ and γδ T cells (Fig. 1I and results not shown). Moreover, although there was a trend towards reduction in the number of IFNγ-producing DLN cells, specifically CD4^+^, CD8^+^ and γδ T cells following PMA/Ionomycin stimulation, this did not reach statistical significance (Fig. 1J and results not shown). Furthermore, 12b did not reduce the number of PMA/ionomycin-stimulated DLN or CD4^+^ or γδ T cells that were capable of producing IL-17 (Fig. 1K and results not shown) or lower the serum levels of IL-17 in mice undergoing CIA (Fig. 1L). This lack of modulation of IL-17/IFNγ responses *in vivo* was rather surprising as IL-12p40, as a component of both IL-12p70 and IL-23, is a therapeutic target (ustekinumab) in inflammatory autoimmune diseases [16,17] due to its ability to promote differentiation and/or maintenance of Th1 and Th17 cells. Nevertheless, these studies demonstrated that despite exhibiting some potential to suppress the cytokine milieu associated with Th17/Th1-driven pathogenesis in arthritis, both *in vitro* and *in vivo*, the protection afforded by 12b did not appear to depend on suppressing the Th17/Th1 phenotype associated with pathogenesis in CIA.

### 3.2 SMA-12b modulates inflammatory response gene expression in bmMs

To investigate the mechanism(s) underlying the protection against CIA afforded by SMA-12b, genome-wide microarray of macrophages was undertaken. Bioinformatical analysis revealed that whilst ES-62 and an SMA, 19o, that had been found not to modulate TLR-driven proinflammatory cytokine production in the *in vitro* screens [9], essentially did not alter the gene expression profile, treatment of macrophages with 12b for 4 h resulted in 364 genes being up-regulated and 496 genes being down-regulated in comparison to un-stimulated cells (a complete list of the affected genes and associated information provided by Ingenuity Pathway Analysis is shown in Supplementary Table 1). Indeed, tree structure analysis of sample clusters according to their degree of similarity shows that only 12b-stimulated cells clearly separated from the three other experimental conditions (Fig. 2A), with the 30 most down- and up-regulated genes shown (Fig. 2B). Furthermore, it was noted that when examining all of the data, a number of genes of possible relevance to RA were affected (Supplementary Table 2). For example, several associated with pro-inflammatory cytokine responses (e.g., IL-1β) and cell migration and recruitment, particularly of monocytes (e.g., NR4A1, CXCL10, CXCL3, CCR2, CX3CR1 and TREM) were down regulated and many of these were members of the top 30 downregulated genes. At the same time, some genes that play an inhibitory role in inflammation e.g., CD200R1 and CD274 (PD-L1) and have recently been identified as therapeutic targets in RA [18,19] were up-regulated. The data for a number of these key genes have been validated by qRT-PCR (Fig. 2C).

### 3.3 SMA-12b inhibits the secretion of IL-1β

IL-1β was found to be the most downregulated gene in bmMs exposed to SMA-12b (Supplementary Tables 1 and 2) and so we next attempted to obtain evidence linking this effect of the SMA to the release of IL-1β. First, we determined whether 12b inhibited LPS-mediated secretion of IL-1β by bmMs and this was found to be the case (Fig. 3A). We then investigated mice undergoing CIA and found that DLN cells from SMA-12b-treated mice displayed reduced ConA-stimulated IL-1β production relative to cells from mice undergoing CIA treated with PBS or from naïve mice (Fig. 3B). Finally, crucially it was found that staining of IL-1β production in the joint reveals that this is greatly inhibited in mice that show reduced disease development as a consequence of exposure to 12b (Fig. 3C&D).

### 3.4 SMA-12b is predicted to target transcription factors

To identify the transcription factors potentially responsible for the observed SMA-12b-mediated changes in macrophage gene expression, we used IPA for Transcription Factors (TFs). This software predicted, based on prior knowledge of expected effects between transcriptional regulators and their known target genes, the activation z-scores of 3 TFs - RelA (−2.996), NFκB1 (−2.223) and HMGB1 (−2.168) to be significantly inhibited by SMA 12b and 6 TFs, NFE2L2 (NRF2; 3.630), NKX2-3 (3.300), CBFB (2.630), TRIM24 (2.611), RXRA (2.213) and DACH1 (2.000) to be significantly activated. The TF with the lowest activation z-score and therefore the most inhibited by 12b was RelA, the p65 signalling element of the NFκB pathway. Consistent with this, we found that SMA-12b inhibits p65 NFκB activation in bmMs stimulated with each of TLR2 (BLP), TLR4 (LPS) and TLR9 (CpG) ligands *in vitro* (Fig. 4A). Moreover, exposure of mice undergoing CIA to 12b *in vivo* resulted in bmMs with reduced capacity for phosphorylation and consequent activation of p65 (pp65) in response to stimulation with LPS *ex vivo* (Fig. 4B). RelA-regulated genes with changes in their mRNA levels following exposure of cells to 12b that correlated with inhibition of RelA are shown in Fig. 4C and include IL-1β, as reported earlier the most repressed gene amongst all those tested in the microarray (Supplementary Table 1). As mentioned above, IPA also projected inhibition of activity of another member of the NFκB family, namely NFκB1 (Fig. 4C), the 105 kD protein that is processed to produce the p50 TF and consistent with this, 12b down-regulated transcript levels of a number of NFκB1-dependent genes (Fig. 4C). Many genes whose expression was changed by SMA-12b and whose direction of change supported inhibition of RelA were present in this group of genes confirming that these two TFs act in concert to regulate gene expression levels and are both 12b targets. Collectively, these data suggest that 12b acts to suppress the hyperactive NF-κB (p65 and p50) signalling that promotes recruitment of inflammatory cells and generation of pro-inflammatory mediators such as IL-1β in RA joints [20].

To address the mechanisms responsible for suppressing NF-κB signalling, we turned to the TFs that were predicted by the IPA to be activated by SMA-12b (Fig. 4D) and, on the basis of the changes in the expression profile of their target genes, NFE2L2 (NRF2) was ascribed the highest activation score. Interestingly, therefore, given the convergence of hypoxia, reactive oxygen species (ROS) and the inflammasome in promoting the inflammation and angiogenesis that leads to joint damage in RA [21–25], a large group of genes, involved in protection against oxidative stress and controlled by this TF were up-regulated by SMA-12b (Supplementary Tables 1 and 2). For example, 12b drove increased expression of genes taking part in synthesis, regeneration and utilization of glutathione such as GCLM, GCLC, SLC7A11, GSTA3 and GSR (Fig. 4D). In addition, SMA-12b also increased mRNA levels of TXNRD1 and PRDX1, which are engaged in thioredoxin production, regeneration and utilization, as well as NQO1 and HMOX1 that play a role in quinone detoxification and iron sequestration, respectively.

That NF-κB-associated TFs and NRF2 were inversely targeted was particularly interesting as there is evidence in the literature that these elements counter-regulate [26,27] and also that NRF2 protects against joint damage in the antibody-induced arthritis (AIA) model of RA by limiting oxidative stress-induced cartilage destruction [28]. By contrast, crosstalk between NF-κB and Hypoxia-Inducible Factors (HIFs) has been shown to be arthritogenic [21]. It was therefore decided to further explore, including a comparison with SMA-11a, whether SMA-12b inversely targeted key NF-κB- (IL-1β, inflammasome genes) and NRF2- (HMOX1, GCLC and GCLM) dependent genes that may play roles in counter-regulating inflammation in RA.

### 3.5 SMA-12b modulates transcript levels of IL-1β/inflammasome and NRF2-controlled genes

Inflammasomes are closely associated with IL-1β as they form molecular platforms that drive the proteolytic cleavage by caspase-1, which results in release of bioactive IL-1β. Indeed, ROS-dependent NF-κB signalling via the NLRP3 inflammasome has been implicated in IL-1β-mediated pathogenesis in RA [22,23]. It was therefore interesting that in addition to IL-1β being the gene most repressed by 12b, the SMA down-regulated, albeit not as profoundly, levels of genes encoding several inflammasome molecules, including NLRP3 (−1.51 vs unstimulated) and NLRC4 (−1.69 vs unstimulated) (Supplementary Tables 1 and 2). We therefore further explored the modulation of IL-1β and these inflammasome genes in macrophages by SMA-12b and also, for comparison, the effects of 11a (both at 5 μg/ml; Fig. 5A–C). This confirmed that 12b downregulated steady-state expression of IL-1β and NLRC4 mRNA within 4 h but this was not the case for 11a. Moreover, whilst both SMAs downregulated NLRP3 expression within 4 h, significant suppression of NLRP3 mRNA was observed at 2 h with 12b, but not with 11a. We next investigated the effects of 11a and 12b on the mRNA levels of IL-1β and the inflammasome genes after simultaneous exposure of the cells to a pro-inflammatory stimulus, in this case, LPS (Fig. 5D–F). Under these pro-inflammatory conditions, 12b, but not 11a, also suppressed LPS-mediated upregulation of IL-1β and NLRP3. Although, 12b did not significantly inhibit the transient LPS-mediated upregulation of NLRC4, levels of this inflammasome component were reduced below basal levels within 4 h under all LPS-stimulated conditions tested. These differential effects may reflect that, for example, unlike NLRP3 that requires additional pro-inflammatory signals including bacterial TLR ligands for full induction, NLRC4 exhibits high levels of expression under steady-state conditions, and hence exposure to LPS may induce confounding effects in this case [24].

Likewise, as the IPA analysis revealed that SMA-12b increased levels of many antioxidant genes controlled by the transcription factor NRF2 (Fig. 6A), we assessed whether 12b and 11a (both at 5 μg/ml) differentially modulated the expression levels of HMOX1 (the most up-regulated gene from the antioxidant family) and two genes, GCLC and GCLM that are crucially involved in biosynthesis of glutathione, under steady-state and pro-inflammatory (LPS-TLR4 signalling) conditions. Consistent with the microarray data, 12b, but not 11a, was able to strongly upregulate expression of all 3 genes between 2 and 4 h (Fig. 6B–D). By contrast, LPS acted to downregulate HMOX1, GCLC and GCLM expression: however, this was prevented by 12b and indeed, even in the presence of LPS, this SMA, but not 11a, was able to induce their expression (Fig. 6E–G).

### 3.6 SMA-12b suppresses IL-1β and inflammasome genes via NRF2

NRF2 and NF-κB have been reported to counter-regulate gene induction and consistent with this, 12b downregulated NF-κB-regulated IL-1β and inflammasome genes whilst up-regulating expression of NRF2-controlled anti-oxidant genes (Figs. 5 and 6). We therefore hypothesised that deficiency in NRF2 could inhibit the ability of 12b to dampen-down genes classically known to depend on NFκB e.g. IL-1β. As predicted, while 12b significantly down-regulated the levels of IL-1β mRNA in a dose-dependent manner in bmM from WT mice, this was not apparent in NRF2 KO macrophages (Fig. 7A). Similarly, the 12b-induced down-regulation in the levels of NLRP3 and NLRC4 (Fig. 7B and C) was shown to be NRF2-dependent, as there was no significant differences in the mRNA levels of these genes between control and 12b-treated bmM from NRF2 KO mice, although it should be noted that NRF2 deficiency itself appeared to impact on the steady-state levels of NLRP3 and particularly, perhaps mimicking the effects of LPS, NLRC4 expression. NRF2-deficiency was validated by the suppression of 12b-mediated induction of GCLC observed in WT bmM (Fig. 7D). Of note, the counter-regulation of inflammasome and anti-oxidant genes that we are witnessing appears to be associated with SMA-12b protection against CIA as analysis showed increased levels of mRNA for GCLC (129%) and HMOX (121%) yet reduced levels of NLRP3 (74%) in splenocytes from 12b-treated relative to PBS-treated mice with CIA.

We next investigated the ability of 12b to suppress LPS-production of IL-1β in NRF2-deficient bmM and found it to be lost (Fig. 8A). By contrast, 12b-mediated inhibition of LPS-stimulated IL-12p40 or IL-6 production remained intact despite NRF2 deficiency (Fig. 8A & results not shown). This latter result presumably reflects that 12b, similarly to the parent molecule ES-62 and SMA-11a [9], downregulates steady-state levels of MyD88 expression in bmM (Fig. 8B; for 12b, 67.68 ± 8.34% of medium alone levels where n = 3 and p < 0.05). By contrast, we have recently reported that LPS upregulates MyD88 expression in bmMs [9], and in two further independent experiments we have shown that pre-exposure to 12b also resulted in downregulation of MyD88 expression in LPS-treated bmM (Fig. 8C and D). In addition to being essential for TLR4-mediated IL-12p40 production by bmMs [29], MyD88 is required for LPS-induction of IL-1β as well as for the NF-κB-mediated induction of NLRP3 and IL-1β by TLRs important in CIA/RA, such as TLR2 [30–32] and IL-1R [33,34] that do not couple via the TRIF, MyD88-independent pathway [35]: thus, we investigated whether 12b-mediated inhibition of IL-1β production was also associated with downregulation of MyD88 signalling. This revealed that although steady-state levels of IL-1β mRNA (Fig. 8E) are partially reduced by MyD88 deficiency, exposure to 12b resulted in a further reduction in levels. Steady-state levels of NLRP3, NLRC4, GCLC and HMOX1 expression and their modulation by 12b were predominantly independent of MyD88 expression (Fig. 8E and results not shown). Thus with respect to IL-1β, these data suggest that SMA 12b acts to inhibit pathogenic production of this cytokine by a dual-pronged mechanism, involving downregulation of MyD88 in addition to upregulation of NRF2 activation and converging at the level of the inflammasome (Fig. 8F).

## 4 Discussion

ES-62 is an immunomodulatory molecule secreted by the filarial nematode, *A. viteae* that exerts anti-inflammatory effects on both the innate and adaptive arms of the immune response to promote parasite survival and, as a consequence, exhibits therapeutic potential in a number of autoimmune and allergic inflammatory disorders [36]. However, due to being a large and potentially immunogenic protein, ES-62 is in reality not suitable for development as a therapy. Nevertheless, its anti-inflammatory activity is due to post-translational decoration with PC, allowing us to recently construct a library of drug-like compounds based around this active PC moiety as a potential starting point in the development of novel anti-inflammatory drugs as therapies in autoimmune inflammation.

The sulfone SMA-12b was selected for screening for anti-inflammatory actions in the mouse CIA model on the basis that its ability to inhibit PAMP-induced IL-12p40 and to a lesser degree IL-6 production should dampen down subsequent Th1/Th17 polarisation [9], a phenotype associated with pathology in this model [7,37]. This strategy has recently provided proof of concept with respect to the protective actions of the related SMA, 11a [9]. Like ES-62 and 11a, SMA-12b was found to afford protection against CIA in both prophylactic and therapeutic studies but detailed analysis of the former revealed that even allowing for 12b's reduced effectiveness at lowering IL-6 responses, such protection clearly did not appear to reflect the significant suppression of Th1/Th17 responses that had been noted with 11a.

As an approach to understanding how SMA-12b might be protecting against CIA in the light of its limited effects on Th1/Th17 responses, we turned to microarray analysis employing bmMs. This indicated that 12b was able to suppress IL-1β and associated inflammasome gene expression and also production of IL-1β protein. Moreover, analysis of the joints of CIA mice successfully treated with SMA-12b indicated a reduction in the level of IL-1β-expressing cells in the synovium. This represents a key finding given the importance of this cytokine in induction of pathology in both CIA [38] and RA [39] as evidenced by the effectiveness of IL-1-targeting biologics such as anakinra in suppressing the infiltration of inflammatory cells and joint damage in RA patients [33]. Subsequent IPA for Transcription factors (TF) helped provide an explanation for the effect on IL-1β by revealing that NF-κB signalling, which can promote both IL-1β production and effector responses and has been implicated in joint pathogenesis in CIA [20], was likely to be inhibited by SMA-12b and this was confirmed by functional analysis, both *in vitro* and *ex vivo*. At the same time, IPA indicated activation of NRF2; a key component of the response to oxidative stress [40,41]. This result was consistent with the observation that the two TFs are known to counter-regulate each other [26,27,42] and indeed NRF2^−/−^ mice were subsequently employed to show the importance of this TF to the inhibitory effects on production of the NF-κB target, IL-1β.

A key question is how SMA-12b is able to promote NRF2 activation. Like 12b, SMA-11a can cause inhibition of NF-κB [9] but it does not appear to activate NRF2 (Fig. 6). The major difference between 11a and 12b is that the latter is a quaternary ammonium salt as opposed to a tertiary amine, a structural difference that would be expected to have a substantial influence both on binding to receptors (through differences in hydrogen bonding ability and steric bulk) and on access to cells and cellular compartments (12b is permanently positively charged). The 4-substituent on the benzene ring (bromo in 11a and methyl in 12b) is also significantly different, particularly in terms of size, and might also influence receptor binding. However the most plausible explanation for the difference in effect on NRF2 is that 12b but not 11a is likely to be converted by β-elimination within the cell to a vinyl sulfone [43], a structure recently shown to cause activation of NRF2 [44]. The 12b-derived vinyl sulfone, as an electrophile, could in theory interact with thiol-groups on cysteine residues of the NRF2 repressor protein, Keap-1, and based on recent ideas reported in the literature [45] this could cause a conformational change that would allow release of NRF2, and translocation to the nucleus to drive expression of target genes such as HMOX1 which, like NRF2 itself [28], has been shown to be protective against inflammatory arthritis. Indeed, like 12b, HMOX1 appears to achieve protection in the CIA model by reducing each of NF-κB activation, production of IL-1β by synovial cells, and synovial fibroblast hyperplasia (pannus formation) [46]. Further support for 12b mediating activation of NRF2 by interacting with Keap1 is provided by the recent report of a crystal structure of the Btb domain of Keap1 with a triterpenoid antagonist bound through a sulfide link at residue C151 (Protein Data Bank 4cxt) [47]. Thus, future work designed to fully optimize the structure of SMA-12b in the drive towards the clinic will have particular focus on the role of vinyl sulfone conversion.

In addition to inducing NRF2-dependent inhibition of bioactive IL-1β production, 12b (like ES-62 and 11a but not 19o [9]) downregulates MyD88 expression: as this key TLR/IL-1R signal transducer is also critical for IL-1R (and for DAMP/TLR) coupling to the inflammasome [48], such downregulation amplifies suppression of IL-1β and associated transduction of its pathogenic effector functions (Fig. 8F). Moreover, by inducing TLR/IL-1R-hyporesponsiveness, MyD88 downregulation not only also provides a molecular mechanism for the observed 12b-mediated NRF2-independent inhibition of TLR-mediated IL-6 and IL-12 production but potentially impacts on IL-1R/TLR driven-MyD88-ARNO-Arf6 signalling that plays a key role in vascular leakage and consequent induction of inflammatory arthritis and joint damage [49–51].

Although 12b does not suppress IFNγ responses in CIA to the extent that they are inhibited by ES-62 and 11a, its ability to suppress expression of downstream effectors of IFNγ signalling such as IRFs (as evidenced by microarray analysis) may reflect MyD88 downregulation as this adaptor molecule appears to play a critical role in transducing effector immune responses in IFNγ-activated macrophages [52]. Similarly, MyD88-signalling in Meningococcal sepsis has been associated with high serum levels of C5a [53], a pro-inflammatory mediator that enhances TLR4/MyD88-mediated IL-17F production by macrophages [54] and is pathogenic in arthritis [55–57] due to its promotion of proinflammatory cell migration [58] and osteoclastogenesis, particularly in synergism with IL-1β [59]. Moreover, as osteoclasts, the cells responsible for bone resorption in chronically inflamed joints, can arise from the same progenitors as macrophages, it is intriguing in the context of RA that microarray analysis suggests that 12b can downregulate the transcription factor NFAT-C1, which has been proposed to be a master regulator of the osteoclast transcriptome [60] and can be induced in a TLR2/MyD88-dependent manner [61]. Thus, given the important role of spontaneous TLR2 signalling in synovial inflammation in RA [30] and reports that whilst TLR4 can preferentially couple to IL-6/IL-17 signalling, TLR2 signalling primarily results in IL-1β production in mice exposed to *Mycoplasma arthritidis* mitogen, a superantigen that induces inflammation resulting in arthritis, skin necrosis and shock [62], our microarray findings that TLR2, IL-1β and C5aR all appear to be targets of 12b in macrophages may further explain the differential protective effects of 12b and 11a in CIA.

Finally, further analysis of the microarray data reveals that the expression of a large number of genes apparently not related to inflammation and immunity are also modulated by SMA-12b. Amongst the 30 most down-regulated genes are: (i) Nr4al (NUR77; 7.75-fold decrease), a member of the steroid-thyroid hormone-retinoid receptor superfamily that acts as a nuclear transcription factor/orphan nuclear receptor and is currently being explored with respect to roles in cancer [63] and insulin resistance leading to type-2 diabetes [64]; (ii) Gpr65 (5.59-fold decrease), which acts as a receptor for psychosine [65]; (iii) x99383 (4.34-fold decrease), an RNA-specific editase of glutamate receptors [66]; (iv) Nuak1 (3.4-fold decrease), a serine/threonine protein kinase involved in a number of different biological processes relating to cell adhesion, senescence and proliferation [67] and (v) Ccrn4l (3.17-fold decrease), which intriguingly has a suggested role as a circadian clock effector (Nocturnin) [68] that promotes obesity [68,69]. Amongst the 30 most-upregulated genes are: (i) Ednrb (14.37-fold increase), the endothelin receptor type B [70]; (ii) Slc16a9 (12.9-fold increase), solute carrier 16, member 9, a monocarboxylic acid transporter [71]; (iii) RragD (6.42-fold increase), a Rag-like GTPase [72]; (iv) Ppap2b (6.09-fold increase), a plasma membrane-located phosphatidic acid phosphatase [73] and (v) Ext1 (5.94-fold increase), an ER-located glycosyltransferase involved in heparin sulfate biosynthesis [74]. Clearly, genes covering a wide range of functions (or possible functions) are being targeted and it is uncertain whether changes in expression of any of these genes or other non-immunity/inflammation genes affected by SMA-12b contribute to its protective effects against CIA. However, given the recent focus on how insulin resistance and consequent obesity [75,76] and circadian rhythms [77] impact on autoimmune inflammatory diseases such as RA, and that NRF2-dependent pathways act to protect against the dysfunction of metabolic pathways and biological clocks that exacerbates inflammatory diseases [78–80], it would be of interest in the future to proceed to determine whether experimentally knocking out/down or increasing the expression of such individual genes most affected by 12b offers any protection against disease in mouse models of RA and other inflammatory disorders.

## 5 Conclusion

In spite of initial therapeutic success, IL-1-targeting biologics were superseded by TNF-blockers in the treatment of RA, although lately there has been a resurgence of interest in such reagents [33]. This reflects recent proposals that IL-1 and IL-6 rather than TNF may be critical in the transition from acute to chronic disease [81] and, perhaps consistent with this, that patients refractory to TNF therapy respond better to IL-1-modulation rather than alternative TNF treatments [11]. This raises the possibility of using SMAs, such as 11a and 12b with complementary inflammatory targets in a stratified/personalized manner, taking into account both the differential kinetics of particular cytokines depending on the stage of the disease and also their site of action (such as the joint). For example, whilst IL-17 is secreted in high levels during the initiation phase of arthritis, this production is much reduced at the chronic stage [82]. Perhaps particularly pertinent to this suggestion, recent data suggest that certain synovial phenotypes are associated with responsiveness to biologic therapies: thus, good responses to anti-TNFα correlated with an IL-1-associated myeloid synovial signature whereas lymphoid synovial phenotypes, reflective of IL-17-driven pathogenesis, were less responsive to TNF blocking [83]. At the same time, targeting of C5a/C5aR [55–57] and TLR2 [32] has shown promise in experimental models of arthritis and thus the increasing evidence of crosstalk between complement, TLR and IL-1R signalling in inflammatory pathologies such as RA, for example by promoting cellular migration and osteoclastogenesis [59] makes SMAs that potentially target all three convergent pathways an attractive proposition for development of novel treatments for RA. Encouragingly, therefore, in consideration of the route to the clinic, our early preliminary data suggest that ES-62 and 11a but not 12b can inhibit LPS-stimulated IL-6 production from PBMC from RA patients and also that (at least) ES-62 can similarly reduce IL-17 production. Moreover, SMA-12b modulates expression of a number of genes in human mast cells similarly to that witnessed with mouse macrophages (unpublished data). Furthermore, and in line with the observed lack of toxicity of 11a and 12b *in vitro*, preliminary ADMET data show no hERG liability or cytochrome p450 enzyme inhibition associated with these compounds (Supplementary Fig. 1 and data not shown). Finally, the recent dramatic success of IL-1-blocking therapies in a wide range of autoinflammatory syndromes indicates that there may be more widespread application of such complementary drugs in a diverse range of these previously intractable and debilitating conditions, as well as to more common IL-1-mediated disorders such as post-infarction heart failure [33,84,85].

## Funding

This work was funded by grants from the Wellcome Trust (086852), the Bioscience and Biotechnology Research Council (BBSRC: E013929), MRC (Confidence in Concept) and Arthritis Research UK (18413). DTR and MC were awarded studentships funded by the Wellcome Trust and FL by the BBSRC respectively.

## Author contributions

WH, MMH, CJS and JR conceived the study. JR, MAP, LA, DTR, MC and FL performed the experiments. JH and AK synthesised the ES-62 small molecule analogues and LA and FL manufactured ES-62. PJM and MLA prepared the NRF2^−/−^ mice. WH, MMH and JR wrote the paper. WH, MMH, JR, LA, CJS and MAP evaluated and interpretated the emerging data. All authors critiqued and proof read the manuscript and approved the final version.

## Non-author contributions

Padraic Fallon of Trinity College, Dublin and Susanne Hartmann of Freie Universitat, Berlin, kindly provided tissues from MyD88^−/−^ mice.

## Conflicts on interest

The authors have no conflicts of interest.

## Figures and Tables

**Figure:**
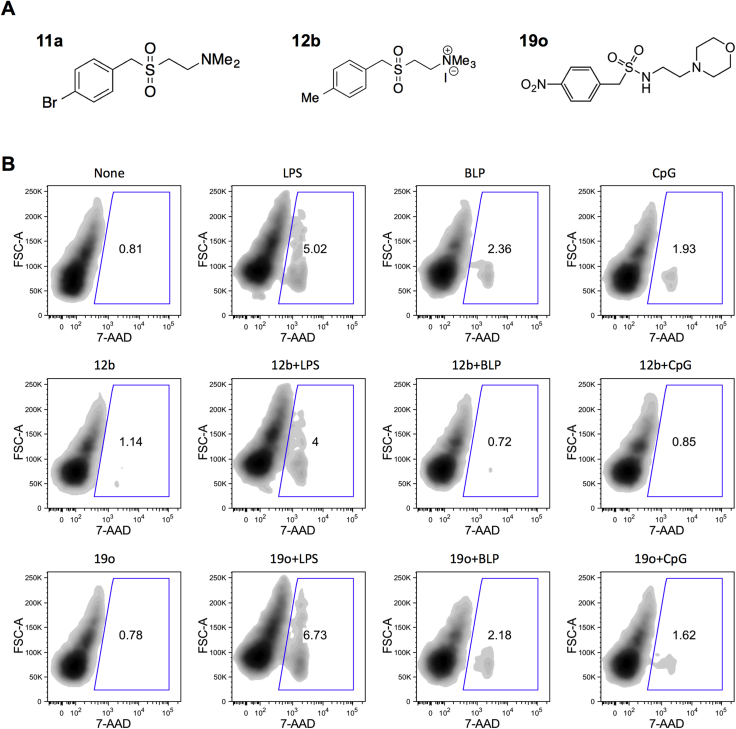


**Fig. 1:**
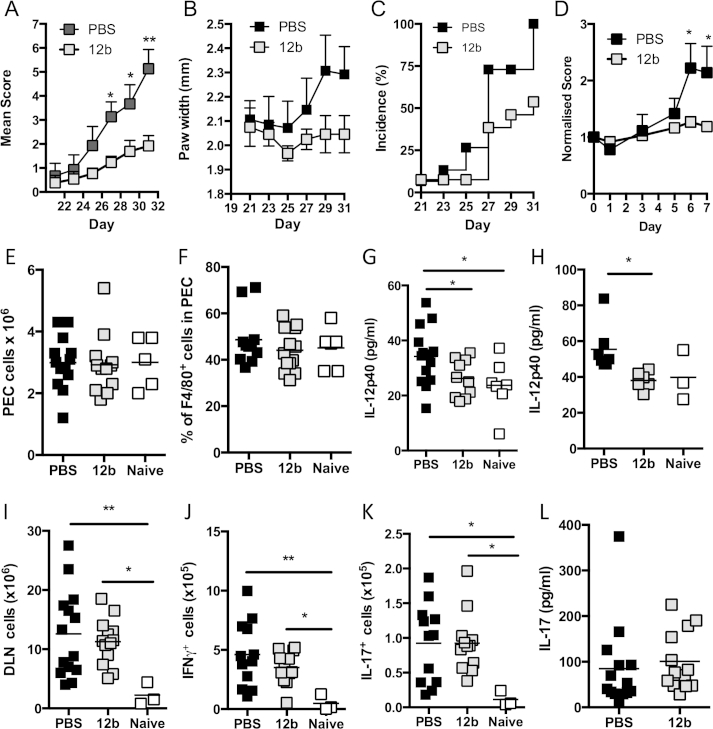
*SMA-12b protects against CIA in an IL-17-independent manner*. Development of CIA by (A) Mean Arthritis Score (PBS, n = 15; 12b, n = 13; data pooled from 2 independent experiments) and (B) hind paw width (PBS, n = 7; 12b, n = 6; data from single experiment), where results are expressed as mean scores ± SEM for PBS or 12b-treatment groups of collagen-exposed mice. Incidence (C), indicated by % of mice developing a severity score ≥2 is shown (PBS, n = 15; 12b, n = 13). (D) Following development of arthritis (d0), mice were treated every 3 days with PBS or 12b (both n = 6) and score for each mouse normalised to that at day 0. Peritoneal cavity cells were counted (E) (PBS, n = 11; 12b, n = 12; naive, n = 6) and frequency of F4/80^+^ cells determined by FACS (F) (PBS, n = 13; 12b; n = 12, naive, n = 5). Peritoneal fluid was concentrated and IL-12p40 measured by ELISA (G) (PBS, n = 14; 12b, n = 10; naive, n = 7). For E-G, each value represents data from individual mice with data pooled from two independent experiments. (H) IL-12p40 spontaneously released by DLN cells from mice undergoing CIA and treated as indicated are shown where data are presented as the mean values of individual mice from one experiment (naïve, n = 3; PBS, n = 7; 12b, n = 6). (I) Total numbers of DLN cells of individual mice from the naïve (n = 3), PBS-treated (n = 14) and 12b-treated (n = 13) groups are shown. (J) The number of IFNγ-expressing DLN cells and (K), IL-17-expressing DLN cells following stimulation with PMA/ionomycin from individual mice is shown (naïve, n = 3; PBS, n = 12; 12b, n = 12). (L) Serum IL-17 levels are plotted as mean values of triplicate IL-17 analyses from individual mice (PBS, n = 14; 12b, n = 13). *p < 0.05; **p < 0.01 and ***p < 0.001.

**Fig. 2:**
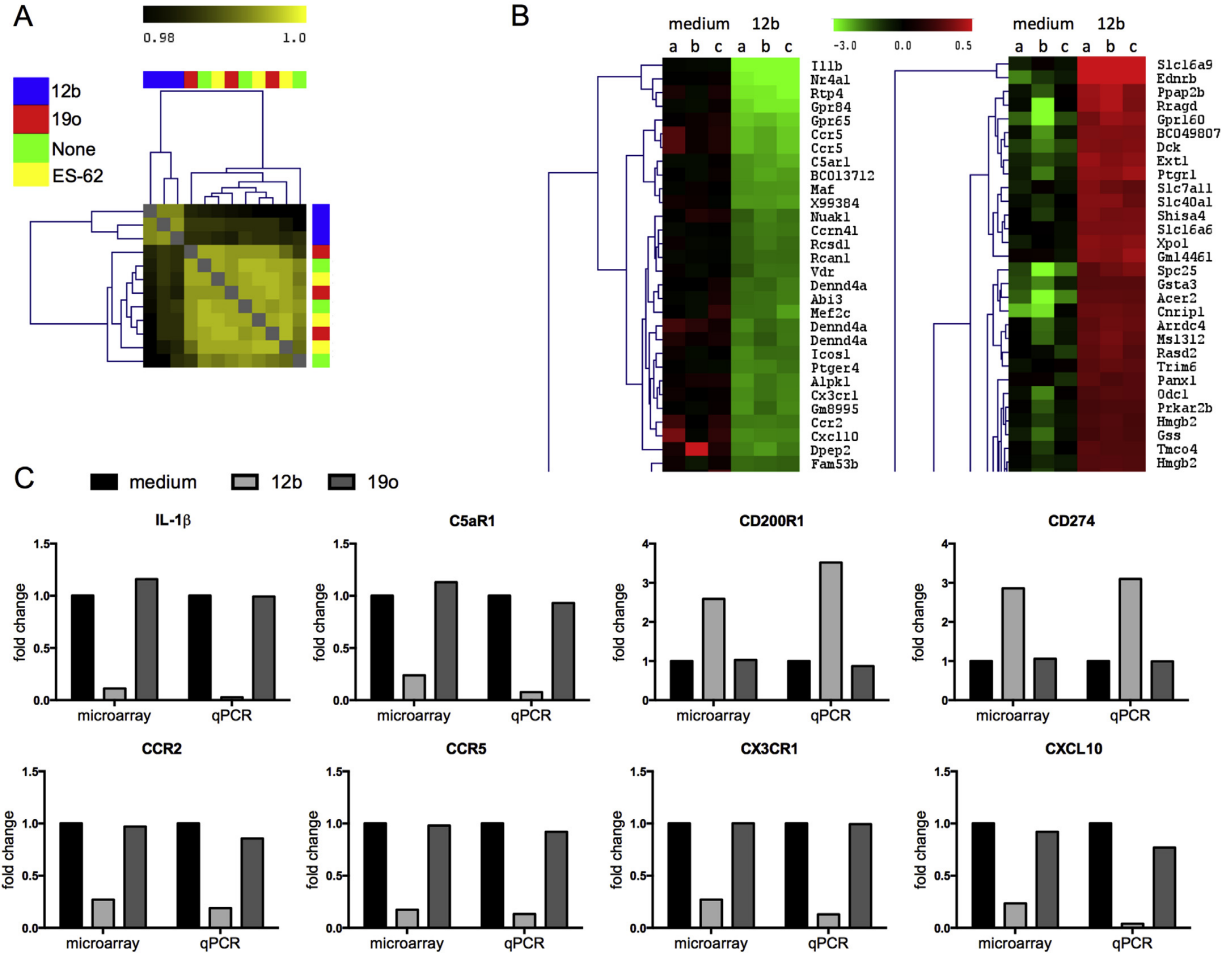
*SMA-12b modulates gene expression in bmMs*. (A) The tree structure indicates clusters of samples according to their degree of similarity. Positive correlation is indicated by shades of yellow (higher correlation = brighter colour), while less well correlated samples are indicated by shades of black. The colour bar on top of the tree indicates the treatment group assignment: green, none; blue, 12b; red, 19o and yellow, ES-62, of samples from 3 independent experiments. (B) Excerpt of a clustered heat map (Euclidean distance, complete linkage) showing 30 most down-regulated and 30 most up-regulated reporters in triplicate samples from 3 independent experiments (a–c) of 12b-treated macrophages compared to un-treated cells (medium). (C) Microarray data were validated for selected target genes by qRT-PCR where the levels of the gene of interest were normalized to the level of GAPDH and expressed as a fold change for 12b (and 19o) with respect to the medium control. Data shown are means from three biological replicates.

**Fig. 3:**
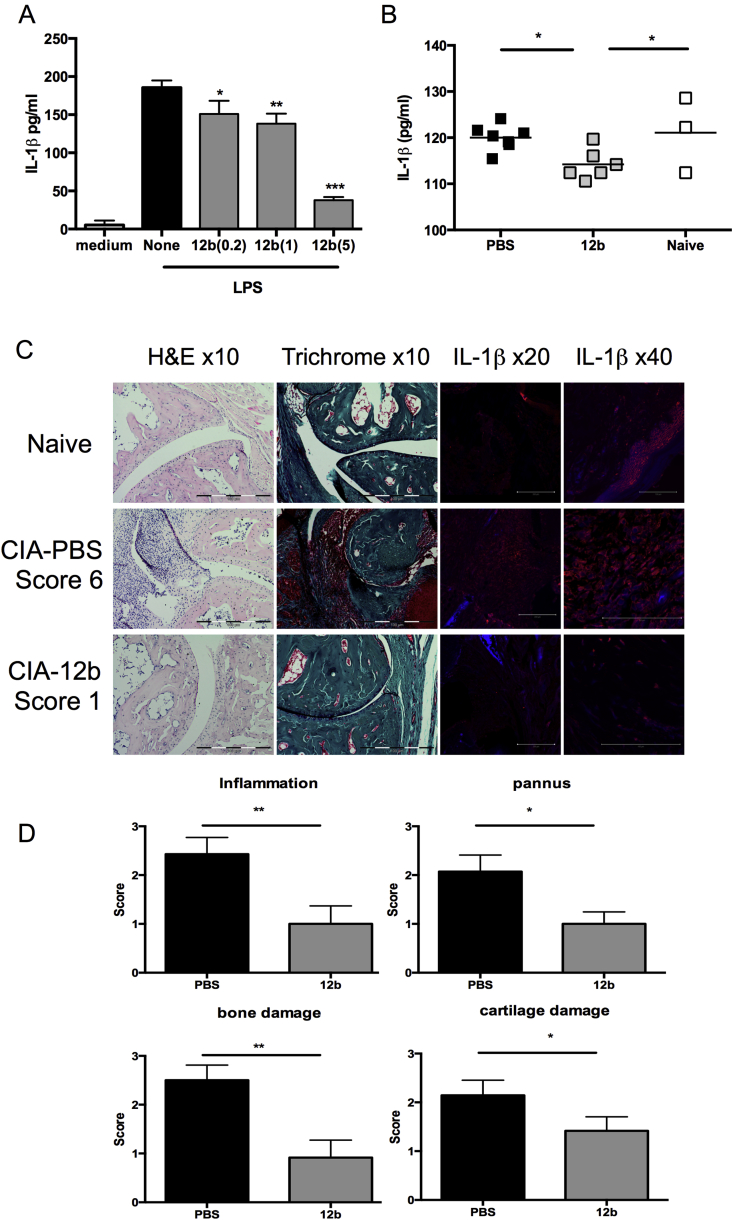
*SMA-12b inhibits IL-1β production*. (A) BmMs from BALB/c mice pre-treated with the indicated concentration (μg/ml) of 12b or medium alone were then stimulated with LPS (1 μg/ml) and IL-1β release determined by ELISA. Data presented are the mean values ± SD for replicate cultures (medium, n = 7; none, n = 6; 12b(0.2), n = 4; 12b(1), n = 8 and 12b(5), n = 8) from 3 individual mice (except for 12b(0.2) cultures where data were only obtained for 2 mice). (B) DLN cells from naive DBA/1 mice or DBA/1 mice undergoing CIA treated with either PBS or 12b were stimulated with ConA (1 μg/ml) and levels of released IL-1β determined. Data shown are the mean values for individual mice (naive, n = 3; PBS, n = 7; 12b, n = 6). (C) Joint sections from individual mice representative of each treatment group were assessed for histopathology (10× magnification; H & E and Trichrome staining; scale bars 100 μm, no zoom) and also IL-1β expression by immunofluorescence (magnification 20×: scale bars 200 μm and scan zoom 0.7; 40×: scale bars 100 μm and scan zoom 0.7 for naive and 1.5 for PBS and 12b). Isotype controls were negative and the strong IL-1β staining in the naïve (40×) image reflects high production of IL-1β (31kD) by keratinocytes in the portion of skin in the section included as an additional control for validation of the antibody specificity. Parameters of histopathology were scored (D) with the data presented as mean values from individual mice ± SEM (n = 14 for PBS-; n = 12 for 12b-treatment groups). *p < 0.05; **p < 0.01 and ***p < 0.001.

**Fig. 4:**
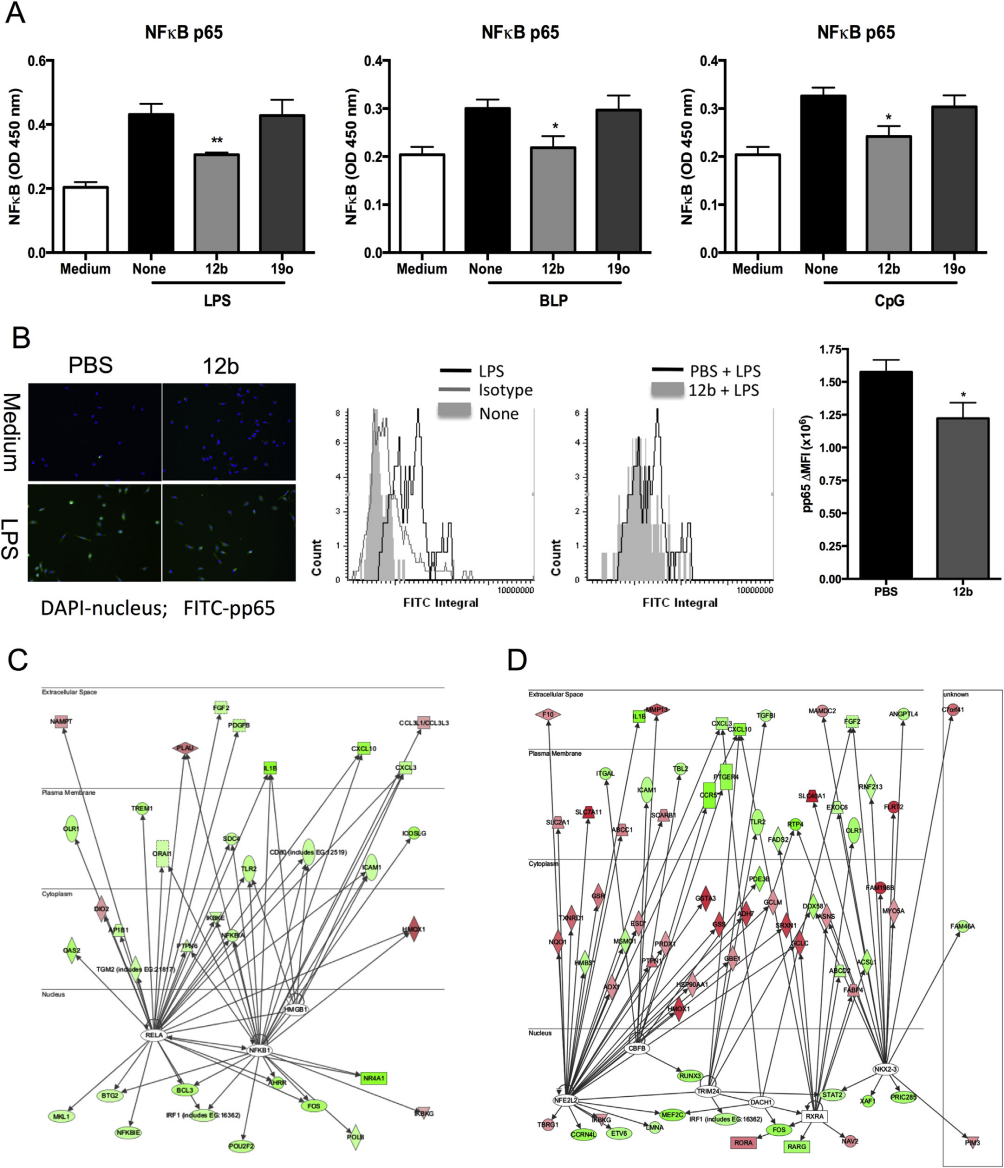
*SMA-12b inhibits TLR-induced NFκB activation in macrophages*. (A) BmMs were pre-incubated for 18 h with SMAs (5 μg/ml) and then stimulated with 100 ng/ml LPS, 10 ng/ml BLP or 1 μM CpG for 1 h. “None” represents no SMA pre-treatment and “medium”, no PAMP treatment. p65 activation was measured by the TransAM assay and data presented are mean values ± SEM from three independent experiments, *p < 0.05; **p < 0.01 (B) BmM from CIA mice treated with PBS or 12b were incubated with medium or LPS (100 ng/ml) and assessed for expression of pp65 (green) against a DAPI (blue) nuclear counterstain. Histograms are presented showing gating of pp65^+^ cells relative to the isotype control; the increase in pp65 expression by LPS-treated cells relative to that in unstimulated bmMs and the levels of pp65 expression in LPS-stimulated bmMs derived from CIA mice exposed to PBS or 12b (>200 individual cells/group) were analysed. Data are then presented as the mean values ± SD, n = 3, of the difference in mean fluorescence integral (ΔMFI) of LPS-stimulated cells relative to their medium controls. IPA prediction of 12b-mediated down-regulation of RelA/NFkB1 (C) and up-regulation of NRF2 NFE2L2; (D) signalling based on expression of their target genes by microarray analysis is shown. Genes down-regulated or up-regulated by 12b are shown in green and red, respectively.

**Fig. 5:**
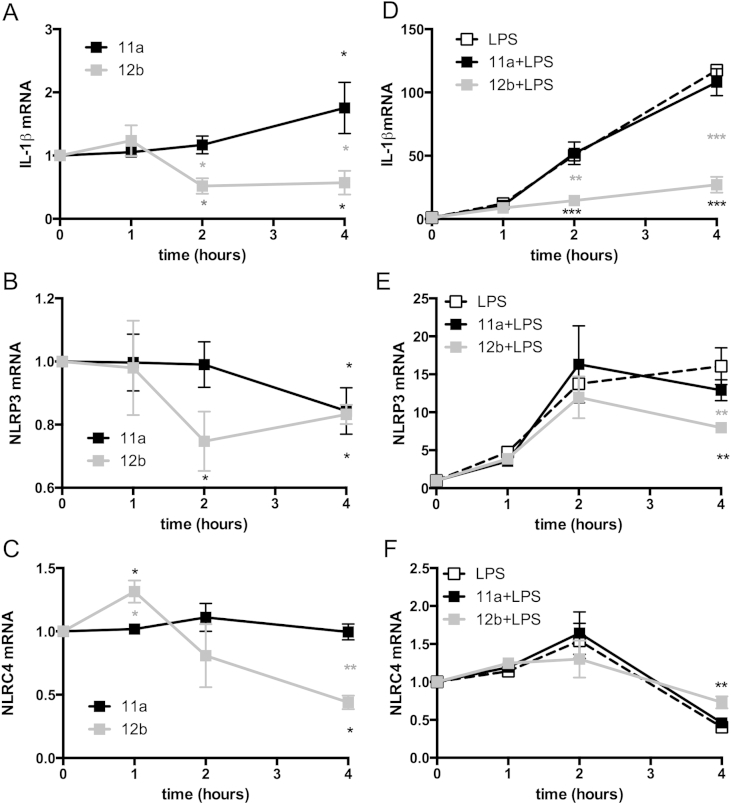
*SMA-12b downregulates genes associated with production of bioactive IL-1β in bmMs*. The effect of exposure of bmMs to SMAs-11a and -12b (both at 5 μg/ml) over 4 h on the steady state- and LPS-induced mRNA levels of IL-1β (A & D); NLRP3 (B & E) and NLRC4 (C & F) as assessed by qRT-PCR where the levels of the gene of interest were normalized to the level of GAPDH and expressed as a fold change with respect to the medium control. Data are presented as the means ± SEM of the mean of replicate values pooled from 3 individual experiments. *p < 0.05; **p < 0.01 and ***p < 0.001. Black* represent significance between 12b (or 11a) and control whereas grey* represents significant differences between 12b- and 11a-treated cells.

**Fig. 6:**
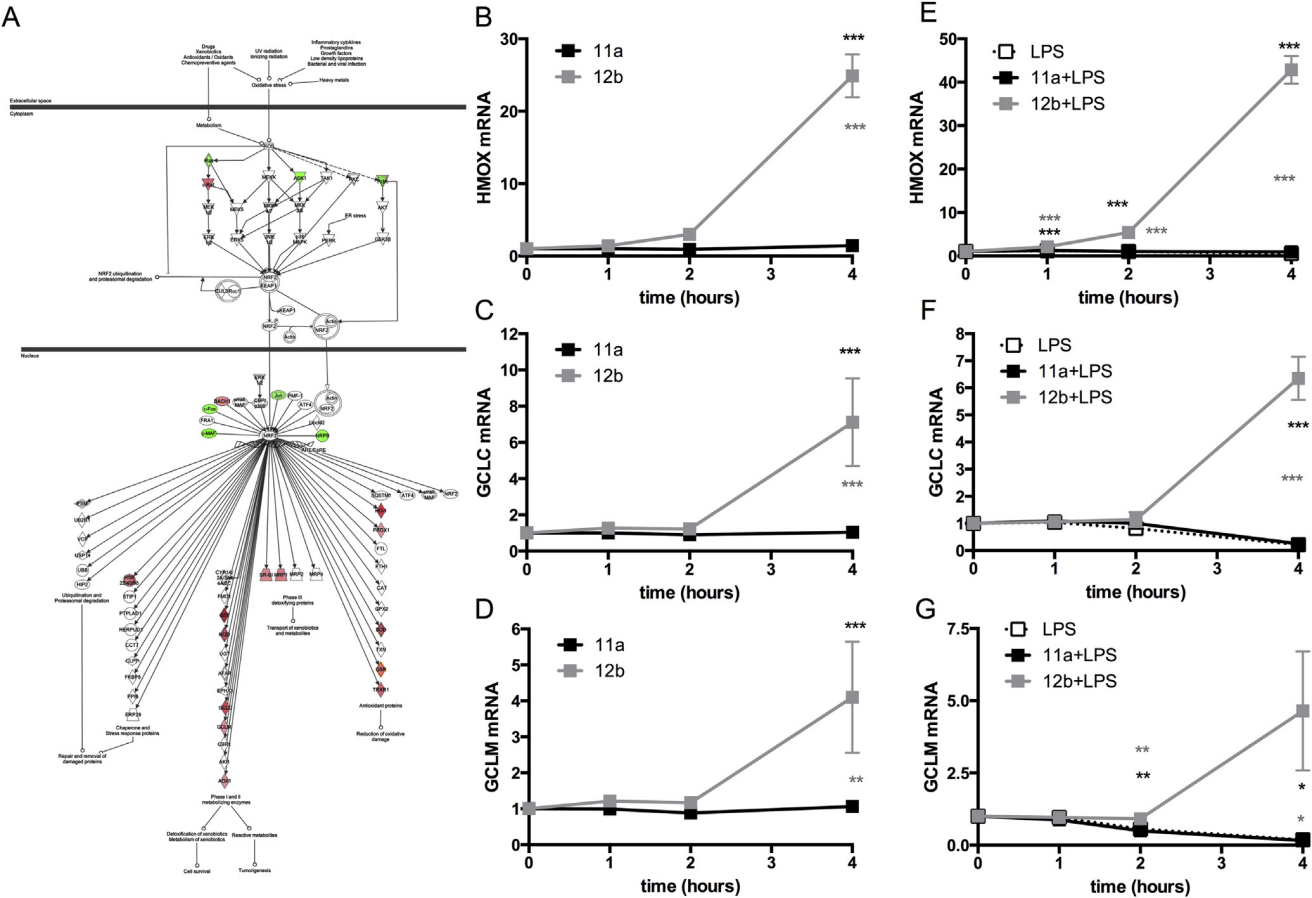
*SMA-12b upregulates mRNA levels of anti-oxidant genes that are NRF2 targets*. (A) IPA prediction of 12b-mediated activation of NRF2 cytoprotective/anti-oxidant pathways based on 12b-modulation of expression of NRF2 target genes as assessed by microarray analysis. The effect of exposure of bmMs to SMAs-11a and -12b over 4 h (both at 5 μg/ml) on the steady state- and LPS-induced mRNA levels of HMOX1 (B & E); GCLC (C & F) and GCLM (D & G) as assessed by qRT-PCR where the levels of the gene of interest were normalized to the level of GAPDH and expressed as a fold change with respect to the medium control. Data are presented as the means ± SEM of values pooled from 3 individual experiments. *p < 0.05; **p < 0.01 and ***p < 0.001. Black* represent significance between 12b (or 11a) and control whereas grey* represents significant differences between 12b- and 11a-treated cells.

**Fig. 7:**
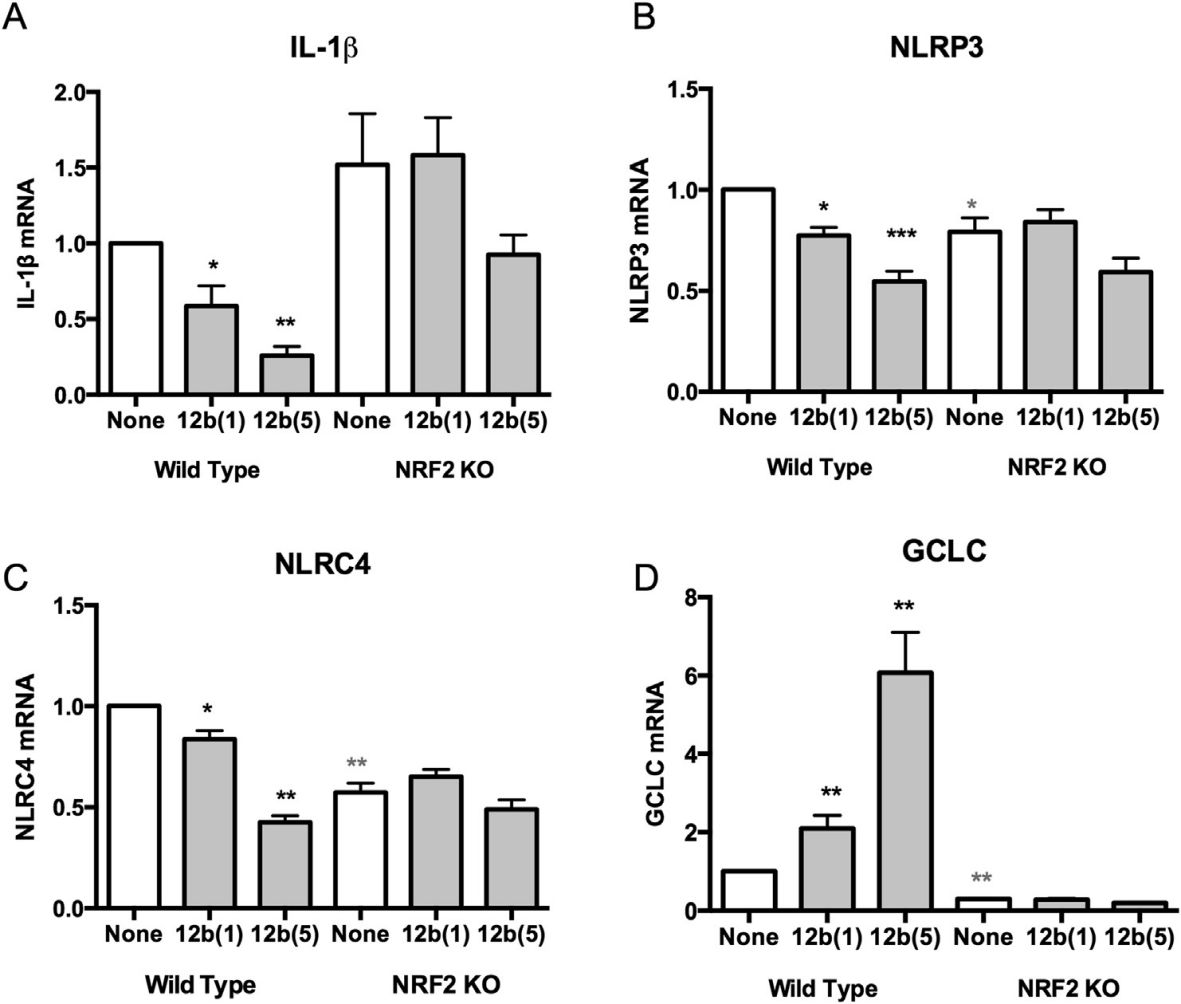
*SMA-12b-mediated changes in gene expression are abrogated in NRF2-deficient bmMs*. The effect of exposure (4 h) of bmMs from wild type and NRF2-deficient (NRF2 KO) C57BL/6 mice to SMA-12b on the mRNA levels of IL-1β (A; n = 5); NLRP3 (B; n = 5); NLRC4 (C; n = 6) and GCLC (D; n = 6) as assessed by qRT-PCR. The levels of the gene of interest were normalized to GAPDH and expressed as a fold change with respect to the relevant wild type medium control. Data are presented as the means ± SEM, where n represents matched replicate cultures of individual wild type and KO mice. *p < 0.05; **p < 0.01 and ***p < 0.001 where significance is for WT SMA treatments relative to the wild type “none” condition as indicated by black* and grey* indicates significance between “none” WT and “none” KO samples.

**Fig. 8:**
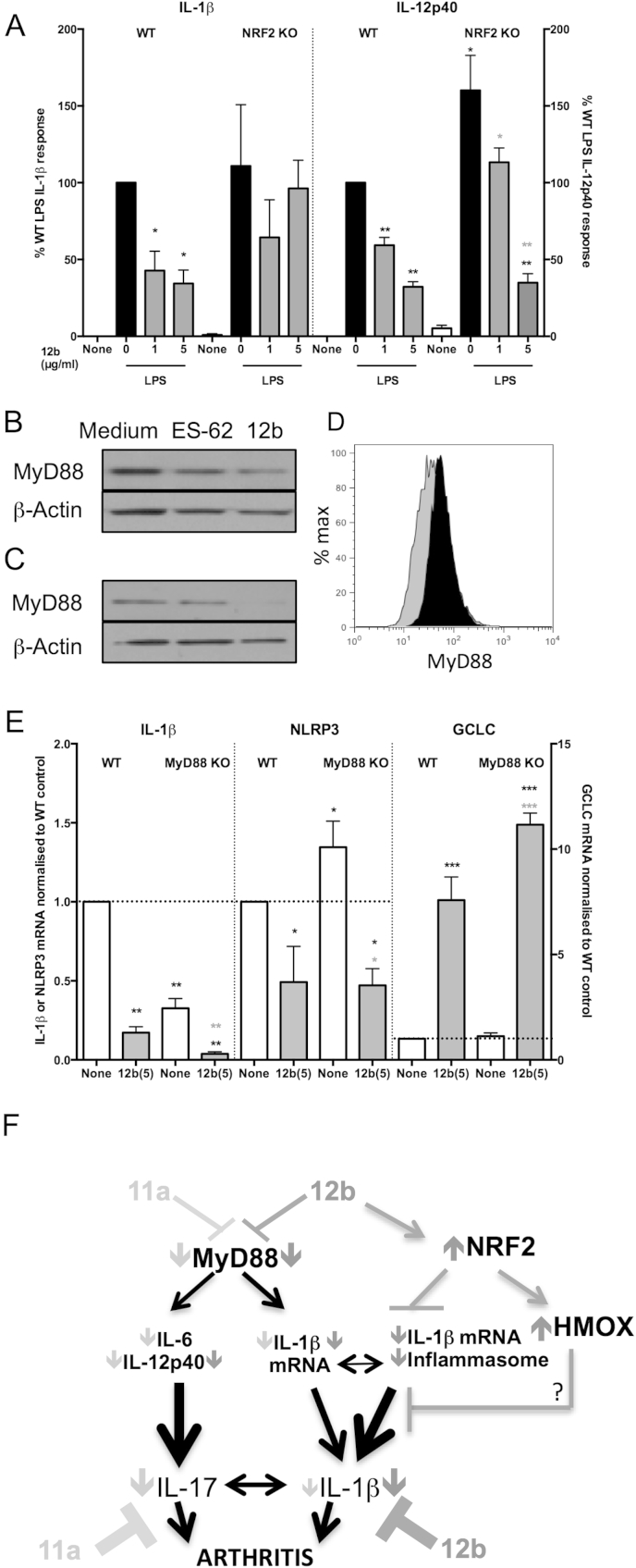
*SMA-12b-mediated suppression IL-1β is abrogated in NRF2 KO but not MyD88 KO bmMs*. (A) BmMs from wild type and NRF2-deficient (WT and NRF2 KO) C57BL/6 mice were incubated with 12b (18 h) prior to exposure to medium (“None”) or LPS (100 ng/ml) for a further 24 h and IL-β and IL-12p40 release measured by ELISA. Data presented are the % responses (normalised to the wild type LPS response; 100%) and the means ± SEM (of mean values of triplicate cultures) from matched individual wild type and NRF2 KO mice (IL-1β: n = 3 and IL-12p40; n = 6). Western blot analysis of MyD88 expression in BALB/c bmMs (B) treated with ES-62 (2 μg/ml) or 12b (1 μg/ml) for 20 h. bmMs pretreated with 12b (2 h) were then stimulated with LPS overnight and MyD88 expression assessed by western blotting (C) or flow cytometry (D; black = LPS; grey = LPS + SMA-12b). The effect of exposure (4 h) of bmMs from wild type and MyD88-deficient (MyD88 KO) C57BL/6 mice to 12b (E) on the mRNA levels of IL-1β (n = 4); NLRP3 (n = 3) and GCLC (n = 6) as assessed by qRT-PCR. The levels of the genes were normalized to GAPDH and expressed as a fold change with respect to the relevant WT medium control. Data (A & E) are presented as the means ± SEM, where n represents matched cultures from individual wild type and KO mice. *p < 0.05; **p < 0.01 and ***p < 0.001 where for black *, significance is relative to the corresponding wild type control and for grey**, significance is relative to the corresponding KO control. (F) Model of SMA-12b action in CIA: 12b protection predominantly reflects activation of NRF2 signalling to counteract MyD88-integrated inflammasome-mediated IL-1β production whilst 11a preferentially targets MyD88-driven induction of the IL-17 inflammatory axis.
