# Supplementary material for: Prophylactic and therapeutic treatment with a synthetic analogue of a parasitic worm product prevents experimental arthritis and inhibits IL-1β production via NRF2-mediated counter-regulation of the inflammasome
Source: J Autoimmun. 2015 Jun;60:59–73. doi: 10.1016/j.jaut.2015.04.005 (PMC4459730; doi:10.1016/j.jaut.2015.04.005)
Supplement: Supplementary file 1 [file mmc1.docx]

Supplementary Table 1: list of genes affected by SMA12b and associated IPA information

| p-value | Fold Change | ID | Symbol | Entrez Gene Name | Location | Type(s) |
| --- | --- | --- | --- | --- | --- | --- |
| 2.29E-05 | 1.705 | 10455967 | 2610318N02Rik | RIKEN cDNA 2610318N02 gene | unknown | other |
| 2.35E-04 | 1.525 | 10497481 | 2810416G20Rik | RIKEN cDNA 2810416G20 gene | unknown | other |
| 6.62E-05 | 2.400 | 10459643 | 4930503L19Rik | RIKEN cDNA 4930503L19 gene | Extracellular Space | other |
| 2.24E-05 | 2.098 | 10349661 | 5430435G22Rik | RIKEN cDNA 5430435G22 gene | Cytoplasm | peptidase |
| 1.35E-04 | 1.801 | 10568758 | 9430038I01Rik | RIKEN cDNA 9430038I01 gene | unknown | other |
| 5.38E-05 | 2.806 | 10451646 | 9830107B12Rik (includes others) | RIKEN cDNA B430306N03 gene | unknown | other |
| 1.11E-04 | -1.538 | 10385526 | 9930111J21Rik2 | RIKEN cDNA 9930111J21 gene 2 | unknown | other |
| 7.10E-05 | 2.051 | 10601421 | A630033H20Rik | RIKEN cDNA A630033H20 gene | Plasma Membrane | G-protein coupled receptor |
| 3.41E-04 | -1.672 | 10532313 | AB010352 | cDNA sequence AB010352 | unknown | other |
| 3.16E-07 | 1.893 | 10433735 | ABCC1 | ATP-binding cassette, sub-family C (CFTR/MRP), member 1 | Plasma Membrane | transporter |
| 1.58E-04 | -1.665 | 10431697 | ABCD2 | ATP-binding cassette, sub-family D (ALD), member 2 | Cytoplasm | transporter |
| 3.67E-05 | -1.575 | 10579874 | ABCE1 | ATP-binding cassette, sub-family E (OABP), member 1 | Cytoplasm | transporter |
| 2.10E-04 | 1.621 | 10590452 | ABHD5 | abhydrolase domain containing 5 | Cytoplasm | enzyme |
| 2.59E-06 | -3.208 | 10390186 | ABI3 | ABI family, member 3 | Plasma Membrane | other |
| 3.14E-04 | -1.716 | 10511226 | ACAP3 | ArfGAP with coiled-coil, ankyrin repeat and PH domains 3 | Nucleus | transcription regulator |
| 1.83E-06 | 4.705 | 10505779 | ACER2 | alkaline ceramidase 2 | Cytoplasm | enzyme |
| 8.42E-07 | 2.740 | 10397172 | Acot6 | acyl-CoA thioesterase 6 | Cytoplasm | enzyme |
| 1.42E-05 | -2.104 | 10571657 | ACSL1 | acyl-CoA synthetase long-chain family member 1 | Cytoplasm | enzyme |
| 6.77E-06 | 2.460 | 10477649 | ACSS2 | acyl-CoA synthetase short-chain family member 2 | Cytoplasm | enzyme |
| 1.36E-05 | 2.552 | 10426999 | ACVRL1 | activin A receptor type II-like 1 | Plasma Membrane | kinase |
| 2.16E-04 | -2.467 | 10443949 | ADAMTS10 | ADAM metallopeptidase with thrombospondin type 1 motif, 10 | Extracellular Space | peptidase |
| 3.75E-04 | -1.626 | 10437364 | ADCY9 | adenylate cyclase 9 | Plasma Membrane | enzyme |
| 1.76E-04 | 3.336 | 10496425 | ADH7 | alcohol dehydrogenase 7 (class IV), mu or sigma polypeptide | Cytoplasm | enzyme |
| 4.96E-05 | -2.299 | 10376832 | ADORA2B | adenosine A2b receptor | Plasma Membrane | G-protein coupled receptor |
| 6.21E-06 | -2.157 | 10459288 | ADRB2 | adrenoceptor beta 2, surface | Plasma Membrane | G-protein coupled receptor |
| 2.94E-04 | -1.514 | 10520574 | AGBL5 | ATP/GTP binding protein-like 5 | Cytoplasm | peptidase |
| 1.45E-04 | -1.721 | 10534889 | AGFG2 | ArfGAP with FG repeats 2 | Nucleus | other |
| 5.03E-06 | -2.870 | 10410604 | AHRR | aryl-hydrocarbon receptor repressor | Nucleus | other |
| 5.86E-05 | -2.394 | 10495186 | AI504432 | expressed sequence AI504432 | unknown | other |
| 1.05E-05 | -2.069 | 10368947 | AIM1 (includes EG:11630) | absent in melanoma 1 | Extracellular Space | other |
| 8.73E-05 | -1.565 | 10519420 | AKAP9 | A kinase (PRKA) anchor protein (yotiao) 9 | Cytoplasm | other |
| 1.70E-05 | 1.960 | 10556208 | AKIP1 | A kinase (PRKA) interacting protein 1 | Nucleus | other |
| 6.99E-06 | -1.700 | 10513666 | AKNA | AT-hook transcription factor | Nucleus | other |
| 2.39E-05 | -3.885 | 10502052 | ALPK1 | alpha-kinase 1 | unknown | kinase |
| 2.59E-07 | 3.145 | 10556302 | AMPD3 | adenosine monophosphate deaminase 3 | Cytoplasm | enzyme |
| 3.12E-04 | -1.849 | 10450038 | ANGPTL4 | angiopoietin-like 4 | Extracellular Space | other |
| 5.11E-05 | -1.933 | 10443244 | ANKS1A | ankyrin repeat and sterile alpha motif domain containing 1A | Cytoplasm | other |
| 1.91E-04 | 1.593 | 10346374 | AOX1 | aldehyde oxidase 1 | Cytoplasm | enzyme |
| 1.95E-04 | -1.824 | 10373986 | AP1B1 | adaptor-related protein complex 1, beta 1 subunit | Cytoplasm | transporter |
| 7.40E-07 | 2.556 | 10594638 | Aph1c | anterior pharynx defective 1c homolog (C. elegans) | unknown | peptidase |

| 1.91E-04 | -1.613 | 10467637 | ARHGAP19 | Rho GTPase activating protein 19 | Cytoplasm | other |
| --- | --- | --- | --- | --- | --- | --- |
| 6.38E-05 | -2.878 | 10413951 | ARHGAP22 | Rho GTPase activating protein 22 | Cytoplasm | other |
| 1.26E-04 | -1.715 | 10605143 | ARHGAP4 | Rho GTPase activating protein 4 | Cytoplasm | other |
| 8.51E-05 | -1.710 | 10517744 | ARHGEF10L | Rho guanine nucleotide exchange factor (GEF) 10-like | Cytoplasm | enzyme |
| 6.24E-06 | -1.694 | 10556463 | ARNTL | aryl hydrocarbon receptor nuclear translocator-like | Nucleus | transcription regulator |
| 2.23E-05 | -1.564 | 10555235 | ARRB1 | arrestin, beta 1 | Cytoplasm | other |
| 2.68E-04 | -1.768 | 10377804 | ARRB2 | arrestin, beta 2 | Cytoplasm | other |
| 5.43E-08 | 2.000 | 10406407 | ARRDC3 | arrestin domain containing 3 | Plasma Membrane | other |
| 9.61E-07 | 4.221 | 10564507 | ARRDC4 | arrestin domain containing 4 | unknown | other |
| 3.70E-05 | -1.584 | 10428998 | ASAP1 | ArfGAP with SH3 domain, ankyrin repeat and PH domain 1 | Plasma Membrane | other |
| 3.24E-05 | 1.545 | 10543067 | ASNS | asparagine synthetase (glutamine-hydrolyzing) | Cytoplasm | enzyme |
| 3.12E-04 | 1.507 | 10511446 | ASPH | aspartate beta-hydroxylase | Cytoplasm | enzyme |
| 2.95E-04 | 1.990 | 10363541 | ASS1 | argininosuccinate synthase 1 | Cytoplasm | enzyme |
| 1.85E-05 | 2.136 | 10428763 | ATAD2 | ATPase family, AAA domain containing 2 | Nucleus | other |
| 1.34E-04 | -1.646 | 10394366 | ATAD2B | ATPase family, AAA domain containing 2B | unknown | other |
| 4.42E-05 | 1.534 | 10426875 | ATF1 (includes EG:100040260) | activating transcription factor 1 | Nucleus | transcription regulator |
| 2.14E-04 | -1.800 | 10355763 | ATG9A | autophagy related 9A | Cytoplasm | other |
| 5.45E-05 | -1.733 | 10357833 | ATP2B4 | ATPase, Ca++ transporting, plasma membrane 4 | Plasma Membrane | transporter |
| 4.15E-06 | 2.303 | 10511779 | ATP6V0D2 | ATPase, H+ transporting, lysosomal 38kDa, V0 subunit d2 | Cytoplasm | transporter |
| 1.94E-05 | 1.641 | 10482330 | ATP6V1G1 | ATPase, H+ transporting, lysosomal 13kDa, V1 subunit G1 | Cytoplasm | transporter |
| 2.66E-05 | 1.712 | 10344637 | ATP6V1H | ATPase, H+ transporting, lysosomal 50/57kDa, V1 subunit H | Cytoplasm | transporter |
| 1.19E-05 | 1.934 | 10601360 | ATP7A | ATPase, Cu++ transporting, alpha polypeptide | Plasma Membrane | transporter |
| 7.37E-05 | -1.529 | 10476207 | ATRN | attractin | Extracellular Space | other |
| 7.41E-05 | -1.759 | 10525313 | ATXN2 | ataxin 2 | Nucleus | other |
| 9.98E-05 | -1.694 | 10377405 | AURKB | aurora kinase B | Nucleus | kinase |
| 5.84E-05 | 1.669 | 10436734 | BACH1 | BTB and CNC homology 1, basic leucine zipper transcription factor 1 | Nucleus | transcription regulator |
| 1.72E-05 | 1.694 | 10439409 | BC031361 | cDNA sequence BC031361 | unknown | other |
| 3.09E-07 | -2.646 | 10560685 | BCL3 | B-cell CLL/lymphoma 3 | Nucleus | transcription regulator |
| 4.98E-05 | -1.777 | 10584762 | BCL9L | B-cell CLL/lymphoma 9-like | Cytoplasm | other |
| 9.38E-05 | -2.333 | 10603627 | BCOR | BCL6 corepressor | Nucleus | transcription regulator |
| 7.83E-06 | 2.216 | 10549276 | BHLHE41 | basic helix-loop-helix family, member e41 | Nucleus | transcription regulator |
| 1.96E-04 | -1.586 | 10429908 | BOP1 | block of proliferation 1 | Nucleus | other |
| 1.76E-04 | -1.659 | 10537179 | BPGM | 2,3-bisphosphoglycerate mutase | Extracellular Space | phosphatase |
| 2.65E-05 | -1.901 | 10395831 | BRMS1L | breast cancer metastasis-suppressor 1-like | unknown | other |
| 1.02E-04 | 1.695 | 10521667 | BST1 (includes EG:12182) | bone marrow stromal cell antigen 1 | Plasma Membrane | enzyme |
| 1.27E-04 | -1.660 | 10357875 | BTG2 | BTG family, member 2 | Nucleus | transcription regulator |
| 1.79E-05 | 3.751 | 10440419 | BTG3 | BTG family, member 3 | Nucleus | other |
| 5.12E-05 | -1.731 | 10451580 | BYSL | bystin-like | Cytoplasm | other |
| 9.03E-08 | -1.627 | 10397763 | C14orf159 | chromosome 14 open reading frame 159 | Cytoplasm | other |
| 4.22E-05 | -2.224 | 10398920 | C14orf79 | chromosome 14 open reading frame 79 | unknown | other |
| 3.14E-04 | -1.630 | 10569877 | C19orf59 | chromosome 19 open reading frame 59 | unknown | other |
| 9.25E-07 | -4.238 | 10516966 | C1orf38 | chromosome 1 open reading frame 38 | unknown | other |
| 2.65E-04 | 2.227 | 10359446 | C1orf9 | chromosome 1 open reading frame 9 | unknown | other |

| 2.94E-04 | 1.509 | 10489364 | C20orf111 | chromosome 20 open reading frame 111 | unknown | kinase |
| --- | --- | --- | --- | --- | --- | --- |
| 2.70E-04 | -1.523 | 10592802 | C2CD2L | C2CD2-like | unknown | other |
| 4.52E-06 | -1.713 | 10438572 | C3orf70 | chromosome 3 open reading frame 70 | unknown | other |
| 1.06E-06 | -4.200 | 10560242 | C5AR1 | complement component 5a receptor 1 | Plasma Membrane | G-protein coupled receptor |
| 1.97E-04 | 1.659 | 10407387 | C5orf28 | chromosome 5 open reading frame 28 | unknown | other |
| 1.28E-05 | 2.287 | 10538408 | C7orf41 | chromosome 7 open reading frame 41 | unknown | other |
| 2.49E-05 | -2.314 | 10535006 | C7orf43 | chromosome 7 open reading frame 43 | unknown | other |
| 2.83E-04 | -1.707 | 10513397 | C9orf80 | chromosome 9 open reading frame 80 | Nucleus | other |
| 1.51E-05 | 1.632 | 10505465 | C9orf91 | chromosome 9 open reading frame 91 | Cytoplasm | other |
| 2.82E-05 | 1.583 | 10490903 | CA13 | carbonic anhydrase XIII | unknown | enzyme |
| 5.89E-05 | -1.795 | 10390542 | CACNB1 | calcium channel, voltage-dependent, beta 1 subunit | Plasma Membrane | ion channel |
| 2.39E-04 | -2.276 | 10468294 | CALHM2 | calcium homeostasis modulator 2 | unknown | other |
| 1.68E-07 | -1.999 | 10417972 | CAMK2G | calcium/calmodulin-dependent protein kinase II gamma | Cytoplasm | kinase |
| 7.58E-05 | -1.787 | 10533529 | CAMKK2 | calcium/calmodulin-dependent protein kinase kinase 2, beta | Cytoplasm | kinase |
| 1.01E-04 | 2.242 | 10436978 | CBR3 | carbonyl reductase 3 | Cytoplasm | enzyme |
| 1.81E-04 | -1.646 | 10393620 | CBX4 | chromobox homolog 4 | Nucleus | transcription regulator |
| 2.04E-04 | -1.822 | 10393614 | CBX8 | chromobox homolog 8 | Nucleus | other |
| 1.70E-04 | -2.210 | 10557439 | CCDC101 | coiled-coil domain containing 101 | Nucleus | other |
| 1.84E-04 | -1.710 | 10406905 | CCDC125 | coiled-coil domain containing 125 | unknown | other |
| 2.80E-05 | -1.899 | 10425686 | CCDC134 | coiled-coil domain containing 134 | unknown | other |
| 2.77E-05 | 1.719 | 10389231 | CCL3L1/CCL3L3 | chemokine (C-C motif) ligand 3-like 1 | Extracellular Space | cytokine |
| 2.27E-06 | -3.758 | 10590631 | CCR2 | chemokine (C-C motif) receptor 2 | Plasma Membrane | G-protein coupled receptor |
| 6.51E-06 | -5.750 | 10590635 | CCR5 | chemokine (C-C motif) receptor 5 (gene/pseudogene) | Plasma Membrane | G-protein coupled receptor |
| 2.93E-06 | -3.169 | 10491915 | CCRN4L | CCR4 carbon catabolite repression 4-like (S. cerevisiae) | Nucleus | transcription regulator |
| 1.80E-05 | 1.642 | 10587383 | CD109 | CD109 molecule | Plasma Membrane | other |
| 2.71E-08 | 2.589 | 10435907 | CD200R1 | CD200 receptor 1 | Plasma Membrane | other |
| 3.54E-07 | 2.857 | 10462390 | CD274 | CD274 molecule | Plasma Membrane | enzyme |
| 1.02E-06 | -2.734 | 10392845 | CD300LF | CD300 molecule-like family member f | unknown | other |
| 4.65E-06 | 1.766 | 10351658 | CD48 | CD48 molecule | Plasma Membrane | other |
| 5.17E-05 | -1.716 | 10435704 | CD80 (includes EG:12519) | CD80 molecule | Plasma Membrane | transmembrane receptor |
| 2.70E-04 | -1.667 | 10580033 | CD97 | CD97 molecule | Plasma Membrane | G-protein coupled receptor |
| 4.58E-05 | 1.661 | 10410099 | CDC14B | CDC14 cell division cycle 14 homolog B (S. cerevisiae) | Nucleus | phosphatase |
| 1.19E-05 | -2.343 | 10453049 | CDC42EP3 | CDC42 effector protein (Rho GTPase binding) 3 | Cytoplasm | other |
| 9.83E-05 | -1.652 | 10369815 | CDK1 | cyclin-dependent kinase 1 | Nucleus | kinase |
| 2.74E-05 | -1.838 | 10581996 | CDYL2 | chromodomain protein, Y-like 2 | Nucleus | other |
| 3.20E-05 | -1.747 | 10359080 | CEP350 | centrosomal protein 350kDa | Cytoplasm | other |
| 5.18E-05 | -1.914 | 10466423 | CEP78 | centrosomal protein 78kDa | Cytoplasm | other |
| 1.11E-04 | -1.614 | 10472501 | CERS6 | ceramide synthase 6 | Nucleus | transcription regulator |
| 1.78E-05 | -1.558 | 10541803 | CHD4 | chromodomain helicase DNA binding protein 4 | Nucleus | enzyme |
| 1.03E-04 | -2.381 | 10503196 | CHD7 | chromodomain helicase DNA binding protein 7 | Nucleus | enzyme |
| 1.47E-04 | -1.951 | 10573823 | CHD9 | chromodomain helicase DNA binding protein 9 | Cytoplasm | other |
| 1.34E-05 | 2.300 | 10524266 | CHEK2 | checkpoint kinase 2 | Nucleus | kinase |
| 4.76E-05 | -1.672 | 10369301 | CHST3 | carbohydrate (chondroitin 6) sulfotransferase 3 | Cytoplasm | enzyme |
| 1.28E-04 | -1.552 | 10467921 | CHUK | conserved helix-loop-helix ubiquitous kinase | Cytoplasm | kinase |
| 1.67E-04 | -1.538 | 10544273 | CLEC5A | C-type lectin domain family 5, member A | Plasma Membrane | other |

| 1.03E-06 | 2.035 | 10541605 | CLEC6A | C-type lectin domain family 6, member A | Plasma Membrane | other |
| --- | --- | --- | --- | --- | --- | --- |
| 2.51E-04 | -1.514 | 10584561 | CLMP | CXADR-like membrane protein | Plasma Membrane | other |
| 1.08E-07 | 2.794 | 10416843 | CLN5 | ceroid-lipofuscinosis, neuronal 5 | Cytoplasm | other |
| 1.48E-04 | 1.690 | 10570472 | CLN8 | ceroid-lipofuscinosis, neuronal 8 (epilepsy, progressive with mental retardation) | Cytoplasm | other |
| 9.86E-05 | -2.050 | 10532711 | CMKLR1 | chemokine-like receptor 1 | Plasma Membrane | G-protein coupled receptor |
| 3.80E-04 | 1.552 | 10460085 | CNDP2 | CNDP dipeptidase 2 (metallopeptidase M20 family) | Cytoplasm | peptidase |
| 8.35E-05 | -2.023 | 10463716 | CNNM2 | cyclin M2 | Plasma Membrane | other |
| 1.68E-04 | -2.345 | 10509122 | CNR2 | cannabinoid receptor 2 (macrophage) | Plasma Membrane | G-protein coupled receptor |
| 2.49E-05 | 4.773 | 10374406 | CNRIP1 | cannabinoid receptor interacting protein 1 | unknown | other |
| 2.78E-05 | -1.949 | 10382345 | COG1 (includes EG:100334475) | component of oligomeric golgi complex 1 | Cytoplasm | transporter |
| 2.39E-04 | 1.831 | 10572378 | COMP | cartilage oligomeric matrix protein | Extracellular Space | other |
| 1.94E-04 | -1.606 | 10524668 | COQ5 | coenzyme Q5 homolog, methyltransferase (S. cerevisiae) | Cytoplasm | enzyme |
| 3.96E-05 | -2.231 | 10568024 | CORO1A | coronin, actin binding protein, 1A | Cytoplasm | other |
| 5.29E-06 | 2.241 | 10375002 | CPEB4 | cytoplasmic polyadenylation element binding protein 4 | Plasma Membrane | other |
| 1.23E-07 | -1.510 | 10478799 | CSE1L | CSE1 chromosome segregation 1-like (yeast) | Nucleus | transporter |
| 1.81E-05 | -1.505 | 10593966 | CSK | c-src tyrosine kinase | Cytoplasm | kinase |
| 2.30E-04 | -1.611 | 10344803 | CSPP1 | centrosome and spindle pole associated protein 1 | Cytoplasm | other |
| 7.75E-05 | -1.870 | 10409876 | Ctla2a/Ctla2b | cytotoxic T lymphocyte-associated protein 2 alpha | Plasma Membrane | other |
| 3.01E-04 | 1.501 | 10388241 | CTNS | cystinosin, lysosomal cystine transporter | Cytoplasm | transporter |
| 4.00E-05 | 1.838 | 10494262 | CTSK | cathepsin K | Cytoplasm | peptidase |
| 5.65E-05 | -1.605 | 10500948 | CTTNBP2NL | CTTNBP2 N-terminal like | Cytoplasm | other |
| 2.86E-07 | -3.701 | 10597743 | CX3CR1 | chemokine (C-X3-C motif) receptor 1 | Plasma Membrane | G-protein coupled receptor |
| 5.31E-05 | -4.256 | 10531415 | CXCL10 | chemokine (C-X-C motif) ligand 10 | Extracellular Space | cytokine |
| 3.11E-05 | -1.690 | 10409579 | CXCL14 | chemokine (C-X-C motif) ligand 14 | Extracellular Space | cytokine |
| 1.54E-05 | -1.979 | 10523156 | CXCL3 | chemokine (C-X-C motif) ligand 3 | Extracellular Space | cytokine |
| 7.26E-06 | 1.715 | 10457071 | CYB5A | cytochrome b5 type A (microsomal) | Cytoplasm | enzyme |
| 5.73E-05 | 1.535 | 10578448 | CYP4V2 | cytochrome P450, family 4, subfamily V, polypeptide 2 | Cytoplasm | enzyme |
| 7.68E-05 | -1.617 | 10606355 | CYSLTR1 | cysteinyl leukotriene receptor 1 | Plasma Membrane | G-protein coupled receptor |
| 9.56E-06 | -1.828 | 10425092 | CYTH4 | cytohesin 4 | Cytoplasm | other |
| 1.00E-07 | -2.741 | 10482802 | CYTIP | cytohesin 1 interacting protein | Cytoplasm | other |
| 3.26E-04 | -1.557 | 10430645 | D730005E14Rik | RIKEN cDNA D730005E14 gene | unknown | other |
| 1.30E-04 | -1.698 | 10422728 | DAB2 | disabled homolog 2, mitogen-responsive phosphoprotein (Drosophila) | Plasma Membrane | other |
| 1.58E-04 | -1.992 | 10580085 | DCAF15 | DDB1 and CUL4 associated factor 15 | unknown | other |
| 5.31E-06 | -2.739 | 10391831 | DCAKD | dephospho-CoA kinase domain containing | Cytoplasm | other |
| 1.54E-05 | 2.274 | 10436372 | DCBLD2 | discoidin, CUB and LCCL domain containing 2 | Plasma Membrane | other |
| 2.63E-07 | 5.595 | 10523012 | DCK | deoxycytidine kinase | Nucleus | kinase |
| 3.04E-04 | -2.001 | 10522430 | DCUN1D4 | DCN1, defective in cullin neddylation 1, domain containing 4 (S. cerevisiae) | Nucleus | other |
| 3.83E-05 | -2.281 | 10369630 | DDX21 | DEAD (Asp-Glu-Ala-Asp) box helicase 21 | Nucleus | enzyme |
| 4.57E-05 | -1.788 | 10581450 | DDX28 | DEAD (Asp-Glu-Ala-Asp) box polypeptide 28 | Nucleus | enzyme |
| 1.20E-04 | -1.607 | 10470628 | DDX31 | DEAD (Asp-Glu-Ala-Asp) box polypeptide 31 | Nucleus | enzyme |
| 5.30E-06 | -2.239 | 10512067 | DDX58 | DEAD (Asp-Glu-Ala-Asp) box polypeptide 58 | Cytoplasm | enzyme |
| 1.45E-05 | 1.659 | 10409031 | DEK | DEK oncogene | Nucleus | transcription regulator |
| 9.88E-06 | -3.751 | 10586250 | DENND4A | DENN/MADD domain containing 4A | Nucleus | other |
| 6.56E-05 | 2.044 | 10565775 | DGAT2 | diacylglycerol O-acyltransferase 2 | Cytoplasm | enzyme |

| 5.49E-05 | -1.508 | 10373542 | DGKA | diacylglycerol kinase, alpha 80kDa | Cytoplasm | kinase |
| --- | --- | --- | --- | --- | --- | --- |
| 7.49E-06 | 1.845 | 10401841 | DIO2 | deiodinase, iodothyronine, type II | Cytoplasm | enzyme |
| 4.04E-06 | 1.956 | 10426835 | DIP2B | DIP2 disco-interacting protein 2 homolog B (Drosophila) | unknown | other |
| 8.12E-06 | -1.927 | 10363575 | DNA2 | DNA replication helicase 2 homolog (yeast) | Cytoplasm | enzyme |
| 3.53E-06 | 2.778 | 10502823 | DNAJB4 | DnaJ (Hsp40) homolog, subfamily B, member 4 | Nucleus | other |
| 6.95E-05 | 1.712 | 10400137 | DNAJB9 | DnaJ (Hsp40) homolog, subfamily B, member 9 | Nucleus | other |
| 8.17E-06 | -1.726 | 10462140 | DOCK8 | dedicator of cytokinesis 8 | Cytoplasm | other |
| 3.08E-06 | -2.550 | 10364856 | DOT1L | DOT1-like, histone H3 methyltransferase (S. cerevisiae) | Nucleus | phosphatase |
| 1.26E-06 | 1.742 | 10408915 | DTNBP1 | dystrobrevin binding protein 1 | Plasma Membrane | other |
| 2.66E-04 | -1.700 | 10574944 | DUS2L | dihydrouridine synthase 2-like, SMM1 homolog (S. cerevisiae) | Cytoplasm | other |
| 2.91E-04 | 1.922 | 10418016 | DUSP13 | dual specificity phosphatase 13 | Cytoplasm | phosphatase |
| 2.47E-04 | -2.151 | 10548735 | DUSP16 | dual specificity phosphatase 16 | Nucleus | phosphatase |
| 5.95E-05 | 2.291 | 10571312 | DUSP4 | dual specificity phosphatase 4 | Nucleus | phosphatase |
| 2.08E-05 | -2.099 | 10588495 | DUSP7 | dual specificity phosphatase 7 | Cytoplasm | phosphatase |
| 4.90E-05 | 1.572 | 10589913 | DYNC1LI1 | dynein, cytoplasmic 1, light intermediate chain 1 | Cytoplasm | other |
| 2.18E-07 | 14.373 | 10422164 | EDNRB | endothelin receptor type B | Plasma Membrane | G-protein coupled receptor |
| 2.45E-04 | -1.534 | 10582712 | EGLN1 | egl nine homolog 1 (C. elegans) | Cytoplasm | other |
| 2.16E-05 | -1.966 | 10400304 | EGLN3 | egl nine homolog 3 (C. elegans) | Cytoplasm | enzyme |
| 2.70E-06 | 2.420 | 10363735 | EGR2 | early growth response 2 | Nucleus | transcription regulator |
| 8.04E-06 | -1.573 | 10486396 | EHD4 | EH-domain containing 4 | Plasma Membrane | enzyme |
| 5.43E-06 | 1.926 | 10429327 | EIF2C2 | eukaryotic translation initiation factor 2C, 2 | Cytoplasm | translation regulator |
| 4.62E-05 | 1.568 | 10498038 | ELF2 | E74-like factor 2 (ets domain transcription factor) | Nucleus | transcription regulator |
| 4.98E-06 | -1.687 | 10406254 | ELL2 | elongation factor, RNA polymerase II, 2 | Nucleus | transcription regulator |
| 2.67E-05 | -1.726 | 10489660 | ELMO2 | engulfment and cell motility 2 | Cytoplasm | other |
| 3.36E-06 | -2.247 | 10447141 | EML4 | echinoderm microtubule associated protein like 4 | Cytoplasm | other |
| 1.37E-06 | -3.055 | 10406817 | ENC1 | ectodermal-neural cortex 1 (with BTB-like domain) | Nucleus | peptidase |
| 3.06E-04 | -1.529 | 10383235 | ENDOV | endonuclease V | unknown | enzyme |
| 5.24E-07 | -1.993 | 10383032 | ENGASE | endo-beta-N-acetylglucosaminidase | unknown | enzyme |
| 1.12E-05 | 1.537 | 10368289 | ENPP1 | ectonucleotide pyrophosphatase/phosphodiesterase 1 | Plasma Membrane | enzyme |
| 2.70E-04 | -1.610 | 10476952 | ENTPD6 | ectonucleoside triphosphate diphosphohydrolase 6 (putative) | Cytoplasm | enzyme |
| 1.16E-04 | -1.627 | 10477854 | EPB41L1 | erythrocyte membrane protein band 4.1-like 1 | Plasma Membrane | other |
| 5.55E-05 | -1.757 | 10548905 | EPS8 | epidermal growth factor receptor pathway substrate 8 | Plasma Membrane | peptidase |
| 1.92E-04 | -1.719 | 10547410 | ERC1 | ELKS/RAB6-interacting/CAST family member 1 | Cytoplasm | other |
| 2.25E-04 | 1.713 | 10416411 | ESD | esterase D | Cytoplasm | enzyme |
| 4.99E-05 | -1.835 | 10542275 | ETV6 | ets variant 6 | Nucleus | transcription regulator |
| 5.23E-05 | -1.539 | 10462822 | EXOC6 | exocyst complex component 6 | Plasma Membrane | transporter |
| 1.71E-05 | -1.585 | 10467688 | EXOSC1 | exosome component 1 | Nucleus | enzyme |
| 2.52E-07 | 5.935 | 10428579 | EXT1 | exostosin 1 | Cytoplasm | enzyme |
| 9.12E-06 | -2.329 | 10420837 | EXTL3 | exostoses (multiple)-like 3 | Cytoplasm | enzyme |
| 3.02E-04 | 1.929 | 10353192 | EYA1 | eyes absent homolog 1 (Drosophila) | Nucleus | phosphatase |
| 2.06E-05 | 1.645 | 10447602 | EZR | ezrin | Plasma Membrane | other |
| 1.48E-04 | 1.634 | 10570291 | F10 | coagulation factor X | Extracellular Space | peptidase |
| 1.04E-04 | -1.858 | 10351623 | F11R | F11 receptor | Plasma Membrane | other |
| 8.45E-05 | 1.671 | 10497265 | FABP4 | fatty acid binding protein 4, adipocyte | Cytoplasm | transporter |
| 3.26E-04 | -1.631 | 10465895 | FADS2 | fatty acid desaturase 2 | Plasma Membrane | enzyme |

| 3.78E-04 | 1.709 | 10382797 | FAM100B | family with sequence similarity 100, member B | unknown | other |
| --- | --- | --- | --- | --- | --- | --- |
| 2.48E-05 | -1.596 | 10501456 | FAM102B | family with sequence similarity 102, member B | unknown | other |
| 9.55E-05 | -1.585 | 10592126 | FAM118B | family with sequence similarity 118, member B | unknown | other |
| 6.14E-06 | 3.206 | 10604620 | FAM122B | family with sequence similarity 122B | unknown | other |
| 1.44E-05 | 3.337 | 10545045 | FAM13A | family with sequence similarity 13, member A | Cytoplasm | other |
| 2.63E-04 | -1.515 | 10421394 | FAM160B2 | family with sequence similarity 160, member B2 | unknown | other |
| 8.29E-05 | 1.717 | 10436951 | Fam165b | family with sequence similarity 165, member B | unknown | other |
| 5.66E-05 | -1.657 | 10555425 | FAM168A | family with sequence similarity 168, member A | unknown | other |
| 6.79E-06 | -2.387 | 10552071 | Fam187b | family with sequence similarity 187, member B | unknown | other |
| 4.59E-05 | 3.977 | 10492682 | FAM198B | family with sequence similarity 198, member B | Cytoplasm | other |
| 3.14E-06 | -2.550 | 10386916 | FAM211A | family with sequence similarity 211, member A | unknown | other |
| 4.76E-05 | 1.825 | 10541002 | FAM21A/FAM21C | family with sequence similarity 21, member C | Cytoplasm | other |
| 2.30E-06 | -1.683 | 10595404 | FAM46A | family with sequence similarity 46, member A | unknown | other |
| 1.34E-04 | -2.754 | 10568586 | FAM53B | family with sequence similarity 53, member B | unknown | other |
| 1.14E-05 | 2.570 | 10361995 | FAM54A | family with sequence similarity 54, member A | Cytoplasm | other |
| 3.67E-07 | -2.171 | 10481574 | FAM78A | family with sequence similarity 78, member A | unknown | other |
| 7.48E-06 | 2.602 | 10517967 | FBLIM1 | filamin binding LIM protein 1 | Plasma Membrane | other |
| 1.50E-05 | 2.017 | 10422067 | FBXL3 | F-box and leucine-rich repeat protein 3 | Nucleus | enzyme |
| 9.63E-05 | 2.070 | 10529801 | FBXL5 | F-box and leucine-rich repeat protein 5 | Cytoplasm | enzyme |
| 1.21E-05 | 2.502 | 10361748 | FBXO30 | F-box protein 30 | Extracellular Space | other |
| 9.55E-06 | 1.557 | 10509930 | FBXO42 | F-box protein 42 | unknown | other |
| 1.13E-04 | -1.985 | 10500335 | FCGR1A | Fc fragment of IgG, high affinity Ia, receptor (CD64) | Plasma Membrane | transmembrane receptor |
| 7.95E-05 | 1.951 | 10492971 | FCRL1 | Fc receptor-like 1 | Plasma Membrane | other |
| 2.07E-04 | -1.995 | 10446351 | FER | fer (fps/fes related) tyrosine kinase | Cytoplasm | kinase |
| 6.25E-06 | -1.981 | 10443506 | FGD2 | FYVE, RhoGEF and PH domain containing 2 | Cytoplasm | other |
| 4.02E-05 | -1.752 | 10491699 | FGF2 | fibroblast growth factor 2 (basic) | Extracellular Space | growth factor |
| 1.38E-06 | -2.251 | 10508772 | FGR | Gardner-Rasheed feline sarcoma viral (v-fgr) oncogene homolog | Nucleus | kinase |
| 1.47E-05 | -1.505 | 10507908 | FHL3 | four and a half LIM domains 3 | Plasma Membrane | other |
| 2.27E-04 | -1.905 | 10581222 | FHOD1 | formin homology 2 domain containing 1 | Nucleus | other |
| 1.97E-06 | 3.724 | 10397633 | FLRT2 | fibronectin leucine rich transmembrane protein 2 | Plasma Membrane | other |
| 2.76E-04 | -1.677 | 10381708 | FMNL1 | formin-like 1 | Cytoplasm | other |
| 2.03E-04 | 1.645 | 10472097 | FMNL2 | formin-like 2 | Cytoplasm | other |
| 1.39E-06 | -2.049 | 10432439 | FMNL3 | formin-like 3 | Cytoplasm | other |
| 5.52E-06 | 1.914 | 10498827 | FNIP2 | folliculin interacting protein 2 | Cytoplasm | other |
| 1.07E-05 | -3.078 | 10397346 | FOS | FBJ murine osteosarcoma viral oncogene homolog | Nucleus | transcription regulator |
| 4.78E-05 | -1.978 | 10383545 | FOXK2 | forkhead box K2 | Nucleus | transcription regulator |
| 1.34E-04 | 1.509 | 10402063 | FOXN3 | forkhead box N3 | Nucleus | transcription regulator |
| 1.37E-04 | -1.630 | 10451613 | FOXP4 | forkhead box P4 | Nucleus | transcription regulator |
| 9.94E-05 | 1.977 | 10503010 | FPGT | fucose-1-phosphate guanylyltransferase | Cytoplasm | enzyme |
| 2.93E-06 | 2.380 | 10468992 | FRMD4A | FERM domain containing 4A | Plasma Membrane | other |
| 5.23E-05 | -1.957 | 10546631 | FRMD4B | FERM domain containing 4B | Cytoplasm | other |
| 1.33E-04 | 2.887 | 10607950 | G530011O06Rik | RIKEN cDNA G530011O06 gene | unknown | other |
| 3.29E-05 | 2.667 | 10368700 | G630090E17Rik | RIKEN cDNA G630090E17 gene | unknown | other |
| 2.41E-05 | 1.871 | 10545130 | GADD45A | growth arrest and DNA-damage-inducible, alpha | Nucleus | other |

| 3.17E-05 | -1.605 | 10520288 | GALNT11 | UDP-N-acetyl-alpha-D-galactosamine:polypeptide N-acetylgalactosaminyltransferase 11 (GalNAc-T11) | Cytoplasm | enzyme |
| --- | --- | --- | --- | --- | --- | --- |
| 6.93E-05 | 1.824 | 10432661 | GALNT6 | UDP-N-acetyl-alpha-D-galactosamine:polypeptide N-acetylgalactosaminyltransferase 6 (GalNAc-T6) | Cytoplasm | enzyme |
| 6.96E-06 | 1.515 | 10371770 | GAS2L3 | growth arrest-specific 2 like 3 | unknown | other |
| 1.17E-04 | 1.582 | 10577164 | GAS6 | growth arrest-specific 6 | Extracellular Space | growth factor |
| 5.21E-06 | -2.885 | 10526181 | GATSL2 | GATS protein-like 2 | unknown | other |
| 3.42E-05 | -1.877 | 10512574 | GBA2 | glucosidase, beta (bile acid) 2 | Cytoplasm | enzyme |
| 4.36E-05 | 1.597 | 10436500 | GBE1 | glucan (1,4-alpha-), branching enzyme 1 | Cytoplasm | enzyme |
| 9.23E-05 | -1.655 | 10463557 | GBF1 | golgi brefeldin A resistant guanine nucleotide exchange factor 1 | Cytoplasm | other |
| 3.99E-05 | -2.366 | 10496569 | GBP7 | guanylate binding protein 7 | Cytoplasm | enzyme |
| 6.22E-08 | 3.143 | 10587266 | GCLC | glutamate-cysteine ligase, catalytic subunit | Cytoplasm | enzyme |
| 3.34E-06 | 1.560 | 10495763 | GCLM | glutamate-cysteine ligase, modifier subunit | Cytoplasm | enzyme |
| 2.21E-05 | 2.174 | 10567626 | GGA2 | golgi-associated, gamma adaptin ear containing, ARF binding protein 2 | Cytoplasm | transporter |
| 1.53E-04 | -1.848 | 10442236 | Gm10509 | zinc finger protein 51 pseudogene | unknown | other |
| 9.29E-06 | 2.103 | 10578962 | Gm10661 | predicted gene 10661 | unknown | other |
| 1.16E-06 | 4.802 | 10473109 | Gm14461 | predicted gene 14461 | unknown | other |
| 5.42E-05 | -1.995 | 10385504 | Gm5431 | predicted gene 5431 | unknown | other |
| 1.10E-05 | -3.532 | 10566583 | Gm8995 | predicted gene 8995 | unknown | other |
| 3.59E-05 | -1.720 | 10408346 | Gm9983 | predicted gene 9983 | unknown | other |
| 7.16E-05 | 1.926 | 10418480 | GNL3 | guanine nucleotide binding protein-like 3 (nucleolar) | Nucleus | other |
| 8.16E-07 | 2.203 | 10487945 | GPCPD1 | glycerophosphocholine phosphodiesterase GDE1 homolog (S. cerevisiae) | unknown | other |
| 3.39E-06 | -2.347 | 10402800 | GPR132 | G protein-coupled receptor 132 | Plasma Membrane | G-protein coupled receptor |
| 7.88E-05 | 1.856 | 10407803 | GPR137B | G protein-coupled receptor 137B | Plasma Membrane | other |
| 1.26E-04 | -1.699 | 10483679 | GPR155 | G protein-coupled receptor 155 | Plasma Membrane | G-protein coupled receptor |
| 6.17E-06 | 6.415 | 10491272 | GPR160 | G protein-coupled receptor 160 | Plasma Membrane | G-protein coupled receptor |
| 1.36E-04 | -1.723 | 10447902 | GPR31 | G protein-coupled receptor 31 | Plasma Membrane | G-protein coupled receptor |
| 2.17E-08 | -5.886 | 10397645 | GPR65 | G protein-coupled receptor 65 | Plasma Membrane | G-protein coupled receptor |
| 3.37E-07 | -6.276 | 10433101 | GPR84 | G protein-coupled receptor 84 | Plasma Membrane | G-protein coupled receptor |
| 6.78E-05 | -2.183 | 10543219 | GPR85 | G protein-coupled receptor 85 | Plasma Membrane | G-protein coupled receptor |
| 8.74E-06 | -2.038 | 10427026 | GRASP | GRP1 (general receptor for phosphoinositides 1)-associated scaffold protein | Plasma Membrane | other |
| 7.07E-05 | 2.087 | 10571274 | GSR | glutathione reductase | Cytoplasm | enzyme |
| 1.03E-06 | 3.246 | 10488879 | GSS | glutathione synthetase | Cytoplasm | enzyme |
| 5.42E-07 | 3.905 | 10345065 | GSTA3 | glutathione S-transferase alpha 3 | Cytoplasm | enzyme |
| 1.83E-05 | 1.745 | 10401882 | GTF2A1 | general transcription factor IIA, 1, 19/37kDa | Cytoplasm | transcription regulator |
| 1.80E-04 | -1.776 | 10411646 | GTF2H2 | general transcription factor IIH, polypeptide 2, 44kDa | Nucleus | transcription regulator |
| 1.93E-05 | 1.523 | 10365769 | HAL | histidine ammonia-lyase | Cytoplasm | enzyme |
| 1.76E-04 | 1.754 | 10579468 | HAUS8 | HAUS augmin-like complex, subunit 8 | Cytoplasm | other |
| 2.44E-04 | -1.604 | 10375443 | HAVCR2 | hepatitis A virus cellular receptor 2 | Plasma Membrane | other |
| 5.96E-06 | -1.550 | 10395227 | HBP1 | HMG-box transcription factor 1 | Nucleus | transcription regulator |
| 7.38E-05 | -1.812 | 10432006 | HDAC7 | histone deacetylase 7 | Nucleus | transcription regulator |
| 3.71E-06 | -1.916 | 10403511 | HEATR1 | HEAT repeat containing 1 | Nucleus | other |
| 8.51E-06 | -2.307 | 10372766 | HELB | helicase (DNA) B | Nucleus | enzyme |

| 2.30E-05 | -1.702 | 10494388 | HIST1H2BJ/HIST1H2BK | histone cluster 1, H2bk | Nucleus | other |
| --- | --- | --- | --- | --- | --- | --- |
| 1.57E-04 | -1.513 | 10404036 | HIST2H2BE (includes others) | histone cluster 2, H2be | Nucleus | other |
| 1.98E-05 | 1.766 | 10409376 | HK3 | hexokinase 3 (white cell) | Cytoplasm | kinase |
| 2.81E-04 | -1.650 | 10592816 | HMBS | hydroxymethylbilane synthase | Cytoplasm | enzyme |
| 3.56E-05 | 3.663 | 10571870 | HMGB2 | high mobility group box 2 | Nucleus | transcription regulator |
| 4.61E-05 | 1.666 | 10411332 | HMGCR | 3-hydroxy-3-methylglutaryl-CoA reductase | Cytoplasm | enzyme |
| 7.69E-05 | 2.345 | 10517141 | HMGN2 | high mobility group nucleosomal binding domain 2 | Nucleus | other |
| 9.27E-08 | 3.581 | 10572897 | HMOX1 | heme oxygenase (decycling) 1 | Cytoplasm | enzyme |
| 2.71E-04 | 1.878 | 10448803 | HN1L | hematological and neurological expressed 1-like | Cytoplasm | other |
| 2.82E-04 | 1.521 | 10378848 | HSP90AA1 | heat shock protein 90kDa alpha (cytosolic), class A member 1 | Cytoplasm | enzyme |
| 1.41E-04 | 2.129 | 10416406 | HTR2A | 5-hydroxytryptamine (serotonin) receptor 2A, G protein-coupled | Plasma Membrane | G-protein coupled receptor |
| 3.12E-05 | 2.064 | 10356305 | HTR2B | 5-hydroxytryptamine (serotonin) receptor 2B, G protein-coupled | Plasma Membrane | G-protein coupled receptor |
| 1.16E-05 | 2.162 | 10525365 | HVCN1 | hydrogen voltage-gated channel 1 | unknown | ion channel |
| 3.90E-05 | 2.642 | 10588691 | HYAL1 | hyaluronoglucosaminidase 1 | Cytoplasm | enzyme |
| 7.46E-06 | 2.077 | 10543120 | ICA1 | islet cell autoantigen 1, 69kDa | Cytoplasm | other |
| 3.92E-06 | -1.686 | 10583519 | ICAM1 | intercellular adhesion molecule 1 | Plasma Membrane | transmembrane receptor |
| 4.80E-06 | -3.308 | 10364361 | ICOSLG | inducible T-cell co-stimulator ligand | Plasma Membrane | other |
| 3.69E-04 | -1.831 | 10509163 | ID3 | inhibitor of DNA binding 3, dominant negative helix-loop-helix protein | Nucleus | transcription regulator |
| 7.13E-05 | -1.594 | 10357604 | IKBKE | inhibitor of kappa light polypeptide gene enhancer in B-cells, kinase epsilon | Cytoplasm | kinase |
| 1.34E-06 | 1.537 | 10600453 | IKBKG | inhibitor of kappa light polypeptide gene enhancer in B-cells, kinase gamma | Nucleus | kinase |
| 2.73E-05 | -1.562 | 10593050 | IL10RA | interleukin 10 receptor, alpha | Plasma Membrane | transmembrane receptor |
| 2.91E-07 | -8.934 | 10487597 | IL1B | interleukin 1, beta | Extracellular Space | cytokine |
| 5.29E-05 | -2.446 | 10557342 | IL21R | interleukin 21 receptor | Plasma Membrane | transmembrane receptor |
| 5.65E-05 | 1.751 | 10454039 | IMPACT | Impact homolog (mouse) | unknown | other |
| 2.10E-05 | 2.279 | 10587495 | IRAK1BP1 | interleukin-1 receptor-associated kinase 1 binding protein 1 | unknown | other |
| 3.16E-05 | -1.936 | 10372781 | IRAK3 | interleukin-1 receptor-associated kinase 3 | Cytoplasm | kinase |
| 2.16E-04 | -1.642 | 10376060 | IRF1 (includes EG:16362) | interferon regulatory factor 1 | Nucleus | transcription regulator |
| 6.36E-05 | -1.502 | 10571705 | IRF2 | interferon regulatory factor 2 | Nucleus | transcription regulator |
| 5.11E-06 | 2.464 | 10416837 | IRG1 | immunoresponsive 1 homolog (mouse) | unknown | other |
| 6.64E-05 | 1.569 | 10576971 | IRS2 | insulin receptor substrate 2 | Cytoplasm | enzyme |
| 1.36E-05 | -1.666 | 10433114 | ITGA5 | integrin, alpha 5 (fibronectin receptor, alpha polypeptide) | Plasma Membrane | other |
| 1.11E-04 | -2.129 | 10557591 | ITGAL | integrin, alpha L (antigen CD11A (p180), lymphocyte function-associated antigen 1; alpha polypeptide) | Plasma Membrane | other |
| 2.97E-04 | 2.194 | 10381809 | ITGB3 | integrin, beta 3 (platelet glycoprotein IIIa, antigen CD61) | Plasma Membrane | transmembrane receptor |
| 1.26E-04 | -1.697 | 10352234 | ITPKB | inositol-trisphosphate 3-kinase B | Cytoplasm | kinase |
| 3.51E-04 | -1.526 | 10436892 | ITSN1 | intersectin 1 (SH3 domain protein) | Cytoplasm | other |
| 3.57E-04 | -1.589 | 10402808 | JAG2 | jagged 2 | Extracellular Space | growth factor |
| 2.18E-05 | -2.409 | 10404848 | JARID2 | jumonji, AT rich interactive domain 2 | Nucleus | transcription regulator |
| 1.30E-04 | -2.034 | 10572456 | JUND | jun D proto-oncogene | Nucleus | transcription regulator |
| 6.46E-06 | -1.886 | 10391221 | KAT2A | K(lysine) acetyltransferase 2A | Cytoplasm | enzyme |
| 2.27E-06 | 1.667 | 10544976 | KBTBD2 | kelch repeat and BTB (POZ) domain containing 2 | unknown | other |
| 2.30E-04 | 1.519 | 10492402 | KCNAB1 | potassium voltage-gated channel, shaker-related subfamily, beta member 1 | Plasma Membrane | ion channel |
| 3.33E-05 | 2.203 | 10555297 | KCNE3 | potassium voltage-gated channel, Isk-related family, member 3 | Plasma Membrane | ion channel |
| 8.83E-05 | -2.110 | 10351781 | KCNJ10 | potassium inwardly-rectifying channel, subfamily J, member 10 | Plasma Membrane | ion channel |

| 1.61E-05 | 1.504 | 10466848 | KIAA0020 | KIAA0020 | Nucleus | other |
| --- | --- | --- | --- | --- | --- | --- |
| 2.98E-04 | -1.546 | 10576010 | KIAA0182 | KIAA0182 | Extracellular Space | other |
| 7.07E-05 | -1.893 | 10575993 | KIAA0513 | KIAA0513 | unknown | other |
| 1.17E-05 | 2.086 | 10368101 | KIAA1244 | KIAA1244 | Cytoplasm | other |
| 3.65E-08 | -4.343 | 10369453 | KIAA1274 | KIAA1274 | Cytoplasm | phosphatase |
| 4.82E-05 | 1.779 | 10453629 | KIAA1462 | KIAA1462 | Plasma Membrane | other |
| 3.57E-05 | 1.644 | 10516566 | KIAA1522 | KIAA1522 | unknown | other |
| 4.72E-06 | -1.604 | 10468762 | KIAA1598 | KIAA1598 | unknown | other |
| 9.48E-07 | 3.344 | 10397476 | KIAA1737 | KIAA1737 | unknown | other |
| 6.40E-05 | -1.538 | 10512236 | KIF24 | kinesin family member 24 | Cytoplasm | other |
| 3.30E-05 | -1.597 | 10536996 | KLHDC10 | kelch domain containing 10 | unknown | other |
| 1.06E-05 | 2.280 | 10510624 | KLHL21 | kelch-like 21 (Drosophila) | Cytoplasm | enzyme |
| 4.56E-05 | 1.998 | 10520096 | KLHL7 | kelch-like 7 (Drosophila) | Nucleus | other |
| 3.30E-04 | 1.568 | 10505839 | KLHL9 | kelch-like 9 (Drosophila) | Cytoplasm | other |
| 2.13E-06 | -2.724 | 10392284 | KPNA2 | karyopherin alpha 2 (RAG cohort 1, importin alpha 1) | Nucleus | transporter |
| 2.64E-05 | -1.563 | 10519392 | KRIT1 | KRIT1, ankyrin repeat containing | Plasma Membrane | other |
| 1.17E-05 | 1.716 | 10534303 | LAT2 | linker for activation of T cells family, member 2 | Plasma Membrane | other |
| 5.70E-06 | 2.974 | 10593449 | LAYN | layilin | Plasma Membrane | other |
| 2.30E-05 | -1.566 | 10583732 | LDLR | low density lipoprotein receptor | Plasma Membrane | transporter |
| 1.48E-04 | -2.075 | 10527012 | LFNG | LFNG O-fucosylpeptide 3-beta-N-acetylglucosaminyltransferase | Cytoplasm | enzyme |
| 2.10E-04 | -1.616 | 10422822 | LIFR | leukemia inhibitory factor receptor alpha | Plasma Membrane | transmembrane receptor |
| 5.79E-05 | -1.681 | 10561031 | LIPE | lipase, hormone-sensitive | Cytoplasm | enzyme |
| 2.65E-05 | -2.123 | 10435193 | LMLN | leishmanolysin-like (metallopeptidase M8 family) | Plasma Membrane | peptidase |
| 1.38E-04 | -1.522 | 10499394 | LMNA | lamin A/C | Nucleus | other |
| 8.50E-05 | -1.625 | 10541799 | LPAR5 | lysophosphatidic acid receptor 5 | Plasma Membrane | G-protein coupled receptor |
| 1.11E-05 | -1.933 | 10526656 | LRCH4 | leucine-rich repeats and calponin homology (CH) domain containing 4 | Cytoplasm | transcription regulator |
| 5.88E-08 | 2.839 | 10506714 | LRP8 | low density lipoprotein receptor-related protein 8, apolipoprotein e receptor | Plasma Membrane | transmembrane receptor |
| 2.71E-05 | -1.801 | 10408280 | LRRC16A | leucine rich repeat containing 16A | Cytoplasm | enzyme |
| 2.94E-05 | 1.594 | 10594221 | LRRC49 | leucine rich repeat containing 49 | unknown | other |
| 6.86E-05 | -1.594 | 10564377 | LRRK1 | leucine-rich repeat kinase 1 | Cytoplasm | kinase |
| 1.70E-04 | 1.538 | 10498576 | LXN | latexin | Cytoplasm | other |
| 2.88E-05 | 1.601 | 10360158 | LY9 | lymphocyte antigen 9 | Plasma Membrane | other |
| 1.31E-06 | -2.004 | 10484941 | MADD | MAP-kinase activating death domain | Cytoplasm | other |
| 4.87E-08 | -4.071 | 10581992 | MAF | v-maf musculoaponeurotic fibrosarcoma oncogene homolog (avian) | Nucleus | transcription regulator |
| 4.87E-06 | -1.581 | 10500847 | MAGI3 | membrane associated guanylate kinase, WW and PDZ domain containing 3 | Cytoplasm | kinase |
| 2.59E-04 | 1.601 | 10466712 | MAMDC2 | MAM domain containing 2 | Extracellular Space | other |
| 2.13E-06 | -2.015 | 10433008 | MAP3K12 | mitogen-activated protein kinase kinase kinase 12 | Cytoplasm | kinase |
| 1.58E-04 | -1.687 | 10447742 | MAP3K4 | mitogen-activated protein kinase kinase kinase 4 | Cytoplasm | kinase |
| 1.29E-05 | -2.939 | 10361926 | MAP3K5 | mitogen-activated protein kinase kinase kinase 5 | Cytoplasm | kinase |
| 2.32E-05 | 1.508 | 10414355 | MAPK1IP1L | mitogen-activated protein kinase 1 interacting protein 1-like | Nucleus | other |
| 5.81E-05 | 1.680 | 10572050 | MARCH1 | membrane-associated ring finger (C3HC4) 1, E3 ubiquitin protein ligase | Cytoplasm | enzyme |
| 7.38E-06 | -1.530 | 10463224 | MARVELD1 | MARVEL domain containing 1 | unknown | other |
| 6.10E-05 | -1.560 | 10579373 | MAST3 | microtubule associated serine/threonine kinase 3 | unknown | kinase |

| 3.87E-06 | 2.180 | 10546163 | MCM2 | minichromosome maintenance complex component 2 | Nucleus | enzyme |
| --- | --- | --- | --- | --- | --- | --- |
| 1.95E-04 | 1.735 | 10496771 | MCOLN2 | mucolipin 2 | Plasma Membrane | ion channel |
| 4.27E-05 | -3.597 | 10406434 | MEF2C | myocyte enhancer factor 2C | Nucleus | transcription regulator |
| 1.51E-04 | 2.601 | 10536505 | MET | met proto-oncogene (hepatocyte growth factor receptor) | Plasma Membrane | kinase |
| 3.56E-04 | -1.846 | 10554655 | MEX3B | mex-3 homolog B (C. elegans) | unknown | kinase |
| 2.63E-05 | -2.021 | 10354003 | MGAT4A | mannosyl (alpha-1,3-)-glycoprotein beta-1,4-N-acetylglucosaminyltransferase, isozyme A | Cytoplasm | enzyme |
| 8.76E-06 | 1.594 | 10362767 | MICAL1 | microtubule associated monoxygenase, calponin and LIM domain containing 1 | Cytoplasm | enzyme |
| 8.75E-05 | 1.582 | 10370021 | MIF | macrophage migration inhibitory factor (glycosylation-inhibiting factor) | Extracellular Space | cytokine |
| 1.58E-05 | 2.892 | 10405783 | mir-24 | microRNA 24-1 | Cytoplasm | microRNA |
| 3.46E-04 | -1.610 | 10430693 | MKL1 | megakaryoblastic leukemia (translocation) 1 | Nucleus | transcription regulator |
| 3.76E-05 | 2.073 | 10514255 | MLLT3 | myeloid/lymphoid or mixed-lineage leukemia (trithorax homolog, Drosophila); translocated to, 3 | Nucleus | other |
| 1.86E-05 | -2.169 | 10448582 | MLST8 | MTOR associated protein, LST8 homolog (S. cerevisiae) | Cytoplasm | other |
| 1.03E-04 | 1.624 | 10583090 | MMP10 | matrix metallopeptidase 10 (stromelysin 2) | Extracellular Space | peptidase |
| 7.22E-06 | 2.089 | 10583056 | MMP12 | matrix metallopeptidase 12 (macrophage elastase) | Extracellular Space | peptidase |
| 4.50E-06 | 2.911 | 10583044 | MMP13 | matrix metallopeptidase 13 (collagenase 3) | Extracellular Space | peptidase |
| 1.65E-05 | 1.692 | 10583112 | MMP27 | matrix metallopeptidase 27 | unknown | peptidase |
| 1.64E-06 | 1.858 | 10583100 | MMP8 | matrix metallopeptidase 8 (neutrophil collagenase) | Extracellular Space | peptidase |
| 3.03E-04 | -1.940 | 10512024 | MOB3B | MOB kinase activator 3B | unknown | other |
| 3.60E-06 | 1.865 | 10604630 | MOSPD1 | motile sperm domain containing 1 | unknown | other |
| 2.94E-04 | -1.917 | 10595981 | MRAS | muscle RAS oncogene homolog | Plasma Membrane | enzyme |
| 5.46E-07 | 2.328 | 10355456 | MREG | melanoregulin | Cytoplasm | other |
| 5.56E-07 | 1.848 | 10569542 | MRGPRE | MAS-related GPR, member E | Plasma Membrane | G-protein coupled receptor |
| 2.65E-05 | -1.651 | 10408348 | MRS2 | MRS2 magnesium homeostasis factor homolog (S. cerevisiae) | Cytoplasm | transporter |
| 2.00E-05 | 1.651 | 10466190 | MS4A14 | membrane-spanning 4-domains, subfamily A, member 14 | unknown | other |
| 1.70E-06 | 4.191 | 10363170 | Msl3l2 | male-specific lethal 3-like 2 (Drosophila) | unknown | other |
| 1.76E-05 | -1.707 | 10578916 | MSMO1 | methylsterol monooxygenase 1 | Cytoplasm | enzyme |
| 1.81E-04 | -1.549 | 10410264 | MTERFD1 | MTERF domain containing 1 | Cytoplasm | other |
| 4.38E-07 | -1.668 | 10553897 | MTMR10 | myotubularin related protein 10 | unknown | other |
| 3.16E-04 | -1.972 | 10494351 | MTMR11 | myotubularin related protein 11 | unknown | other |
| 2.79E-04 | 1.954 | 10410393 | MTRR | 5-methyltetrahydrofolate-homocysteine methyltransferase reductase | Cytoplasm | enzyme |
| 8.33E-06 | -1.957 | 10578324 | MTUS1 | microtubule associated tumor suppressor 1 | unknown | other |
| 8.74E-05 | -2.277 | 10507885 | MYCBP | c-myc binding protein | Nucleus | transcription regulator |
| 4.22E-05 | 1.756 | 10404874 | MYLIP | myosin regulatory light chain interacting protein | Cytoplasm | enzyme |
| 3.03E-06 | 1.538 | 10587107 | MYO5A | myosin VA (heavy chain 12, myoxin) | Cytoplasm | enzyme |
| 3.71E-04 | -1.733 | 10375886 | N4BP3 | NEDD4 binding protein 3 | Cytoplasm | other |
| 3.40E-05 | 1.534 | 10536595 | NAA38 | N(alpha)-acetyltransferase 38, NatC auxiliary subunit | Nucleus | other |
| 1.44E-05 | 1.520 | 10381211 | NAGLU | N-acetylglucosaminidase, alpha | Cytoplasm | enzyme |
| 4.17E-05 | 1.846 | 10395259 | NAMPT | nicotinamide phosphoribosyltransferase | Extracellular Space | cytokine |
| 4.03E-04 | 1.910 | 10464409 | NANOS1 | nanos homolog 1 (Drosophila) | Cytoplasm | translation regulator |
| 4.33E-05 | 2.004 | 10515337 | NASP | nuclear autoantigenic sperm protein (histone-binding) | Nucleus | other |
| 2.55E-04 | 1.780 | 10553354 | NAV2 | neuron navigator 2 | Nucleus | other |
| 1.38E-08 | -2.271 | 10597182 | NBEAL2 | neurobeachin-like 2 | Cytoplasm | other |
| 1.38E-07 | -1.925 | 10504703 | NCBP1 | nuclear cap binding protein subunit 1, 80kDa | Nucleus | other |
| 1.48E-05 | 1.984 | 10345913 | NCK2 | NCK adaptor protein 2 | Cytoplasm | kinase |

| 1.89E-04 | -2.154 | 10589113 | NCKIPSD | NCK interacting protein with SH3 domain | Nucleus | other |
| --- | --- | --- | --- | --- | --- | --- |
| 1.44E-05 | -1.523 | 10399265 | NCOA1 | nuclear receptor coactivator 1 | Nucleus | transcription regulator |
| 1.84E-05 | -1.530 | 10353135 | NCOA2 | nuclear receptor coactivator 2 | Nucleus | transcription regulator |
| 6.25E-07 | 2.946 | 10489620 | NCOA5 | nuclear receptor coactivator 5 | Nucleus | other |
| 9.20E-05 | -1.792 | 10533875 | NCOR2 | nuclear receptor corepressor 2 | Nucleus | transcription regulator |
| 1.18E-07 | 1.819 | 10429140 | NDRG1 | N-myc downstream regulated 1 | Nucleus | kinase |
| 1.01E-06 | -2.146 | 10353844 | NEURL3 | neuralized homolog 3 (Drosophila) pseudogene | unknown | other |
| 8.64E-05 | -1.635 | 10459944 | NFATC1 | nuclear factor of activated T-cells, cytoplasmic, calcineurin-dependent 1 | Nucleus | transcription regulator |
| 1.58E-05 | -2.778 | 10489961 | NFATC2 | nuclear factor of activated T-cells, cytoplasmic, calcineurin-dependent 2 | Nucleus | transcription regulator |
| 2.67E-05 | -1.760 | 10400405 | NFKBIA | nuclear factor of kappa light polypeptide gene enhancer in B-cells inhibitor, alpha | Cytoplasm | other |
| 1.77E-04 | -1.841 | 10445412 | NFKBIE | nuclear factor of kappa light polypeptide gene enhancer in B-cells inhibitor, epsilon | Nucleus | transcription regulator |
| 2.41E-05 | -1.647 | 10439936 | NFKBIZ | nuclear factor of kappa light polypeptide gene enhancer in B-cells inhibitor, zeta | Nucleus | transcription regulator |
| 4.53E-05 | 1.593 | 10601857 | NGFRAP1 | nerve growth factor receptor (TNFRSF16) associated protein 1 | Plasma Membrane | other |
| 1.03E-04 | 2.371 | 10517383 | NIPAL3 | NIPA-like domain containing 3 | unknown | other |
| 1.00E-07 | 1.768 | 10411882 | NLN | neurolysin (metallopeptidase M3 family) | Cytoplasm | peptidase |
| 8.81E-05 | -1.690 | 10452879 | NLRC4 | NLR family, CARD domain containing 4 | Cytoplasm | other |
| 1.10E-04 | -1.506 | 10376513 | NLRP3 | NLR family, pyrin domain containing 3 | Cytoplasm | other |
| 2.39E-04 | -1.807 | 10444352 | NOTCH4 | notch 4 | Plasma Membrane | transcription regulator |
| 2.03E-05 | 2.591 | 10581538 | NQO1 | NAD(P)H dehydrogenase, quinone 1 | Cytoplasm | enzyme |
| 5.51E-08 | -7.750 | 10427035 | NR4A1 | nuclear receptor subfamily 4, group A, member 1 | Nucleus | ligand-dependent nuclear receptor |
| 2.65E-04 | -1.827 | 10482772 | NR4A2 | nuclear receptor subfamily 4, group A, member 2 | Nucleus | ligand-dependent nuclear receptor |
| 4.62E-05 | 1.622 | 10568480 | NSMCE4A | non-SMC element 4 homolog A (S. cerevisiae) | Nucleus | other |
| 3.78E-05 | -1.697 | 10576561 | NTPCR | nucleoside-triphosphatase, cancer-related | unknown | other |
| 5.93E-06 | -3.400 | 10371379 | NUAK1 | NUAK family, SNF1-like kinase, 1 | unknown | kinase |
| 1.63E-06 | -1.862 | 10574033 | NUP93 | nucleoporin 93kDa | Nucleus | other |
| 1.04E-04 | -1.923 | 10520111 | NUPL2 | nucleoporin like 2 | Nucleus | transporter |
| 1.55E-06 | -2.873 | 10533198 | OAS2 | 2'-5'-oligoadenylate synthetase 2, 69/71kDa | Cytoplasm | enzyme |
| 1.45E-04 | 1.509 | 10354418 | OBFC2A | oligonucleotide/oligosaccharide-binding fold containing 2A | Nucleus | other |
| 8.46E-06 | 3.429 | 10582295 | ODC1 | ornithine decarboxylase 1 | Cytoplasm | enzyme |
| 1.50E-04 | -1.582 | 10470529 | OLFM1 | olfactomedin 1 | Cytoplasm | other |
| 2.51E-05 | -2.283 | 10548385 | OLR1 | oxidized low density lipoprotein (lectin-like) receptor 1 | Plasma Membrane | transmembrane receptor |
| 9.76E-06 | -1.960 | 10605884 | OPHN1 | oligophrenin 1 | Cytoplasm | other |
| 4.66E-05 | 2.295 | 10479833 | OPTN | optineurin | Cytoplasm | other |
| 6.78E-05 | -1.559 | 10525464 | ORAI1 | ORAI calcium release-activated calcium modulator 1 | Plasma Membrane | ion channel |
| 1.80E-07 | -2.214 | 10436426 | ORAI2 | ORAI calcium release-activated calcium modulator 2 | unknown | other |
| 3.45E-04 | 2.031 | 10457546 | OSBPL1A | oxysterol binding protein-like 1A | Cytoplasm | other |
| 2.33E-04 | -1.651 | 10380739 | OSBPL7 | oxysterol binding protein-like 7 | Cytoplasm | other |
| 6.16E-05 | -2.036 | 10517666 | OTUD3 | OTU domain containing 3 | unknown | other |
| 1.25E-05 | -1.765 | 10525419 | P2RX7 | purinergic receptor P2X, ligand-gated ion channel, 7 | Plasma Membrane | ion channel |
| 2.53E-05 | -1.597 | 10565958 | P2RY6 | pyrimidinergic receptor P2Y, G-protein coupled, 6 | Plasma Membrane | G-protein coupled receptor |
| 1.19E-04 | 1.782 | 10508974 | PAFAH2 | platelet-activating factor acetylhydrolase 2, 40kDa | Cytoplasm | enzyme |
| 2.62E-04 | -1.612 | 10555118 | PAK1 | p21 protein (Cdc42/Rac)-activated kinase 1 | Cytoplasm | kinase |
| 2.03E-05 | -2.454 | 10467162 | PANK1 | pantothenate kinase 1 | Cytoplasm | kinase |
| 8.15E-06 | 3.253 | 10590983 | PANX1 | pannexin 1 | Plasma Membrane | transporter |

| 3.44E-05 | -1.923 | 10531544 | PAQR3 | progestin and adipoQ receptor family member III | Cytoplasm | other |
| --- | --- | --- | --- | --- | --- | --- |
| 1.38E-06 | -2.248 | 10412345 | PARP8 | poly (ADP-ribose) polymerase family, member 8 | unknown | other |
| 1.17E-04 | 1.590 | 10486935 | PATL2 | protein associated with topoisomerase II homolog 2 (yeast) | Cytoplasm | translation regulator |
| 3.39E-05 | -1.995 | 10373728 | PATZ1 | POZ (BTB) and AT hook containing zinc finger 1 | Nucleus | transcription regulator |
| 3.09E-04 | 1.574 | 10596053 | PCCB | propionyl CoA carboxylase, beta polypeptide | Cytoplasm | enzyme |
| 1.76E-04 | -1.513 | 10468275 | PCGF6 | polycomb group ring finger 6 | Nucleus | transcription regulator |
| 4.64E-05 | -2.079 | 10468292 | PDCD11 | programmed cell death 11 | Nucleus | other |
| 1.09E-06 | -3.063 | 10556528 | PDE3B | phosphodiesterase 3B, cGMP-inhibited | Cytoplasm | enzyme |
| 1.59E-05 | -2.079 | 10506335 | PDE4B | phosphodiesterase 4B, cAMP-specific | Cytoplasm | enzyme |
| 3.76E-04 | -1.648 | 10497399 | PDE7A | phosphodiesterase 7A | Cytoplasm | enzyme |
| 1.37E-06 | -2.106 | 10430660 | PDGFB | platelet-derived growth factor beta polypeptide | Extracellular Space | growth factor |
| 4.06E-04 | 1.653 | 10511580 | PDP1 | pyruvate dehyrogenase phosphatase catalytic subunit 1 | Cytoplasm | phosphatase |
| 3.65E-05 | -1.750 | 10458334 | PFDN1 | prefoldin subunit 1 | Cytoplasm | transcription regulator |
| 1.73E-06 | -1.793 | 10383012 | PGS1 | phosphatidylglycerophosphate synthase 1 | Cytoplasm | enzyme |
| 9.73E-05 | -1.787 | 10378988 | PHF12 | PHD finger protein 12 | Nucleus | transcription regulator |
| 1.23E-05 | -1.899 | 10385747 | PHF15 | PHD finger protein 15 | Nucleus | other |
| 2.97E-05 | 2.707 | 10598848 | PHF16 | PHD finger protein 16 | Nucleus | other |
| 1.37E-04 | -1.666 | 10592891 | PHLDB1 | pleckstrin homology-like domain, family B, member 1 | Cytoplasm | other |
| 1.87E-06 | -1.750 | 10494509 | PIAS3 | protein inhibitor of activated STAT, 3 | Nucleus | transcription regulator |
| 1.43E-05 | 1.505 | 10567173 | PIK3C2A | phosphoinositide-3-kinase, class 2, alpha polypeptide | Cytoplasm | kinase |
| 2.60E-04 | -2.077 | 10518686 | PIK3CD | phosphoinositide-3-kinase, catalytic, delta polypeptide | Cytoplasm | kinase |
| 8.64E-06 | -2.845 | 10377286 | PIK3R6 | phosphoinositide-3-kinase, regulatory subunit 6 | Cytoplasm | kinase |
| 3.36E-04 | 1.634 | 10443527 | PIM1 (includes EG:18712) | pim-1 oncogene | Cytoplasm | kinase |
| 1.55E-05 | 1.639 | 10426110 | PIM3 | pim-3 oncogene | unknown | kinase |
| 2.16E-04 | -1.873 | 10415875 | PINX1 | PIN2/TERF1 interacting, telomerase inhibitor 1 | Nucleus | other |
| 2.46E-04 | -1.660 | 10392347 | PITPNC1 | phosphatidylinositol transfer protein, cytoplasmic 1 | Cytoplasm | transporter |
| 2.19E-05 | 2.882 | 10358434 | PLA2G4A | phospholipase A2, group IVA (cytosolic, calcium-dependent) | Cytoplasm | enzyme |
| 5.65E-06 | 2.596 | 10413047 | PLAU | plasminogen activator, urokinase | Extracellular Space | peptidase |
| 5.37E-05 | -1.597 | 10561431 | PLEKHG2 | pleckstrin homology domain containing, family G (with RhoGef domain) member 2 | Cytoplasm | other |
| 4.50E-07 | -2.927 | 10396671 | PLEKHG3 | pleckstrin homology domain containing, family G (with RhoGef domain) member 3 | unknown | other |
| 4.63E-05 | 1.692 | 10452030 | PLIN3 | perilipin 3 | Cytoplasm | other |
| 6.80E-06 | 1.564 | 10546184 | PLXNA1 | plexin A1 | Plasma Membrane | transmembrane receptor |
| 7.15E-05 | -2.292 | 10490159 | PMEPA1 | prostate transmembrane protein, androgen induced 1 | Plasma Membrane | other |
| 2.48E-06 | -2.564 | 10552760 | PNKP | polynucleotide kinase 3'-phosphatase | Nucleus | kinase |
| 2.26E-05 | -1.702 | 10577544 | POLB | polymerase (DNA directed), beta | Nucleus | enzyme |
| 6.59E-06 | -2.910 | 10400649 | POLE2 | polymerase (DNA directed), epsilon 2, accessory subunit | Nucleus | enzyme |
| 1.63E-04 | -1.806 | 10451860 | Pot1b | protection of telomeres 1B | Nucleus | other |
| 3.02E-07 | -2.235 | 10560964 | POU2F2 | POU class 2 homeobox 2 | Nucleus | transcription regulator |
| 1.61E-04 | -1.531 | 10432619 | POU6F1 | POU class 6 homeobox 1 | Nucleus | transcription regulator |
| 9.47E-06 | 6.094 | 10506488 | PPAP2B | phosphatidic acid phosphatase type 2B | Plasma Membrane | phosphatase |
| 2.36E-05 | 1.857 | 10542791 | PPFIBP1 | PTPRF interacting protein, binding protein 1 (liprin beta 1) | Plasma Membrane | other |
| 1.66E-07 | 1.958 | 10556082 | PPFIBP2 | PTPRF interacting protein, binding protein 2 (liprin beta 2) | Nucleus | phosphatase |
| 1.97E-05 | -2.045 | 10402730 | PPP1R13B | protein phosphatase 1, regulatory subunit 13B | Cytoplasm | phosphatase |

| 3.21E-04 | 1.885 | 10563338 | PPP1R15A | protein phosphatase 1, regulatory subunit 15A | Cytoplasm | other |
| --- | --- | --- | --- | --- | --- | --- |
| 1.14E-04 | -1.597 | 10447437 | PPP1R21 | protein phosphatase 1, regulatory subunit 21 | unknown | other |
| 6.89E-07 | 1.508 | 10507328 | PRDX1 | peroxiredoxin 1 | Cytoplasm | enzyme |
| 7.73E-06 | -2.386 | 10490632 | PRIC285 | peroxisomal proliferator-activated receptor A interacting complex 285 | Nucleus | transcription regulator |
| 2.78E-05 | -2.060 | 10589087 | PRKAR2A | protein kinase, cAMP-dependent, regulatory, type II, alpha | Cytoplasm | kinase |
| 4.81E-06 | 3.145 | 10399908 | PRKAR2B | protein kinase, cAMP-dependent, regulatory, type II, beta | Cytoplasm | kinase |
| 3.15E-05 | -1.858 | 10433797 | PRKDC | protein kinase, DNA-activated, catalytic polypeptide | Nucleus | kinase |
| 4.24E-05 | 2.069 | 10501485 | PRMT6 | protein arginine methyltransferase 6 | Nucleus | enzyme |
| 5.70E-07 | 1.699 | 10427235 | PRR13 | proline rich 13 | Nucleus | other |
| 3.80E-06 | 1.817 | 10500133 | PRUNE | prune homolog (Drosophila) | Nucleus | enzyme |
| 3.53E-04 | 1.629 | 10396193 | PSMA3 | proteasome (prosome, macropain) subunit, alpha type, 3 | Cytoplasm | peptidase |
| 1.10E-07 | -3.408 | 10427461 | PTGER4 | prostaglandin E receptor 4 (subtype EP4) | Plasma Membrane | G-protein coupled receptor |
| 1.06E-06 | 6.125 | 10513320 | PTGR1 | prostaglandin reductase 1 | Cytoplasm | other |
| 1.27E-04 | -1.723 | 10471721 | PTGS1 | prostaglandin-endoperoxide synthase 1 (prostaglandin G/H synthase and cyclooxygenase) | Cytoplasm | enzyme |
| 5.21E-06 | 1.554 | 10467489 | PTP4A1 | protein tyrosine phosphatase type IVA, member 1 | Cytoplasm | phosphatase |
| 2.95E-04 | 2.238 | 10478897 | PTPN1 | protein tyrosine phosphatase, non-receptor type 1 | Cytoplasm | phosphatase |
| 8.92E-06 | -1.620 | 10547769 | PTPN6 | protein tyrosine phosphatase, non-receptor type 6 | Cytoplasm | phosphatase |
| 1.04E-05 | -1.755 | 10524703 | PXN | paxillin | Cytoplasm | other |
| 1.21E-05 | -1.630 | 10568355 | PYCARD | PYD and CARD domain containing | Cytoplasm | transcription regulator |
| 1.71E-05 | -1.717 | 10577954 | RAB11FIP1 | RAB11 family interacting protein 1 (class I) | Cytoplasm | other |
| 8.13E-05 | -2.005 | 10587023 | RAB27A | RAB27A, member RAS oncogene family | Cytoplasm | enzyme |
| 8.71E-05 | 1.720 | 10352459 | RAB3GAP2 | RAB3 GTPase activating protein subunit 2 (non-catalytic) | Cytoplasm | enzyme |
| 2.31E-06 | 1.776 | 10596545 | RAD54L2 | RAD54-like 2 (S. cerevisiae) | Nucleus | transcription regulator |
| 6.73E-05 | 1.637 | 10479026 | RAE1 | RAE1 RNA export 1 homolog (S. pombe) | Nucleus | other |
| 1.85E-07 | 2.418 | 10547034 | RAF1 | v-raf-1 murine leukemia viral oncogene homolog 1 | Cytoplasm | kinase |
| 2.65E-05 | -1.773 | 10376568 | RAI1 | retinoic acid induced 1 | Cytoplasm | other |
| 1.06E-04 | -2.742 | 10466972 | RANBP6 | RAN binding protein 6 | Cytoplasm | other |
| 2.97E-06 | 2.389 | 10431974 | RAPGEF3 | Rap guanine nucleotide exchange factor (GEF) 3 | Nucleus | other |
| 1.91E-04 | -2.337 | 10432972 | RARG | retinoic acid receptor, gamma | Nucleus | ligand-dependent nuclear receptor |
| 2.16E-05 | 3.798 | 10572928 | RASD2 | RASD family, member 2 | Cytoplasm | enzyme |
| 1.75E-05 | 2.011 | 10541114 | RASGEF1A | RasGEF domain family, member 1A | unknown | other |
| 1.67E-04 | -1.716 | 10551696 | RASGRP4 | RAS guanyl releasing protein 4 | Cytoplasm | other |
| 1.38E-04 | -1.795 | 10459918 | RBFA | ribosome binding factor A (putative) | Cytoplasm | other |
| 1.68E-06 | -2.025 | 10489127 | RBL1 | retinoblastoma-like 1 (p107) | Nucleus | other |
| 1.97E-05 | -1.648 | 10598872 | RBM10 | RNA binding motif protein 10 | Nucleus | other |
| 4.19E-08 | -2.910 | 10440993 | RCAN1 | regulator of calcineurin 1 | Nucleus | transcription regulator |
| 1.94E-06 | 1.617 | 10585545 | RCN2 (includes EG:26611) | reticulocalbin 2, EF-hand calcium binding domain | Cytoplasm | other |
| 3.60E-06 | -3.070 | 10359762 | RCSD1 | RCSD domain containing 1 | unknown | other |
| 2.07E-04 | 1.510 | 10530130 | RELL1 | RELT-like 1 | unknown | other |
| 3.85E-04 | -1.744 | 10565924 | RELT | RELT tumor necrosis factor receptor | Plasma Membrane | transmembrane receptor |
| 5.73E-05 | 1.606 | 10526217 | RFC2 | replication factor C (activator 1) 2, 40kDa | Nucleus | other |
| 6.09E-05 | -1.582 | 10573295 | RFX1 (includes EG:100038773) | regulatory factor X, 1 (influences HLA class II expression) | Nucleus | transcription regulator |
| 2.20E-04 | -1.515 | 10606064 | RGAG4 | retrotransposon gag domain containing 4 | unknown | other |
| 6.24E-06 | 2.700 | 10358733 | RGL1 | ral guanine nucleotide dissociation stimulator-like 1 | Cytoplasm | other |
| 2.69E-05 | -1.650 | 10591668 | RGL3 | ral guanine nucleotide dissociation stimulator-like 3 | Cytoplasm | other |
| 1.90E-04 | -1.690 | 10405432 | RGS14 | regulator of G-protein signaling 14 | Cytoplasm | other |
| 1.54E-06 | -1.871 | 10358389 | RGS2 (includes EG:19735) | regulator of G-protein signaling 2, 24kDa | Nucleus | other |
| 3.49E-04 | 1.832 | 10562486 | RGS9BP | regulator of G protein signaling 9 binding protein | unknown | other |
| 1.58E-05 | -2.859 | 10363773 | RHOBTB1 | Rho-related BTB domain containing 1 | unknown | enzyme |
| 3.27E-05 | -2.036 | 10464754 | RHOD | ras homolog family member D | Cytoplasm | enzyme |
| 2.74E-04 | -2.046 | 10472058 | RIF1 (includes EG:295602) | RAP1 interacting factor homolog (yeast) | Nucleus | other |
| 2.89E-04 | 1.724 | 10533849 | RILPL1 | Rab interacting lysosomal protein-like 1 | Cytoplasm | other |
| 9.32E-05 | -1.701 | 10397853 | RIN3 | Ras and Rab interactor 3 | Cytoplasm | other |
| 1.86E-05 | 1.932 | 10511703 | RIPK2 | receptor-interacting serine-threonine kinase 2 | Plasma Membrane | kinase |
| 4.05E-04 | 1.805 | 10493309 | RIT1 (includes EG:19769) | Ras-like without CAAX 1 | Plasma Membrane | enzyme |
| 1.35E-04 | -1.530 | 10385699 | RMND5B | required for meiotic nuclear division 5 homolog B (S. cerevisiae) | unknown | other |
| 2.75E-05 | 1.600 | 10494527 | RNF115 | ring finger protein 115 | Cytoplasm | enzyme |
| 5.95E-05 | -1.979 | 10375343 | RNF145 | ring finger protein 145 | unknown | other |
| 2.23E-04 | -1.523 | 10383196 | RNF213 | ring finger protein 213 | Plasma Membrane | enzyme |
| 2.08E-06 | 2.394 | 10368577 | RNF217 | ring finger protein 217 | unknown | enzyme |
| 9.15E-05 | 1.509 | 10569057 | RNH1 | ribonuclease/angiogenin inhibitor 1 | Cytoplasm | other |
| 7.06E-05 | -1.598 | 10358038 | RNPEP | arginyl aminopeptidase (aminopeptidase B) | Cytoplasm | peptidase |
| 1.09E-04 | 2.093 | 10586700 | RORA | RAR-related orphan receptor A | Nucleus | ligand-dependent nuclear receptor |
| 9.82E-05 | 1.800 | 10479749 | RPP38 | ribonuclease P/MRP 38kDa subunit | Nucleus | enzyme |
| 4.37E-07 | 2.241 | 10361031 | RPS6KC1 | ribosomal protein S6 kinase, 52kDa, polypeptide 1 | Cytoplasm | kinase |
| 3.55E-06 | 1.594 | 10507894 | RRAGC | Ras-related GTP binding C | Cytoplasm | enzyme |
| 2.66E-05 | 6.423 | 10503835 | RRAGD | Ras-related GTP binding D | Cytoplasm | enzyme |
| 5.10E-06 | -2.071 | 10404612 | RREB1 | ras responsive element binding protein 1 | Nucleus | transcription regulator |
| 4.84E-05 | -1.972 | 10443836 | RRP1B | ribosomal RNA processing 1 homolog B (S. cerevisiae) | Nucleus | other |
| 5.76E-07 | -7.252 | 10434778 | RTP4 | receptor (chemosensory) transporter protein 4 | Plasma Membrane | other |
| 6.29E-05 | 1.790 | 10457118 | RTTN | rotatin | unknown | other |
| 3.04E-05 | -2.453 | 10509030 | RUNX3 | runt-related transcription factor 3 | Nucleus | transcription regulator |
| 3.81E-05 | 2.182 | 10413125 | SAMD8 | sterile alpha motif domain containing 8 | unknown | other |
| 3.81E-04 | -1.501 | 10440393 | SAMSN1 | SAM domain, SH3 domain and nuclear localization signals 1 | Nucleus | other |
| 2.42E-05 | -1.704 | 10599487 | SASH3 | SAM and SH3 domain containing 3 | Cytoplasm | other |
| 6.87E-05 | -1.993 | 10495574 | SASS6 | spindle assembly 6 homolog (C. elegans) | Cytoplasm | other |
| 2.21E-05 | 1.736 | 10533929 | SCARB1 | scavenger receptor class B, member 1 | Plasma Membrane | transporter |
| 3.16E-07 | -2.772 | 10463355 | Scd2 | stearoyl-Coenzyme A desaturase 2 | Cytoplasm | enzyme |
| 1.57E-04 | -1.785 | 10489484 | SDC4 | syndecan 4 | Plasma Membrane | other |
| 5.79E-05 | -2.203 | 10353899 | SEMA4C | sema domain, immunoglobulin domain (Ig), transmembrane domain (TM) and short cytoplasmic domain, (semaphorin) 4C | Plasma Membrane | other |
| 1.38E-05 | 2.560 | 10349118 | SERPINB12 | serpin peptidase inhibitor, clade B (ovalbumin), member 12 | Cytoplasm | other |
| 7.10E-05 | 1.543 | 10408600 | SERPINB6 | serpin peptidase inhibitor, clade B (ovalbumin), member 6 | Cytoplasm | other |
| 9.43E-08 | -2.803 | 10362811 | SESN1 | sestrin 1 | Nucleus | other |
| 3.83E-04 | -1.573 | 10574404 | SETD6 | SET domain containing 6 | Nucleus | enzyme |
| 1.35E-06 | 1.899 | 10469167 | SFMBT2 | Scm-like with four mbt domains 2 | unknown | other |
| 9.81E-05 | 1.607 | 10467068 | SGMS1 | sphingomyelin synthase 1 | Cytoplasm | enzyme |
| 3.26E-04 | 1.550 | 10348424 | SH3BP4 | SH3-domain binding protein 4 | Cytoplasm | other |
| 1.27E-07 | 2.011 | 10529497 | SH3TC1 | SH3 domain and tetratricopeptide repeats 1 | Extracellular Space | other |

| 1.18E-06 | 4.759 | 10358057 | SHISA4 | shisa homolog 4 (Xenopus laevis) | unknown | other |
| --- | --- | --- | --- | --- | --- | --- |
| 7.31E-06 | -2.243 | 10561247 | SHKBP1 | SH3KBP1 binding protein 1 | unknown | other |
| 3.15E-06 | -2.563 | 10449741 | SIK1 | salt-inducible kinase 1 | Cytoplasm | kinase |
| 4.56E-06 | -1.689 | 10465132 | SIPA1 | signal-induced proliferation-associated 1 | Cytoplasm | other |
| 6.22E-05 | -2.310 | 10397002 | SIPA1L1 | signal-induced proliferation-associated 1 like 1 | Cytoplasm | other |
| 1.48E-07 | -2.181 | 10582719 | SIPA1L2 | signal-induced proliferation-associated 1 like 2 | unknown | other |
| 2.70E-05 | -2.165 | 10393866 | SIRT7 | sirtuin 7 | Nucleus | enzyme |
| 5.04E-06 | -1.833 | 10429128 | SLA | Src-like-adaptor | Plasma Membrane | other |
| 1.86E-04 | 2.845 | 10495035 | SLC16A1 | solute carrier family 16, member 1 (monocarboxylic acid transporter 1) | Plasma Membrane | transporter |
| 4.84E-05 | 3.150 | 10356240 | SLC16A14 | solute carrier family 16, member 14 (monocarboxylic acid transporter 14) | unknown | other |
| 1.41E-10 | 4.538 | 10392440 | SLC16A6 | solute carrier family 16, member 6 (monocarboxylic acid transporter 7) | Plasma Membrane | transporter |
| 6.32E-07 | 12.898 | 10363860 | SLC16A9 | solute carrier family 16, member 9 (monocarboxylic acid transporter 9) | unknown | other |
| 5.24E-05 | 1.865 | 10351259 | SLC19A2 | solute carrier family 19 (thiamine transporter), member 2 | Plasma Membrane | transporter |
| 6.37E-06 | 2.366 | 10385893 | SLC22A4 | solute carrier family 22 (organic cation/ergothioneine transporter), member 4 | Plasma Membrane | transporter |
| 3.07E-04 | -2.582 | 10377372 | SLC25A35 | solute carrier family 25, member 35 | Cytoplasm | other |
| 1.36E-04 | -1.760 | 10421172 | SLC25A37 | solute carrier family 25 (mitochondrial iron transporter), member 37 | Cytoplasm | transporter |
| 2.19E-05 | 1.704 | 10507594 | SLC2A1 | solute carrier family 2 (facilitated glucose transporter), member 1 | Plasma Membrane | transporter |
| 2.62E-06 | -1.698 | 10386020 | SLC36A2 | solute carrier family 36 (proton/amino acid symporter), member 2 | Plasma Membrane | transporter |
| 2.67E-06 | 4.729 | 10354374 | SLC40A1 | solute carrier family 40 (iron-regulated transporter), member 1 | Plasma Membrane | transporter |
| 9.85E-07 | 2.644 | 10349711 | SLC41A1 | solute carrier family 41, member 1 | Plasma Membrane | transporter |
| 1.92E-05 | -2.047 | 10535841 | SLC46A3 | solute carrier family 46, member 3 | Extracellular Space | other |
| 1.69E-05 | 3.482 | 10535852 | SLC7A1 | solute carrier family 7 (cationic amino acid transporter, y+ system), member 1 | Plasma Membrane | transporter |
| 5.85E-06 | 4.230 | 10498024 | SLC7A11 | solute carrier family 7 (anionic amino acid transporter light chain, xc- system), member 11 | Plasma Membrane | transporter |
| 1.02E-04 | 1.676 | 10582275 | SLC7A5 | solute carrier family 7 (amino acid transporter light chain, L system), member 5 | Plasma Membrane | transporter |
| 2.17E-04 | -1.849 | 10574985 | SLC7A6 | solute carrier family 7 (amino acid transporter light chain, y+L system), member 6 | Plasma Membrane | transporter |
| 1.93E-05 | -2.967 | 10565819 | SLCO2B1 | solute carrier organic anion transporter family, member 2B1 | Plasma Membrane | transporter |
| 3.81E-04 | -1.766 | 10389143 | SLFN13 | schlafen family member 13 | Nucleus | enzyme |
| 5.04E-07 | -2.215 | 10568017 | SLX1A/SLX1B | SLX1 structure-specific endonuclease subunit homolog B (S. cerevisiae) | unknown | enzyme |
| 3.11E-04 | -1.552 | 10476301 | SMOX | spermine oxidase | Cytoplasm | enzyme |
| 1.02E-05 | -2.704 | 10390258 | SNX11 | sorting nexin 11 | unknown | transporter |
| 2.76E-04 | -1.607 | 10584034 | SNX19 | sorting nexin 19 | Cytoplasm | transporter |
| 1.93E-04 | 1.686 | 10433597 | SNX29 | sorting nexin 29 | unknown | other |
| 1.06E-05 | 2.473 | 10460118 | SOCS6 | suppressor of cytokine signaling 6 | Cytoplasm | other |
| 3.22E-04 | -1.514 | 10390308 | SP2 | Sp2 transcription factor | Nucleus | transcription regulator |
| 4.85E-06 | 2.220 | 10429856 | SPATC1 | spermatogenesis and centriole associated 1 | Cytoplasm | other |
| 9.59E-06 | 5.079 | 10483401 | SPC25 (includes EG:100144563) | SPC25, NDC80 kinetochore complex component, homolog (S. cerevisiae) | Cytoplasm | other |
| 1.02E-04 | 1.820 | 10586405 | SPG21 | spastic paraplegia 21 (autosomal recessive, Mast syndrome) | Plasma Membrane | enzyme |
| 2.11E-04 | 2.784 | 10598138 | SPRY3 | sprouty homolog 3 (Drosophila) | Plasma Membrane | other |

| 4.67E-05 | -1.888 | 10424349 | SQLE | squalene epoxidase | Cytoplasm | enzyme |
| --- | --- | --- | --- | --- | --- | --- |
| 2.41E-08 | 3.413 | 10477061 | SRXN1 | sulfiredoxin 1 | Cytoplasm | enzyme |
| 1.29E-04 | -2.034 | 10406551 | SSBP2 | single-stranded DNA binding protein 2 | Nucleus | transcription regulator |
| 7.37E-07 | -1.967 | 10506603 | SSBP3 | single stranded DNA binding protein 3 | Nucleus | transcription regulator |
| 3.20E-05 | -1.570 | 10378833 | SSH2 | slingshot homolog 2 (Drosophila) | Cytoplasm | phosphatase |
| 4.54E-05 | -2.014 | 10539080 | ST3GAL5 | ST3 beta-galactoside alpha-2,3-sialyltransferase 5 | Cytoplasm | enzyme |
| 1.13E-05 | 2.403 | 10566767 | ST5 | suppression of tumorigenicity 5 | unknown | enzyme |
| 1.63E-05 | -1.585 | 10367224 | STAT2 | signal transducer and activator of transcription 2, 113kDa | Nucleus | transcription regulator |
| 4.68E-07 | 2.180 | 10391301 | STAT3 | signal transducer and activator of transcription 3 (acute-phase response factor) | Nucleus | transcription regulator |
| 2.73E-04 | 1.600 | 10391286 | STAT5B | signal transducer and activator of transcription 5B | Nucleus | transcription regulator |
| 3.58E-05 | -1.523 | 10354588 | STK17B | serine/threonine kinase 17b | Nucleus | kinase |
| 1.13E-05 | 2.233 | 10513818 | STMN1 | stathmin 1 | Cytoplasm | other |
| 1.90E-04 | 1.595 | 10512443 | STOML2 | stomatin (EPB72)-like 2 | Plasma Membrane | other |
| 1.52E-05 | 1.796 | 10367919 | STX11 | syntaxin 11 | Plasma Membrane | transporter |
| 7.47E-08 | 3.086 | 10430596 | SUN2 | Sad1 and UNC84 domain containing 2 | Nucleus | other |
| 1.06E-04 | -1.524 | 10453636 | SVIL | supervillin | Plasma Membrane | other |
| 3.27E-04 | -1.597 | 10402490 | SYNE3 | spectrin repeat containing, nuclear envelope family member 3 | Nucleus | other |
| 2.02E-04 | -1.752 | 10441601 | TAGAP | T-cell activation RhoGTPase activating protein | Cytoplasm | other |
| 8.72E-06 | 1.516 | 10558903 | TALDO1 | transaldolase 1 | Cytoplasm | enzyme |
| 5.55E-06 | 1.951 | 10529858 | TAPT1 | transmembrane anterior posterior transformation 1 | Plasma Membrane | G-protein coupled receptor |
| 6.03E-06 | 1.530 | 10372457 | TBC1D15 | TBC1 domain family, member 15 | Cytoplasm | other |
| 5.50E-05 | 1.795 | 10512791 | TBC1D2 | TBC1 domain family, member 2 | Cytoplasm | other |
| 4.48E-05 | 1.534 | 10440050 | TBC1D23 | TBC1 domain family, member 23 | unknown | other |
| 1.68E-05 | -2.564 | 10354168 | TBC1D8 | TBC1 domain family, member 8 (with GRAM domain) | Plasma Membrane | other |
| 4.05E-04 | 1.672 | 10406710 | TBCA | tubulin folding cofactor A | Cytoplasm | other |
| 1.93E-04 | -1.766 | 10526302 | TBL2 | transducin (beta)-like 2 | Plasma Membrane | other |
| 3.84E-06 | 1.510 | 10592342 | TBRG1 | transforming growth factor beta regulator 1 | Nucleus | other |
| 3.78E-05 | 1.686 | 10594879 | TCF12 | transcription factor 12 | Nucleus | transcription regulator |
| 2.08E-04 | -2.094 | 10464084 | TCF7L2 | transcription factor 7-like 2 (T-cell specific, HMG-box) | Nucleus | transcription regulator |
| 2.22E-04 | 2.274 | 10365344 | TCP11L2 | t-complex 11 (mouse)-like 2 | unknown | other |
| 1.28E-05 | 1.785 | 10535532 | TECPR1 | tectonin beta-propeller repeat containing 1 | Cytoplasm | other |
| 8.78E-06 | -1.806 | 10504316 | TESK1 | testis-specific kinase 1 | Nucleus | kinase |
| 1.93E-04 | -1.623 | 10507347 | TESK2 | testis-specific kinase 2 | Nucleus | kinase |
| 4.80E-05 | 2.165 | 10543239 | TFEC | transcription factor EC | Nucleus | transcription regulator |
| 1.23E-04 | -2.147 | 10401673 | TGFB3 | transforming growth factor, beta 3 | Extracellular Space | growth factor |
| 2.41E-05 | -1.612 | 10405587 | TGFBI | transforming growth factor, beta-induced, 68kDa | Extracellular Space | other |
| 1.85E-04 | -1.913 | 10354233 | TGFBRAP1 | transforming growth factor, beta receptor associated protein 1 | Cytoplasm | other |
| 9.46E-05 | -1.650 | 10489204 | TGM2 (includes EG:21817) | transglutaminase 2 (C polypeptide, protein-glutamine-gamma-glutamyltransferase) | Cytoplasm | enzyme |
| 8.84E-06 | -2.063 | 10479136 | TH1L | TH1-like (Drosophila) | Nucleus | other |
| 6.11E-05 | -2.127 | 10539710 | TIA1 | TIA1 cytotoxic granule-associated RNA binding protein | Nucleus | other |
| 3.21E-05 | -2.144 | 10440738 | TIAM1 | T-cell lymphoma invasion and metastasis 1 | Cytoplasm | other |
| 3.28E-06 | -2.010 | 10409567 | TIFAB | TRAF-interacting protein with forkhead-associated domain, family member B | unknown | other |

| 4.03E-06 | 2.801 | 10379034 | TLCD1 | TLC domain containing 1 | unknown | other |
| --- | --- | --- | --- | --- | --- | --- |
| 2.38E-05 | -1.625 | 10513884 | TLE1 | transducin-like enhancer of split 1 (E(sp1) homolog, Drosophila) | Nucleus | transcription regulator |
| 1.05E-05 | -1.986 | 10498992 | TLR2 | toll-like receptor 2 | Plasma Membrane | transmembrane receptor |
| 9.69E-06 | 2.289 | 10435043 | TM4SF19 | transmembrane 4 L six family member 19 | unknown | other |
| 2.17E-04 | 2.013 | 10382956 | TMC8 | transmembrane channel-like 8 | unknown | other |
| 3.99E-06 | 3.307 | 10509601 | TMCO4 | transmembrane and coiled-coil domains 4 | unknown | other |
| 2.32E-04 | -1.764 | 10411459 | TMEM171 | transmembrane protein 171 | unknown | other |
| 1.08E-05 | -2.300 | 10458314 | TMEM173 | transmembrane protein 173 | Cytoplasm | other |
| 2.81E-04 | 1.618 | 10594177 | TMEM202 | transmembrane protein 202 | unknown | other |
| 2.13E-05 | -1.715 | 10363161 | TMEM229B | transmembrane protein 229B | unknown | other |
| 2.52E-05 | -1.780 | 10357239 | TMEM37 | transmembrane protein 37 | Plasma Membrane | ion channel |
| 4.52E-06 | 1.593 | 10505064 | TMEM38B | transmembrane protein 38B | Nucleus | ion channel |
| 1.08E-04 | 1.583 | 10587746 | TMEM41B | transmembrane protein 41B | unknown | other |
| 2.59E-04 | 1.646 | 10429114 | TMEM71 | transmembrane protein 71 | unknown | other |
| 6.80E-05 | 1.714 | 10434668 | TMEM97 | transmembrane protein 97 | Extracellular Space | other |
| 1.85E-04 | -1.928 | 10455647 | TNFAIP8 | tumor necrosis factor, alpha-induced protein 8 | Cytoplasm | other |
| 2.33E-05 | 2.486 | 10569485 | Tnfrsf26 | tumor necrosis factor receptor superfamily, member 26 | unknown | other |
| 7.11E-05 | -1.843 | 10538791 | TNIP3 | TNFAIP3 interacting protein 3 | unknown | other |
| 1.78E-06 | 2.948 | 10355534 | TNS1 | tensin 1 | Plasma Membrane | other |
| 1.68E-04 | -1.528 | 10478196 | TOP1 | topoisomerase (DNA) I | Nucleus | enzyme |
| 2.91E-04 | 1.559 | 10386582 | TOP3A | topoisomerase (DNA) III alpha | Nucleus | enzyme |
| 1.73E-04 | -1.834 | 10480601 | TOR4A | torsin family 4, member A | unknown | other |
| 1.85E-04 | 1.717 | 10478364 | TOX2 | TOX high mobility group box family member 2 | Nucleus | transcription regulator |
| 1.12E-06 | 1.875 | 10503259 | TP53INP1 | tumor protein p53 inducible nuclear protein 1 | Nucleus | other |
| 8.86E-05 | -1.691 | 10477644 | TP53INP2 | tumor protein p53 inducible nuclear protein 2 | Nucleus | other |
| 4.43E-07 | 1.524 | 10572747 | Tpm4 | tropomyosin 4 | Cytoplasm | other |
| 2.17E-04 | -2.510 | 10445746 | TREM1 | triggering receptor expressed on myeloid cells 1 | Plasma Membrane | other |
| 9.73E-06 | -2.275 | 10424370 | TRIB1 | tribbles homolog 1 (Drosophila) | Cytoplasm | kinase |
| 1.06E-04 | 1.968 | 10415784 | TRIM13 | tripartite motif containing 13 | Cytoplasm | enzyme |
| 3.76E-04 | 1.808 | 10376899 | TRIM16 | tripartite motif containing 16 | Cytoplasm | transcription regulator |
| 5.10E-07 | 3.523 | 10555848 | TRIM6 | tripartite motif containing 6 | Cytoplasm | other |
| 3.09E-07 | -2.019 | 10393113 | TRIM65 | tripartite motif containing 65 | unknown | other |
| 4.03E-05 | -1.647 | 10398717 | TRMT61A | tRNA methyltransferase 61 homolog A (S. cerevisiae) | unknown | enzyme |
| 3.44E-06 | 2.923 | 10532839 | TRPV4 | transient receptor potential cation channel, subfamily V, member 4 | Plasma Membrane | ion channel |
| 7.09E-05 | -1.613 | 10376839 | TTC19 | tetratricopeptide repeat domain 19 | Cytoplasm | other |
| 3.49E-04 | -2.442 | 10514128 | TTC39B | tetratricopeptide repeat domain 39B | unknown | other |
| 4.42E-07 | -2.351 | 10478401 | TTPAL | tocopherol (alpha) transfer protein-like | unknown | other |
| 8.53E-05 | 1.504 | 10382376 | TTYH2 | tweety homolog 2 (Drosophila) | unknown | ion channel |
| 1.30E-04 | 1.833 | 10500011 | TUFT1 | tuftelin 1 | Extracellular Space | other |
| 4.23E-05 | 2.394 | 10365260 | TXNRD1 | thioredoxin reductase 1 | Cytoplasm | enzyme |
| 5.00E-05 | -2.543 | 10592515 | UBASH3B | ubiquitin associated and SH3 domain containing B | unknown | enzyme |
| 4.38E-05 | 1.568 | 10535866 | UBL3 | ubiquitin-like 3 | Cytoplasm | other |
| 6.38E-05 | -1.505 | 10537375 | UBN2 | ubinuclein 2 | Nucleus | other |
| 6.16E-05 | -1.532 | 10589848 | UBP1 (includes EG:100136855) | upstream binding protein 1 (LBP-1a) | Cytoplasm | transcription regulator |
| 3.95E-06 | -1.718 | 10481577 | UCK1 | uridine-cytidine kinase 1 | Cytoplasm | kinase |

| 7.72E-05 | 1.688 | 10399036 | UEVLD | UEV and lactate/malate dehyrogenase domains | Cytoplasm | enzyme |
| --- | --- | --- | --- | --- | --- | --- |
| 4.88E-05 | -1.543 | 10526564 | UFSP1 | UFM1-specific peptidase 1 (non-functional) | unknown | enzyme |
| 1.55E-05 | -2.227 | 10585732 | ULK3 | unc-51-like kinase 3 (C. elegans) | Cytoplasm | kinase |
| 9.42E-05 | 2.051 | 10369388 | UNC5B | unc-5 homolog B (C. elegans) | Plasma Membrane | transmembrane receptor |
| 2.01E-05 | 1.593 | 10386636 | USP22 | ubiquitin specific peptidase 22 | Nucleus | peptidase |
| 9.18E-06 | -1.579 | 10545379 | USP39 | ubiquitin specific peptidase 39 | Nucleus | peptidase |
| 1.72E-06 | -1.799 | 10530641 | USP46 | ubiquitin specific peptidase 46 | unknown | peptidase |
| 4.08E-04 | -2.076 | 10367744 | UST | uronyl-2-sulfotransferase | Cytoplasm | enzyme |
| 8.44E-05 | -1.557 | 10565759 | UVRAG | UV radiation resistance associated gene | Nucleus | other |
| 3.93E-04 | -1.541 | 10397450 | VASH1 | vasohibin 1 | Extracellular Space | other |
| 1.13E-04 | -1.535 | 10481210 | VAV2 | vav 2 guanine nucleotide exchange factor | Cytoplasm | other |
| 3.56E-05 | -2.924 | 10432032 | VDR | vitamin D (1,25- dihydroxyvitamin D3) receptor | Nucleus | ligand-dependent nuclear receptor |
| 2.29E-05 | 1.584 | 10465559 | Vegfb | vascular endothelial growth factor B | Extracellular Space | other |
| 1.60E-04 | -1.668 | 10570771 | VPS36 (includes EG:290851) | vacuolar protein sorting 36 homolog (S. cerevisiae) | Cytoplasm | other |
| 1.16E-04 | 1.917 | 10533729 | VPS37B | vacuolar protein sorting 37 homolog B (S. cerevisiae) | Cytoplasm | other |
| 1.97E-07 | 1.799 | 10541910 | VWF | von Willebrand factor | Extracellular Space | other |
| 6.24E-06 | 2.363 | 10526232 | WBSCR27 | Williams Beuren syndrome chromosome region 27 | unknown | enzyme |
| 2.08E-04 | 1.585 | 10581835 | WDR59 | WD repeat domain 59 | unknown | transporter |
| 4.43E-05 | 1.619 | 10388451 | WDR81 | WD repeat domain 81 | unknown | other |
| 2.00E-04 | -1.548 | 10469856 | WDR85 | WD repeat domain 85 | unknown | other |
| 3.66E-04 | -1.616 | 10521136 | WHSC1 | Wolf-Hirschhorn syndrome candidate 1 | Nucleus | enzyme |
| 3.96E-06 | -2.831 | 10378068 | XAF1 | XIAP associated factor 1 | Nucleus | other |
| 2.34E-09 | 5.229 | 10374590 | XPO1 | exportin 1 (CRM1 homolog, yeast) | Nucleus | transporter |
| 7.52E-05 | -1.560 | 10390059 | XYLT2 | xylosyltransferase II | unknown | enzyme |
| 4.74E-05 | 1.922 | 10406733 | ZBED3 | zinc finger, BED-type containing 3 | Cytoplasm | other |
| 2.28E-05 | -1.836 | 10377593 | ZBTB4 | zinc finger and BTB domain containing 4 | Nucleus | other |
| 6.71E-06 | 1.704 | 10593497 | ZC3H12C | zinc finger CCCH-type containing 12C | unknown | other |
| 1.05E-06 | -2.901 | 10418171 | ZCCHC24 | zinc finger, CCHC domain containing 24 | unknown | other |
| 5.11E-06 | 1.829 | 10517070 | ZDHHC18 | zinc finger, DHHC-type containing 18 | unknown | enzyme |
| 8.72E-05 | -1.610 | 10439542 | ZDHHC23 | zinc finger, DHHC-type containing 23 | unknown | other |
| 1.01E-04 | 1.543 | 10461991 | ZFAND5 | zinc finger, AN1-type domain 5 | Nucleus | other |
| 7.69E-07 | 5.316 | 10442224 | Zfp948 | zinc finger protein 948 | unknown | other |
| 1.50E-05 | -1.662 | 10576090 | ZFPM1 | zinc finger protein, multitype 1 | Nucleus | transcription regulator |
| 1.98E-05 | 1.665 | 10411059 | ZFYVE16 | zinc finger, FYVE domain containing 16 | Nucleus | transporter |
| 5.60E-05 | -1.639 | 10377826 | ZMYND15 | zinc finger, MYND-type containing 15 | unknown | other |
| 9.86E-05 | -1.735 | 10412421 | ZNF131 | zinc finger protein 131 | Nucleus | transcription regulator |
| 2.25E-04 | -1.857 | 10408049 | ZNF192 | zinc finger protein 192 | Nucleus | transcription regulator |
| 2.64E-04 | -1.955 | 10490053 | ZNF217 | zinc finger protein 217 | Nucleus | transcription regulator |
| 5.18E-06 | -2.032 | 10433199 | ZNF263 | zinc finger protein 263 | Nucleus | transcription regulator |
| 2.77E-06 | -1.796 | 10441330 | ZNF295 | zinc finger protein 295 | Nucleus | other |
| 7.77E-06 | -1.678 | 10415991 | ZNF395 | zinc finger protein 395 | Cytoplasm | other |
| 7.47E-06 | -2.244 | 10444008 | ZNF414 | zinc finger protein 414 | unknown | other |
| 1.30E-05 | -2.170 | 10442549 | ZNF598 | zinc finger protein 598 | Extracellular Space | other |
| 2.12E-04 | -1.980 | 10515986 | ZNF642 | zinc finger protein 642 | unknown | other |
| 1.74E-04 | -1.725 | 10365208 | ZNF845 | zinc finger protein 845 | Nucleus | other |
| 1.75E-04 | -2.034 | 10478590 | ZSWIM1 | zinc finger, SWIM-type containing 1 | Nucleus | other |
| 2.46E-04 | 1.545 | 10363905 | ZWINT | ZW10 interactor | Nucleus | other |

Supplementary Table 2: genes affected by SMA12b that are of possible relevance to RA

| Gene ID | Gene Name | Fold change | Type/Location | Role in macrophage/arthritis |
| --- | --- | --- | --- | --- |
| **TLR and TLR/IL-1R-specific signaling** | | | | |
| 10498992 | TLR2  Toll-like receptor 2 | **-1.99** | Transmembrane receptor/Plasma membrane | Persistent TLR2 signalling is observed in the pathogenesis of RA. Endogenous ligands in RA e.g. SNAPIN bind to TLR2 leading to persistent activation of macrophages. |
| 10397346 | c-Fos  FBJ murine osteosarcoma viral oncogene homolog | **-3.1** | Transcription regulator/Nucleus | Target of NF-κB in RANKL-induced osteoclastogenesis. |
| 10487597 | IL-1  Interleukin 1 | **-8.9** | Cytokine/Extracellular space | Plays an important role in several chronic inflammatory diseases, including RA; recombinant human interleukin-1 receptor antagonist (rhIL-1ra), Anakinra, has been approved for the treatment of patients with moderate-severe RA; two models of autoimmune arthritis, collagen-induced arthritis (CIA) and antigen-induced arthritis (AIA), are known to be highly dependent on IL-1. |
| 10376060 | IRF-1  interferon regulatory factor 1 | **-1.6** | Transcription regulator/Nucleus | Induces type I IFNs and effector genes. Its target genes for transcriptional activation include genes involved in chronic inflammatory responses, such as IFN-α/β. |
| 10587495 | IRAK1BP1  Interleukin-1 receptor-associated kinase 1 binding protein 1 | **2.3** | Unknown/Other | IRAK1BP1 inhibits inflammation by promoting nuclear translocation of NF-κB p50. |
| **Inflammasome** | | | | |
| 10452879 | NLRC4  NLR family, CARD domain containing 4 | **-1.7** | Other/Cytoplasm | Member of NOD family that recognises bacterial flagellin; is a target for PKCδ-mediated activation of NLRC4/caspase-1 and IL-1β secretion from macrophages. |
| 10376513 | NLRP3  NLR family, pyrin domain containing 3 | **-1.5** | Other/Cytoplasm | Encodes a pyrin-like protein that interacts with ASC for NF-κB signalling. Mutations in this gene have been associated with a spectrum of dominantly inherited [autoinflammatory diseases](http://en.wikipedia.org/wiki/Autoinflammatory_disease) called [cryopyrin-associated periodic syndrome](http://en.wikipedia.org/wiki/Cryopyrin-associated_periodic_syndrome) (CAPS). |
| 10568355 | PYCARD  PYD and CARD domain containing | **-1.6** | Transcription regulator/Cytoplasm | Encodes an adaptor protein that is composed of two protein–protein interaction domains: a [N-terminal](http://en.wikipedia.org/wiki/N-terminus) PYRIN-PAAD-DAPIN domain (PYD) and a [C-terminal](http://en.wikipedia.org/wiki/C-terminus) caspase-recruitment domain ([CARD](http://en.wikipedia.org/wiki/CARD_domain)). PYCARD has been shown to [interact](http://en.wikipedia.org/wiki/Protein–protein_interaction) with [MEFV](http://en.wikipedia.org/wiki/MEFV). |
| 10357604 | IKBKE (IKK-e)  inhibitor of kappa light polypeptide gene enhancer in B-cells, kinase epsilon | **-1.6** | Kinase/Cytoplasm | Overexpression of IKKɛ induces phosphorylation of TRAF/TANK, which results in its dissociation from TRAF2 and subsequent activation of NF-κB transcription through the classical IKK pathway. |
|  |  |  |  |  |
| 10538791 | TNIP3  TNFAIP3 interacting protein 3 | **-1.8** | Other/Unknown | Overexpression inhibits NF-κB-dependent gene expression in response to LPS at a level downstream of TRAF6 and upstream of IKBKB. NF-κB inhibition is independent of TNFAIP3 binding. |
| **Immunoreceptors** | | | | |
|  |  |  |  |  |
| 10524703 | PXN  Paxillin | **-1.8** | Other/Cytoplasm |  |
| 10583519 | ICAM1  intercellular adhesion molecule 1 | **-1.7** | Transmembrane receptor/Plasma membrane | Cell adhesion molecules |
| 10364361 | ICOSLG  inducible T-cell co-stimulator ligand | **-3.3** | Other/Plasma membrane | Co-stimulation of T cells |
| 10445746 | TREM1  triggering receptor expressed on myeloid cells 1 | **-2.5** | Other/Plasma membrane | Amplifies neutrophil and monocyte-mediated inflammatory responses triggered by bacterial and fungal infections by stimulating release of pro-inflammatory chemokines and cytokines, as well as increased surface expression of cell activation markers. |
| 10435704 | CD80  CD80 molecule | **-1.7** | Transmembrane receptor/Plasma membrane | costimulation |
| 10544273 | CLEC5A  C-type lectin domain family 5, member A | **-1.5** | Other/Plasma membrane | key regulator of synovial injury and bone erosion during autoimmune joint inflammation. |
| 10557342 | IL21R  interleukin 21 receptor | **-2.4** | Transmembrane receptor/Plasma membrane |  |
| 10560242 | C5AR1  complement component 5a receptor 1 | **-4.2** | G-protein coupled receptor/Plasma membrane | C5a exerts a predominant pro-inflammatory activity through interactions with C5ar1; the group of therapeutic compounds that targets C5aR represents one of the largest developing groups of complement therapeutics. |
| 10435907 | CD200R1  CD200 receptor 1 | **2.6** | Other/Plasma membrane | Thought to play an inhibitory role in immunity; the severity of arthritis was increased significantly in CD200R1(-/-) mice compared to wild-type mice. |
| 10462390 | CD274  CD274 molecule | **2.9** | Enzyme/Plasma membrane | Programmed cell death 1 ligand 1 (PD-L1) also known as cluster of differentiation (CD274) or B7 homolog 1 (B7-H1); thought to mediate T cell-negative costimulation. |
| **Chemokine and chemokine receptors** | | | | |
| 10597743 | CX3CR1  chemokine (C-X3-C motif) receptor 1 | **-3.7** | G-protein coupled receptor /Plasma Membrane | Important players in the trafficking of monocytes/macrophages and in the functions of other cell types relevant to disease pathogenesis; CCR2 and CCR5 are important players in preclinical models of RA, mice treated with a small molecule antagonist of mouse CCR2, CCR5, and CXCR3 (TAK-779) were protected from CIA;  CX3CR1 is a chemokine receptor that uniquely binds to its ligand fractalkine (CX(3) CL1) and has been shown to be important in inflammatory arthritis responses, largely due to its effects on cellular migration; CX3CR1 deficiency is protective in inflammatory arthritis. |
| 10590631 | Ccr2  chemokine (C-C motif) receptor 2 | **-3.8** | G-protein coupled receptor/Plasma Membrane |  |
| 10590635 | Ccr5  chemokine (C-C motif) receptor 5 (gene/pseudogene) | **-5.8** | G-protein coupled receptor/Plasma Membrane |  |
| 10523156 | CXCL3 chemokine (C-X-C motif) ligand 3 | **-1.98** | Cytokine/Extracellular space | Binds to its receptor CXCR3 and thus contributes to the recruitment of T cells from the blood stream into the inflamed joints and have a crucial role in perpetuating inflammation in RA synovial joints. |
| 10531415 | CXCL10  chemokine (C-X-C motif) ligand 10 | **-4.3** | Cytokine/Extracellular space | CXCL10 and its receptor, CXCR3, are increased in many kinds of chronic inflammatory arthritis, especially in RA. |
| 10409579 | CXCL14 chemokine (C-X-C motif) ligand 14 | **-1.7** | Cytokine/Extracellular space | Overexpression exacerbates collagen-induced arthritis. |
| **NFAT signalling & Osteoclastogenesis** | | | | |
| 10459944 | NFATC1 nuclear factor of activated T-cells, cytoplasmic, calcineurin-dependent 1 | **-1.6** | Transcription regulator/Nucleus | The NFAT transcription factor family consists of five members [NFATc1](http://en.wikipedia.org/wiki/NFATC1), [NFATc2](http://en.wikipedia.org/wiki/NFATC2), [NFATc3](http://en.wikipedia.org/wiki/NFATC3), [NFATc4](http://en.wikipedia.org/wiki/NFATC4), and [NFAT5](http://en.wikipedia.org/wiki/NFAT5);  NF-κB has been shown to activate c-Fos and NFATc1 to induce expression of cathepsin K, TRAP and other genes involved in osteoclast resorptive functions;  NFATc1 is the master regulator of this process and in its absence osteoclast differentiation is aborted both in vitro and in vivo. |
| 10489961 | NFATC2 (NFAT1)  nuclear factor of activated T-cells, cytoplasmic, calcineurin-dependent 2 | **-2.8** | Transcription regulator/Nucleus |  |
| 10397346 | FOS  FBJ murine osteosarcoma viral oncogene homolog | **-3.1** | Transcription regulator/Nucleus |  |
| **Anti-oxidant stress responses** | | | | |
| 10587266 | GCLC  glutamate-cysteine ligase, catalytic subunit  also known as g-GCS=g-glutamylcysteine synthetase | **3.14** | Cytoplasm/Enzyme | Required for *de novo* synthesis of GSH. |
| 10488879 | GSS  glutathione synthetase | **3.24** | Cytoplasm/Enzyme | Required for *de novo* synthesis of GSH. |
| 10571274 | GSR or GR  Glutathione reductase | **2.08** | Cytoplasm/Enzyme | Catalizes reduction of GSSG in the process that requires NADPH; required for the conversion of oxidized glutathione (GSSG) to reduced glutathione (GSH). Glutathione reductase therefore plays a major role in glutathione peroxidase (GPx) and glutathione s-transferase (GST) reactions as an adjunct in the control of peroxides and free radicals. |
| 10365260 | TXNRDI  thioredoxin reductase 1 | **2.39** | Cytoplasm/Enzyme | thierodoxin reductase are the only known enzymes to reduce [thioredoxin](http://en.wikipedia.org/wiki/Thioredoxin). Thioredoxins, cysteine and reduced glutathione are examples of molecules that can reduce free radicals by hydrogen donation. Functionally homologous to glutathione reductase. |
| 10507328 | PRDX1  peroxiredoxin 1 | **1.50** | Cytoplasm/Enzyme | Known as GSSG reductase. |
| 10572897 | HMOX1  heme oxygenase (decycling) 1 | **3.58** | Cytoplasm/Enzyme | The induction of heme oxygenase-1 (HO-1; Hmox1) by inflammation, for instance in sepsis, is associated both with an anti-inflammatory response and with mitochondrial biogenesis. |

**Supplemental Fig.1. SMA-12b and -19o do not induce bmM cell death.** (A) Structures of SMA-11a, 12b and 19o. (B) bmM were plated on ultra low binding tissue culture plates in RPMI complete medium and rested for 24h before culturing with 12b or 19o (both 5 µg/ml) in fresh medium for 18h. Subsequently, the macrophages were stimulated with medium, LPS (100 ng/ml), BLP (10 ng/ml) or CpG (0.01 µM) for an additional 24h and the cells were then stained with 7-AAD to assess thier viability after exposure to the SMAs for 42h. The samples were analysed by flow cytometry and the data are presented as density plots with frequenct of 7-AAD positive cells indicated in the gates. The results shown are representative of two independent experiments.
